# Hybrid Antibiotics Targeting the Bacterial Ribosome

**DOI:** 10.1021/acscentsci.5c01046

**Published:** 2025-09-17

**Authors:** Seul Ki Yeon, Jenna Pellegrino, Tushar Raskar, Minh L. N. Tran, Mohamad Dandan, François Guérin, Manuel Einsiedler, Vincent Cattoir, James S. Fraser, Ian B. Seiple

**Affiliations:** † Department of Pharmaceutical Chemistry, Cardiovascular Research Institute, University of California—San Francisco, San Francisco, California 94158, United States; ‡ Department of Bioengineering and Therapeutic Sciences, 8785 University of California—San Francisco, San Francisco, California 94158, United States; § Department of Clinical Microbiology, 36684 University Hospital of Rennes, FR-35033 Rennes, France; ∥ National Reference Center for Antimicrobial Resistance (lab Enterococci), FR-35033 Rennes, France; ⊥ Inserm U1230 BRM unit, University of Rennes, FR-35043 Rennes, France; # Department of Chemistry, 4356 The Scripps Research Institute, La Jolla, California 92037, United States

## Abstract

Antimicrobial resistance remains a formidable challenge to modern medicine, with bacterial resistance mechanisms increasingly eroding the utility of clinically important antibiotics. While recent efforts have expanded the antibacterial pipeline, the development of resistance in priority pathogens continues to exceed the pace of new drug development. One emerging strategy to overcome resistance is the rational design of hybrid antibiotics that engage multiple binding sites. Here we describe the design, synthesis, and microbiological and structural characterization of hybrid antibiotics of azithromycin, tedizolid, and chloramphenicol that span the peptidyltransferase center (PTC) and nascent peptide exit tunnel (NPET) in the bacterial ribosome. We characterize the binding of four such hybrids by cryo-electron microscopy, granting insight into their molecular mechanisms of action. We identify a hybrid of azithromycin and tedizolid that is active against a diverse panel of multidrug-resistant Gram-positive bacteria and is minimally affected by ribosomal protection (ABC-F) resistance mechanisms. These results extend our understanding of ribosome inhibition and provide a pipeline for the rational design of dual-action antibiotics that target the ribosome. In a broader context, this work offers a framework for developing bifunctional inhibitors that engage adjacent binding sites by means of a rational cycle of synthetic optimization, biological evaluation, and structural characterization.

## Graphical abstract

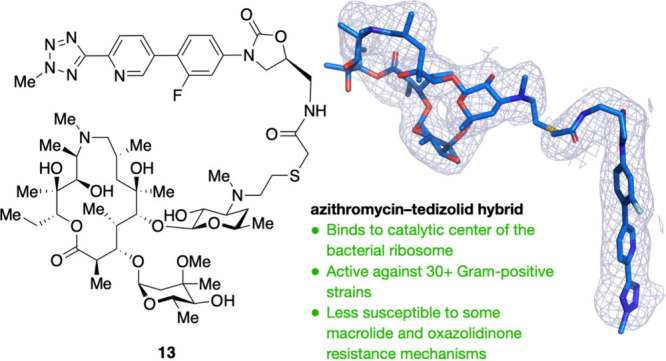

## Introduction

Antimicrobial resistance (AMR) is a growing threat to modern medicine. It is estimated that bacterial AMR was directly responsible for 1.27 million deaths and was associated with 4.95 million deaths in 2019.^1^ Despite a recent increase in the number of antibacterials in the clinical pipeline, 2024 analyses by the World Health Organization show that resistance in priority pathogens continues to outpace therapeutic development.^2,3^ The bacterial ribosome is the target of many clinically important classes of antibiotics.^4^ Several of these ribosome inhibitors, including oxazolidinones, lincosamides, pleuromutilins, phenicols, streptogramins, and macrolides, bind to the peptidyl transferase center (PTC) or the adjacent nascent peptide exit tunnel (NPET). Owing to advances in X-ray crystallography^4^ and cryo-electron microscopy (cryo-EM),^5^ the binding modes of these classes to the PTC and NPET have been elucidated in atomic detail. These data reveal the molecular mechanisms of action of these drugs and enable structure-based drug design to further optimize their properties, as recently exemplified by the synthesis of improved macrolides,^6^ streptogramins,^7^ and lincosamides.^8,9^

Several ribosome-related resistance mechanisms confer resistance to antibiotics that bind to the PTC, the NPET, or both. Binding site modifications such as methylation or mutation of A2503 (PTC) or A2058 (NPET) reduce affinity of antibiotics to the ribosome.^4^ ABC-F proteins, previously thought to mediate efflux, bind to stalled ribosomes and promote the release of ribosome-targeting antibiotics.^10–12^ Resistance due to binding site modification can be overcome by synergy, as exemplified by streptogramins and recently demonstrated with the combination of hygromycin A and macrolides,^13^ by conformational preorganization of the ligand as recently demonstrated with cresomycin,^9^ or by modification of binding kinetics (reducing off rates), as evidenced by the additional aryl-alkyl side chain in the ketolides.^14^

The hybridization of multiple antibiotic pharmacophores into a single hybrid antibiotic has emerged as a promising strategy to combat resistance,^15,16^ with at least six candidates reaching clinical trials.^17^ Hybrid antibiotics can overcome resistance to either or both of their constituent pharmacophores^18^ and can suppress the development of resistance.^19^ One such strategy involves attaching a ribosome inhibitor to an antibiotic that binds to a completely different target, as recently demonstrated for the macrolones, which contain warheads that target the NPET and type II topoisomerases.^20^ Such hybrids have the advantage that their targets have separate resistance profiles and do not induce resistance, but they can only engage one binding site at a time, negating any potency gains from synergistic binding. A second strategy comprises hybridization of warheads that bind adjacent sites in the same target (such as the PTC and NPET), enabling simultaneous and cooperative binding to both sites. This presents an additional design challenge since the geometry of the linker must facilitate precise positioning of the two inhibitors into their constituent binding sites, and reports of such hybrids are sparse in the literature (see Figure S1 for a short summary of previous hybrid molecules). An early example was published in 1993, wherein Zemlicka and colleagues synthesized hybrids of chloramphenicol, sparsomycin, lincomycin, and puromycin, one of which had moderate inhibitory activity against *Staphylococcus aureus*.^21^ Researchers at Rib-X developed azithromycin-florfenicol hybrid RX-2102 that overcomes resistance by mutation and monomethylation of A2058,^22,23^ oxazolidinone-sparsomycin hybrid RX-01 that overcomes linezolid resistance,^24–27^ and macrolide-linezolid hybrids.^28^ More recently, Andrade and colleagues synthesized a solithromycin-linezolid hybrid that exhibited improved activity against methicillin-resistant *Staphylococcus aureus* (MRSA) compared to the parent molecules.^29^ However, a structure-guided approach that harnesses the recent wealth of ribosome–antibiotic binding data to design potent hybrids has not yet been reported.

Here we describe the design, synthesis, and microbiological and structural characterization of several new hybrid antibiotics that bind to the bacterial ribosome. Using cryo-EM, we determine their binding along the PTC and NPET at high-resolution. We evaluate select candidates against a panel of staphylococci, enterococci, and streptococci with well-characterized, clinically relevant resistance mechanisms. These data provide a platform for the continued optimization of hybrid antibiotics that disrupt the catalytic center of the ribosome.

## Results

### Hybrid Design and Functionalization of Azithromycin

We chose azithromycin (**AZI**, Figure 1a), which is commonly used for the treatment of both Gram-positive and Gram-negative infections and has a good safety profile and optimal pharmacokinetic properties among macrolides, as one hybrid component. **AZI** binds in the NPET with its C5-pendant desosamine sugar angled toward the PTC (Figure 1b,c). For the second part of the hybrid, we focused on PTC binders that are proximal to azithromycin, choosing chloramphenicol (**CHL**), an essential medicine on the WHO list, and tedizolid (**TDZ**), an oxazolidinone antibiotic with potent activity against multidrug-resistant Gram-positive pathogens (Figure 1a). Each of these PTC-binding antibiotics suffers from toxicity related to mitochondrial protein synthesis inhibition. An added potential benefit to generating a hybrid of azithromycin with each of these molecules is that it may mitigate mitochondrial toxicity by reducing accumulation in mitochondria, reducing binding to mitochondrial ribosomes, or both. This has particular relevance to chloramphenicol, which can cause irreversible, fatal aplastic anemia due to its effects on mitochondrial function.^30^

**Figure 1:**
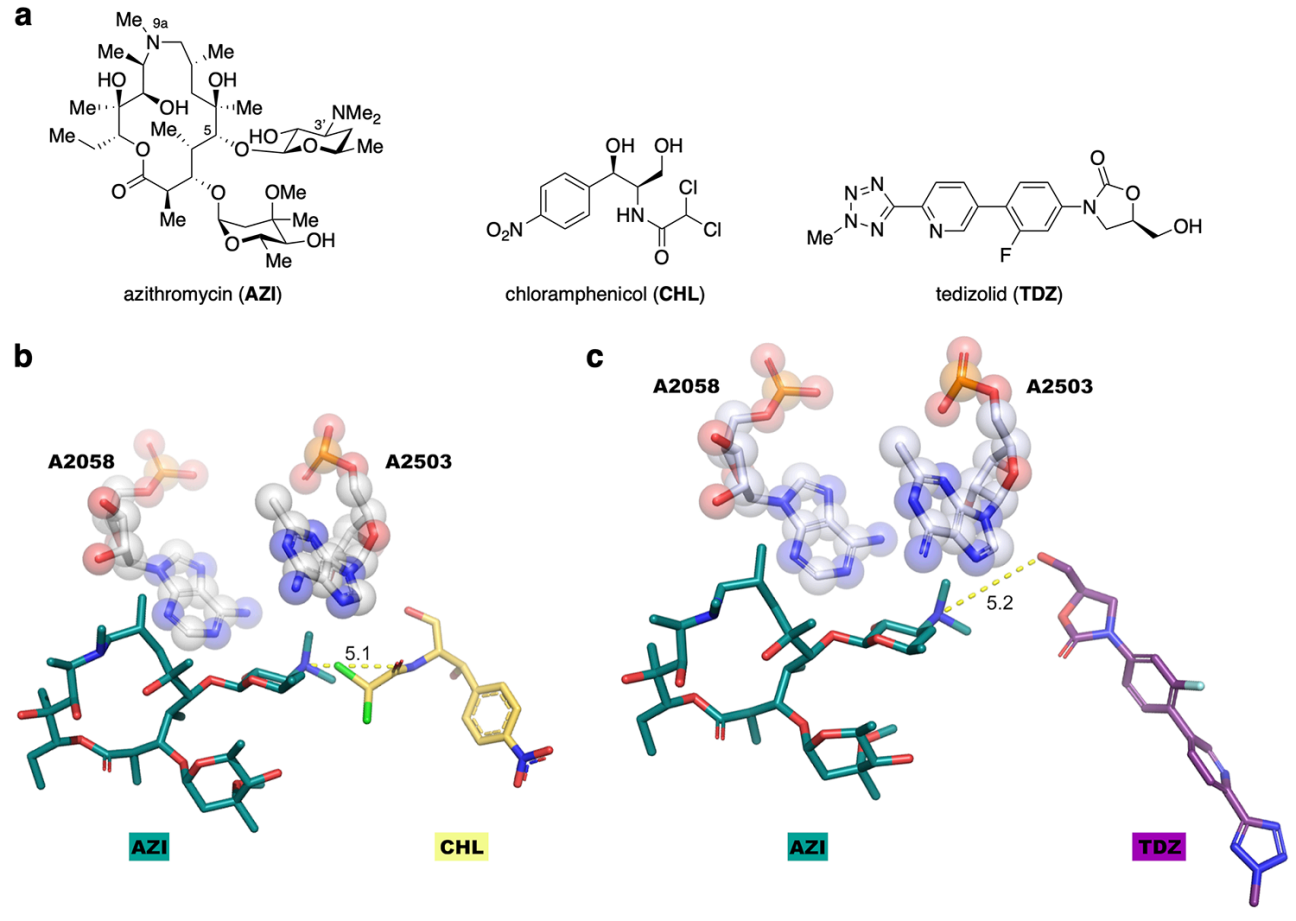
Design of antibiotics that bind to the NPET and PTC. a) Chemical structures of azithromycin (**AZI**), chloramphenicol (**CHL**), and tedizolid (**TDZ**). b) Overlay of **AZI** (PDB: 4V7Y) and **CHL** (PDB: 7RQE) reveals a 5.1-Å distance between the dichloroacetamide nitrogen of **CHL** and the desosamine 3′ nitrogen of **AZI**, with promising linker attachment points. The residues affected by Erm (A2058) and Cfr (A2503) resistance determinants, via base methylation, are shown in spheres. c) As in (b), but **TDZ** (PDB: 6WRS) in the PTC. This comparison reveals a 5.2-Å distance between the desosamine 3′ nitrogen in **AZI** and the primary alcohol of **TDZ**.

The overlay of **AZI**- and **CHL**-bound *Thermus thermophilus* ribosomes (PDBs: 4V7Y and 7RQE, respectively) indicated that one of the chlorine atoms in the dichloroacetamide of **CHL** would clash with the C3′ desosamine dimethylamine in **AZI** (Figure 1b), in agreement with previous data for the macrolide erythromycin and **CHL**.^31^ This suggested that replacement of the dichloroacetamide with a nonbranched linker emanating from the nitrogen of chloramphenicol 5.1 Å from the desosamine nitrogen might lead to a functional hybrid, similar to RX-2102.^22^ Based on an overlay of the structure of **AZI** bound to *Escherichia coli* ribosomes (PDB: 8FC2) and **TDZ** bound to *S. aureus* ribosomes (PDB: 6WRU), we found that the tedizolid C5 alcohol was 5.2 Å from the desosamine C3′ nitrogen (Figure 1c), suggesting a short linker would result in a hybrid compatible with binding.^13^ We next aimed to semisynthetically modify each of these sites in the parent molecules to enable their hybridization.

To enable attachment to **AZI**, we monodemethylated the desosamine 3′ dimethylamine, providing a monomethylamine that could be used directly for hybridization or decorated with a linker. Unlike other macrolides such as erythromycin and solithromycin, **AZI**’s additional amine at position 9a within the macrocycle posed a potential selectivity challenge (it could be demethylated in competition with the 3′ dimethylamine). We found that conditions previously published by Andrade and co-workers for ketolides, which employed iodine and sodium acetate in refluxing methanol/water, led to low yields on **AZI**.^29^ However, we found that the inclusion of Tris buffer and a lower temperature (50 °C) led to reproducibly good yields of hybrid precursor **1** (73%, Figure 2). To access click analogs, we also propargylated the secondary amine in **1** to reach the alkyne hybrid precursor **2**.

**Figure 2:**
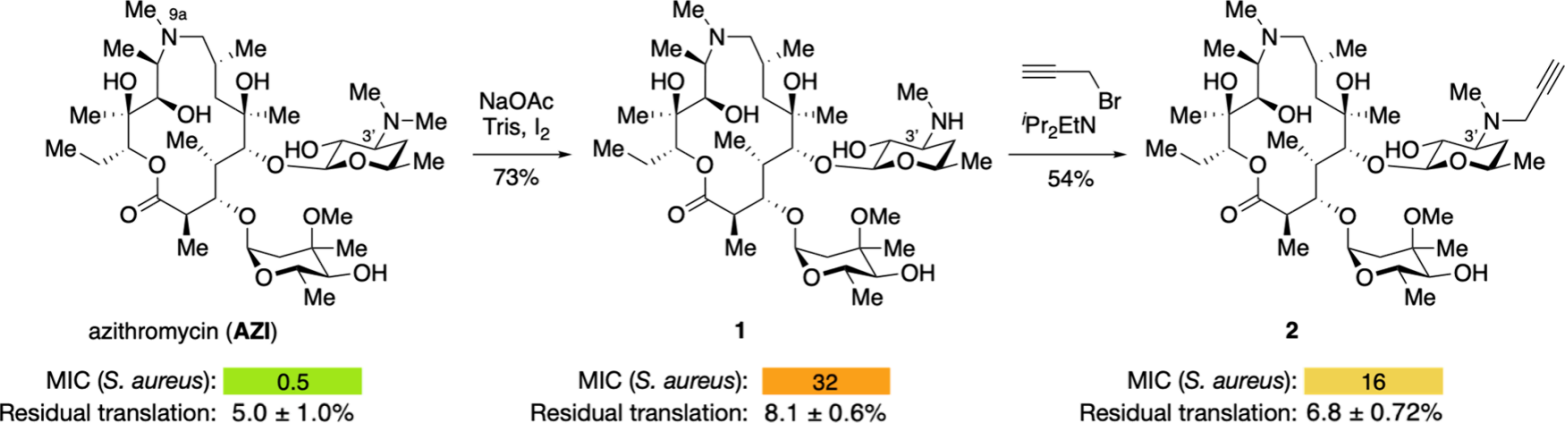
Desosamine-modified azithromycin derivatives for hybridization. Minimum inhibitory concentrations (MICs) were measured against *S. aureus* Newman by broth microdilution and are given in μg/mL. Residual translation compared to vehicle control in an in vitro transcription-coupled translation assay using GFP DNA.

We measured the ability of **AZI**, **1**, and **2** to inhibit the growth of *S. aureus*, a representative Gram-positive organism with a high clinical burden.^3^ Hybrid precursors **1** and **2** exhibited reduced inhibitory activity (32 μg/mL and 16 μg/mL, respectively) compared to azithromycin (0.5 μg/mL). Modifications to antibiotic scaffolds can have profound effects on their ability to accumulate in bacterial cells.^32,33^ Thus, compounds that may strongly inhibit protein synthesis but cannot reach their targets in bacteria due to poor accumulation may appear as inactive in an MIC assay. To decouple cellular activity with protein synthesis inhibitory activity, we measured the ability of each molecule to inhibit the production of GFP in a cell-free transcription-coupled in vitro translation (IVT) assay. We found that **AZI**, **1**, and **2** all inhibited translation in vitro at 10 μM (5%, 8.1%, and 6.8% residual translation, respectively), suggesting that the major contributor for the reduced activity of **1** and **2** was not disruption of their binding to the ribosome. The elevated MIC for **1** might be explained by the reduced shielding of the C3′ desmethyl amine, which is likely to be protonated and will hinder passive diffusion across the lipid membrane. The reduced cellular activity of **2**, however, is more challenging to explain as the C3′ amine is more shielded; reduced activity for this compound may arise from another mechanism besides hindered accumulation. It is important to note that the cellular and in vitro inhibitory activity of these components may not correlate with hybrids derived from them.

### Synthesis and Evaluation of AZI-CHL Hybrids

To access **AZI**-**CHL** hybrids, we subjected **CHL** to acidic hydrolysis followed by silylation of the alcohols to provide amine **3** (Figure 3a). Acylation with chloroacetyl chloride delivered chloroacetamide **4**, which was coupled to desmethylazithromycin (**1**) in the presence of triethylamine and sodium iodide. Treatment with tetrabutylammonium fluoride provided **6**, an **AZI**-**CHL** hybrid with an acetamide linker. We also converted **4** into azide **5** by means of sodium azide. Copper-catalyzed azide–alkyne cycloaddition with alkyne **2** followed by desilylation gave access to triazole-linked **AZI**-**CHL** hybrid **7**.

**Figure 3:**
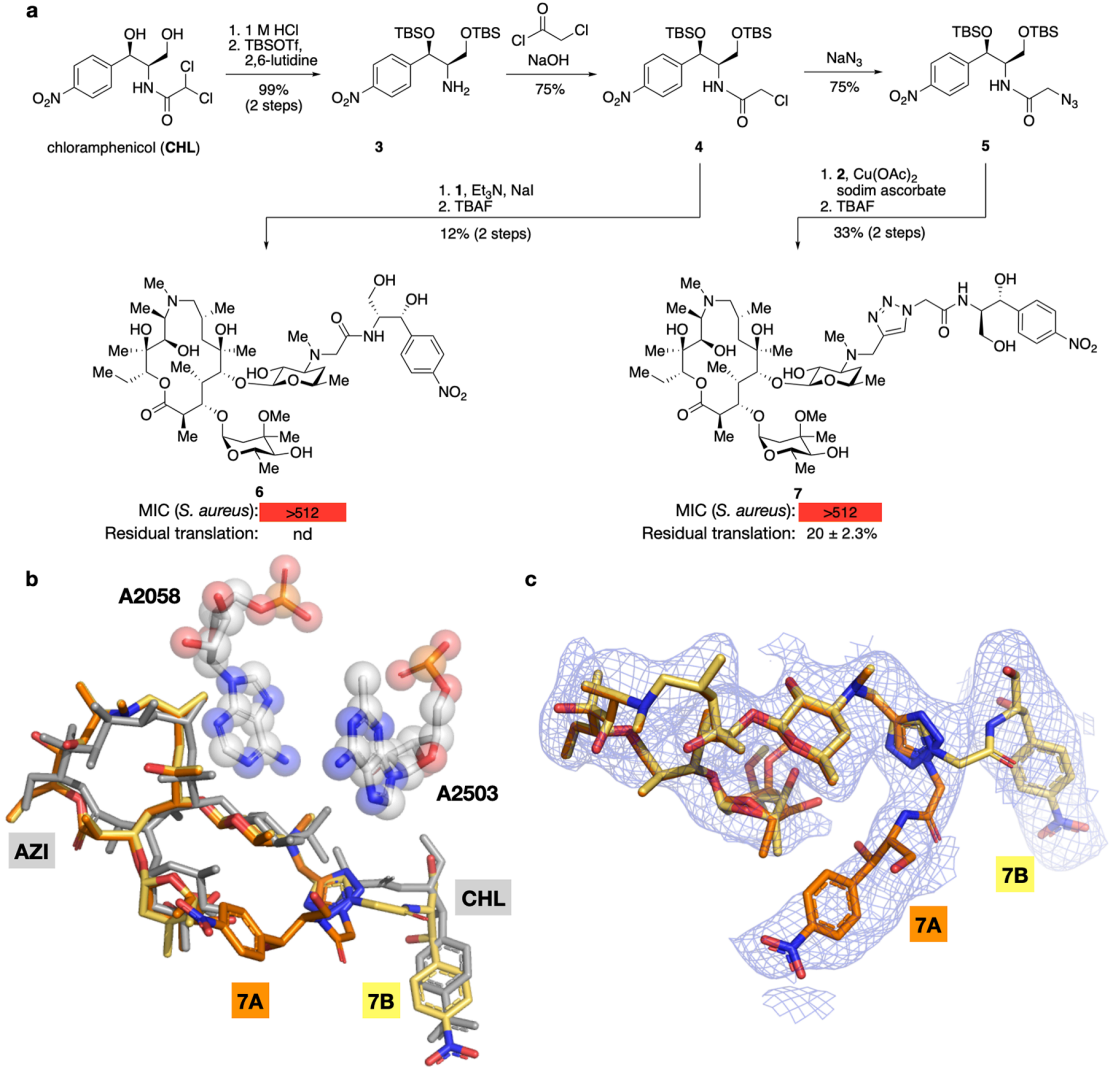
Synthesis and evaluation of **AZI**-**CHL** hybrids. a) Synthesis, MICs, and in vitro translation inhibition of hybrids **6** and **7**. MICs were measured against *S. aureus* Newman by broth microdilution and are given in μg/mL. Residual fluorescence after 1 h in the presence of 10 μM compound compared to vehicle control in an in vitro transcription-coupled translation assay using GFP DNA. b) Overlay of **7** (orange/yellow) (PDB: 8E46) with the individual structures of **AZI** (PDB: 4V7Y) and **CHL** (PDB: 7RQE) (both in gray). The bases methylated by Erm (A2058) and Cfr (A2503) resistance enzymes are shown as spheres. The overlay shows multiple conformations for the chloramphenicol part of the hybrid molecule. Conformation “7B” closely resembles the normal binding pose of **CHL** whereas conformation “7A” extends toward the E site of the PTC. In contrast, the binding pose of the **AZI** component is similar to expectation. c) A zoomed-in view, rotated ∼55° relative to (b) for clarity. The EM potential density (2.3-Å resolution, shown at a normalized σ of 1.0) reveals strong support for multiple conformations of the **CHL** portion, but a disordered or strained linker for the “B” conformation. nd, not determined.

Hybrids **6** and **7** failed to inhibit the growth of *S. aureus* Newman, but triazole-linked **7** showed moderate inhibition of translation in vitro (20% residual translation at 10 μM). We determined a 2.3-Å cryo-EM structure of **7** bound to the *E. coli* ribosome, revealing two binding conformations for the **CHL** portion of the hybrid (Figure 3b,c). The minor conformation (7B, yellow) is closest to the normal binding pose for **CHL**, but it is displaced relative to other **CHL** structures. The dominant conformation (7A, orange) adopts a dramatically different binding pose, rotating nearly 90 deg and extending through the PTC to engage U2586. While both poses are well supported by the density of the 2.3-Å resolution map, the linker density is stronger for the perturbed conformation. Taken together, these data indicate that hybrid **7**, despite a lack of antibiotic activity, is capable of binding to and inhibiting the ribosome, albeit with inefficient placement of the **CHL** portion of the hybrid into the cognate **CHL** binding site.

### Synthesis and Evaluation of AZI-TDZ Hybrids

We next turned our efforts to the synthesis of **AZI**-**TDZ** hybrids, using the primary alcohol in **TDZ** as a functional handle to install a variety of linkers. We treated **TDZ** with methanesulfonyl chloride and triethylamine followed by sodium azide to provide azide **8** in 89% yield (Figure 4). Copper-catalyzed azide–alkyne cycloaddition with alkyne **2** provided hybrid **11** in 66% yield. To access linkers that contain a hydrogen bond donor at C5, which has been shown to play an important role in the binding of many oxazolidinones,^34^ we subjected azide **8** to Staudinger reduction conditions (triphenylphosphine) followed by acylation of the intermediate primary amine with chloroacetyl chloride, delivering chloroacetamide **9** in 83% yield. Coupling with **1** provided acetamide-linked hybrid **12**. Finally, we extended the linker length by treatment of chloroacetamide **9** with 2-mercaptoethanol followed by methanesulfonylation of the resulting primary alcohol, providing methanesulfonate **10**. Coupling of **10** with **1** provided extended-linker **AZI**-**TDZ** hybrid **13**.

**Figure 4:**
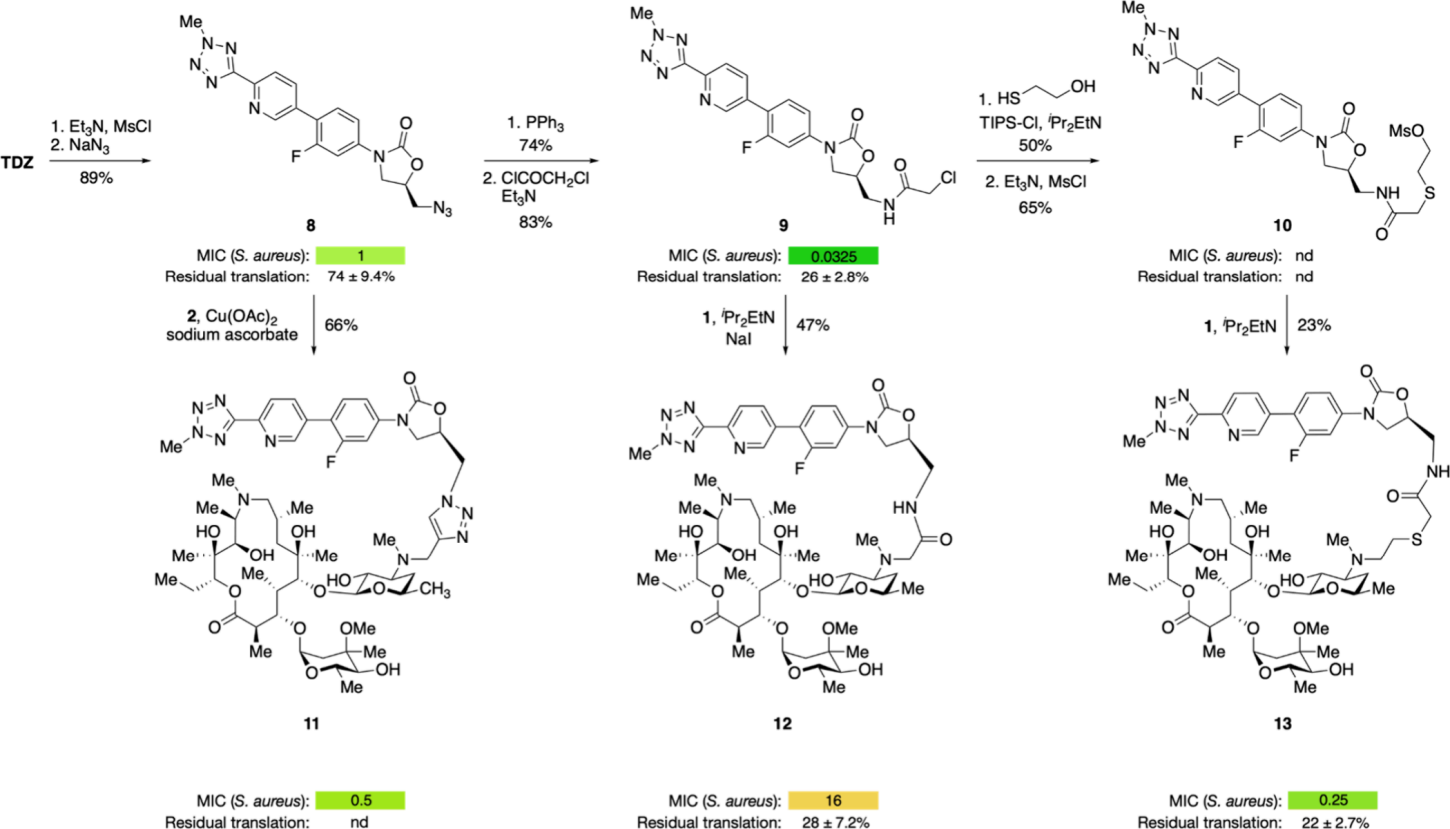
**AZI**-**TDZ** hybrid synthesis and inhibitory activity. MICs were measured against *S. aureus* Newman by broth microdilution and are given in μg/mL. Residual fluorescence after 1 h in the presence of 10 μM compound compared to vehicle control in an in vitro transcription-coupled translation assay using GFP DNA. nd, not determined.

We evaluated hybrids **11**–**13** and intermediates **8** and **9** for their ability to inhibit the growth of *S. aureus* and to inhibit translation in vitro. Intermediate **9**, a chloroacetamide derivative of tedizolid, completely inhibited *S. aureus* growth at 0.03 μg/mL (73 nM); however, it only partially inhibited translation at 10 μM in vitro (26 ± 2.8% residual translation). This suggests that **9** may have additional mechanisms of action beyond translation inhibition or that its action on translation in a longer (20+ hour) cell growth experiment may differ from its action in a 1-h in vitro reconstituted translation experiment. This discrepancy might result from covalent engagement of the ribosome by **9** through attack on the chloroacetamide by A2062, forming a covalent adduct. Prolonged residence time on ribosomes (e.g., slow off rates) have been associated with increased bacterial killing.^14^ We determined a cryo-EM structure of **9** bound to the *E. coli* ribosome, which unambiguously showed that the chloroacetamide functional group was still intact (Figure 5), supporting a reversible binding mode to the ribosome. The enhanced cellular activity of **9** may arise from increased accumulation or from off-target effects on *S. aureus* proteins owing to its reactive chloroacetamide. This hypothesis is supported by recent data from the Hacker group that measured engagement of 230 cysteines in the *S. aureus* proteome with a promiscuous chloroacetamide probe,^35^ and likely tampers any therapeutic potential of this molecule.

**Figure 5:**
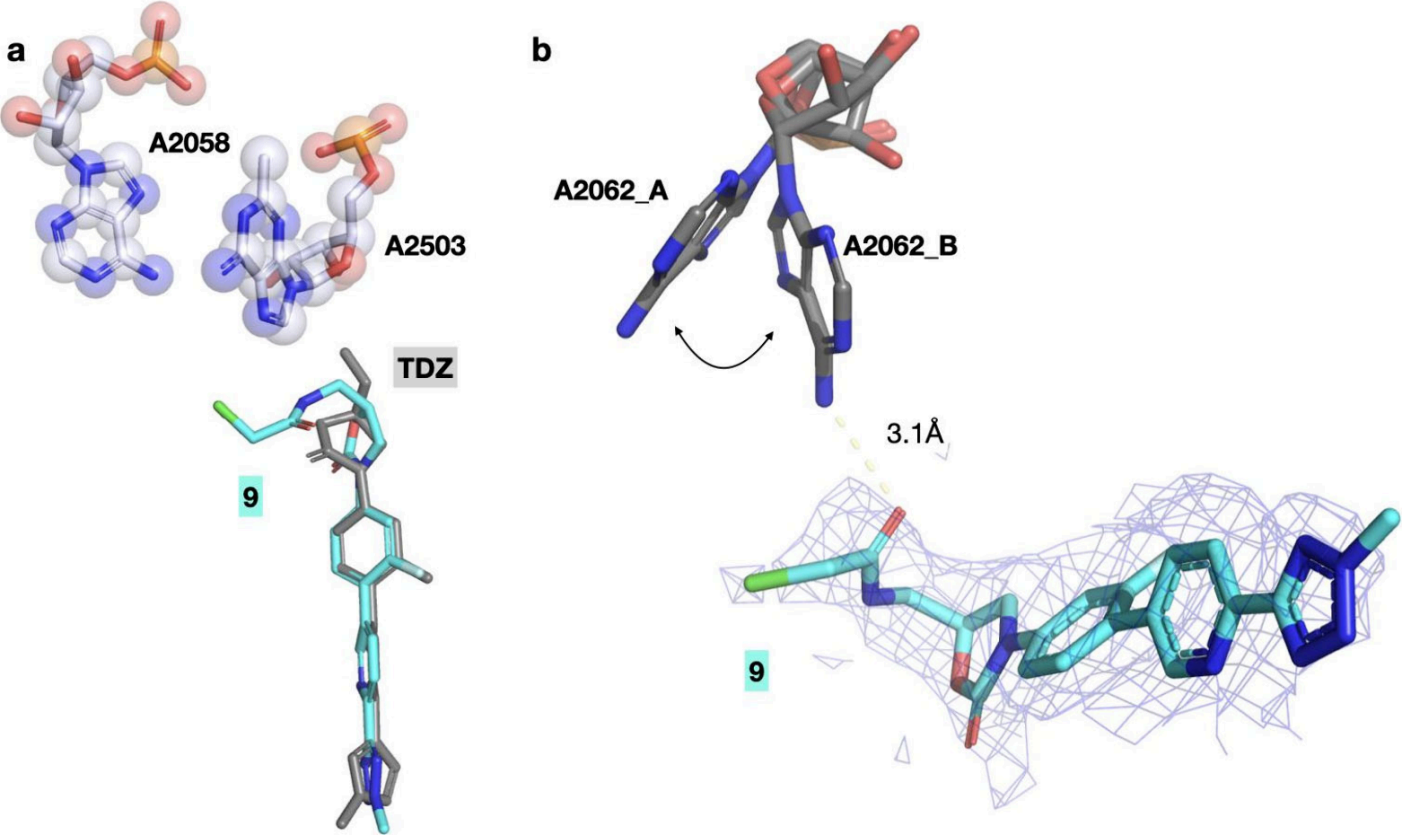
Compound **9** is a reversible ribosome inhibitor. a) Overlay of **9** (cyan) with the individual structure **TDZ** (gray, PDB: 6WRS). The binding pose of the **TDZ** component is similar to expectation with the chloroacetamide extending toward the NPET. b) A zoomed-in view showing the EM potential density (2.5-Å resolution, shown at a normalized σ of 1.0) does not reveal any continuous density for a reacted chloroacetamide. The potential interaction of the carbonyl with one of the alternative conformations of A2062 is shown, but likely not relevant as no stabilization of that base to this conformation is observed.

Hybrids **11** and **13** potently inhibit the growth of *S. aureus* (MICs = 0.5 and 0.25 μg/mL, respectively). Hybrid **12**, which has the shortest linker of the three, exhibited moderate inhibitory activity (MIC = 16 μg/mL). To investigate the consequences of the varied linkers on binding, we determined cryo-EM structures of **11**, **12**, and **13** in complex with the *E. coli* ribosome (Figure 6a). We found that hybrid **11** shows a density for the linker region (orange arrow, Figure 6b). However, when aligned with reference to the **AZI** macrocycle, the **TDZ** portion of the hybrid was displaced relative to the native binding site of **TDZ**, potentially due to the rigid triazole linker (Figure 6a). The cryo-EM map for hybrid **12** had electron potential density for correct positioning of the **TDZ** portion of the hybrid but also contained a second portion of density (red arrow, Figure 6c). This density may represent a second binding pose in which the **TDZ** portion is mispositioned, likely as a consequence of the short linker; however, unlike compound **7**, the weak density did not allow confident modeling of a second conformation. In the cryo-EM structure for **13**, strong electron potential density is present only for one conformation that correctly positions the **TDZ** portion (Figure 6d**)**. Weak density around the linker may arise due to conformational flexibility (green arrow, Figure 6d), which may aid in correct positioning.

**Figure 6:**
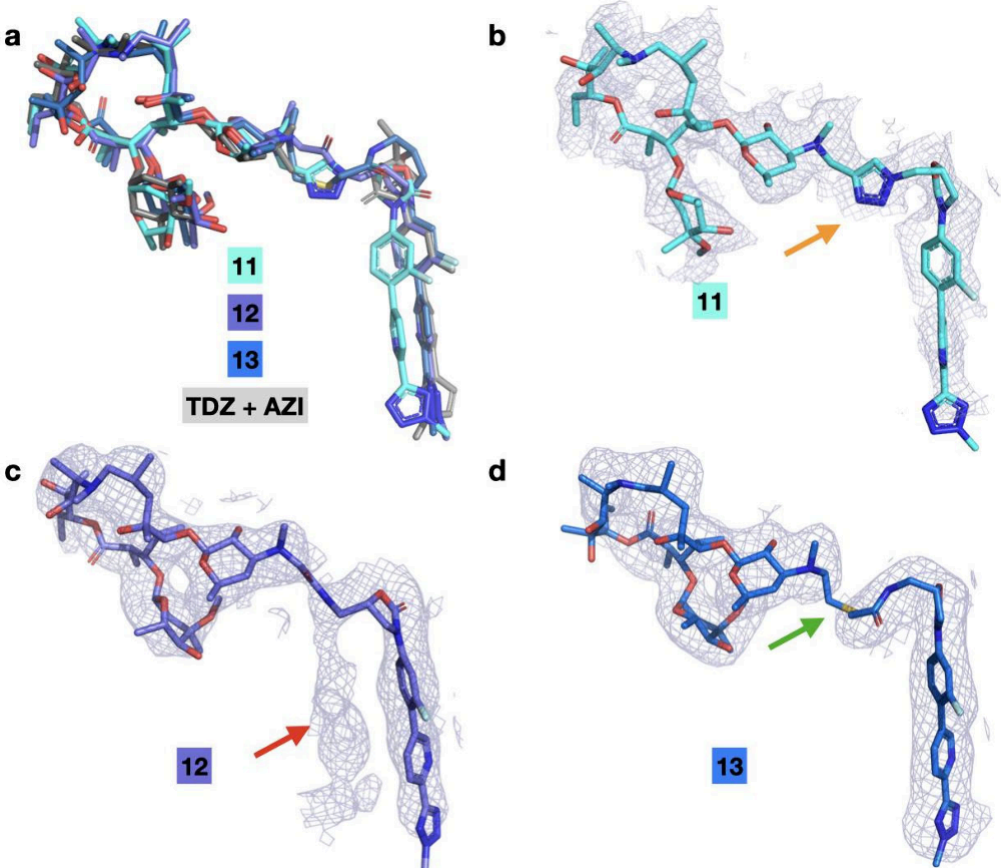
Linkers in **AZI**-**TDZ** determine the placement of the **TDZ** component. a) Overlay of the cryo-EM structures of **11** (cyan), **12** (purple), **13** (blue), and **AZI** and **TDZ** (gray, PDBs: 4V7Y and 6WRS). b) Density for **11** (2.4 Å, 1.3 σ, cyan, PDB: 9EBD), including the rigid triazole linker (orange arrow). c) Density for **12** (PDB: 8E45) including the second portion of the density possibly indicating an alternate pose (red arrow). d) Density for **13** (blue, PDB: 8E47). Weak density for the linker region is indicated by a green arrow.

### MICs in an Expanded Panel of Gram-Positive Pathogens

We tested compounds **11**–**13** in an expanded panel of Gram-positive pathogens, focusing on strains with characterized resistance to macrolides, oxazolidinones, lincosamides, phenicols, and streptogramins (grouped by phenotype in Table 1 for **11** and **13**, selected individual strain data for **11**–**13** in Table S1, including other comparator antibiotics). Erythromycin methylases (Erms) cause (di)­methylation of A2058, disrupting binding of macrolide antibiotics such as **AZI** as well as lincosamides and streptogramins B (the so-called MLS_B_ phenotype).^36^ Erm resistance can be induced in the presence of certain macrolides (including **AZI**) or can be constitutively activated. Analogously, methylation of A2503 by Cfr proteins can disrupt binding of PTC-binding antibiotics such as **TDZ**.^34^ Mutations in ribosomal proteins L4, L22, or at positions 2058/2059 can cause resistance to **AZI** and, in some cases, to oxazolidinones.^37^ Similarly, G2576T mutations can cause oxazolidinone resistance, although the level of resistance is often proportional to the number of mutated 23S rRNA alleles.^38^ Finally, ribosome-protecting ABC-F proteins such as Msr­(A), Vga­(A), OptrA, and PoxtA cause resistance by intercepting antibiotic-stalled ribosomes and restoring protein synthesis.^10^

**Table 1:**
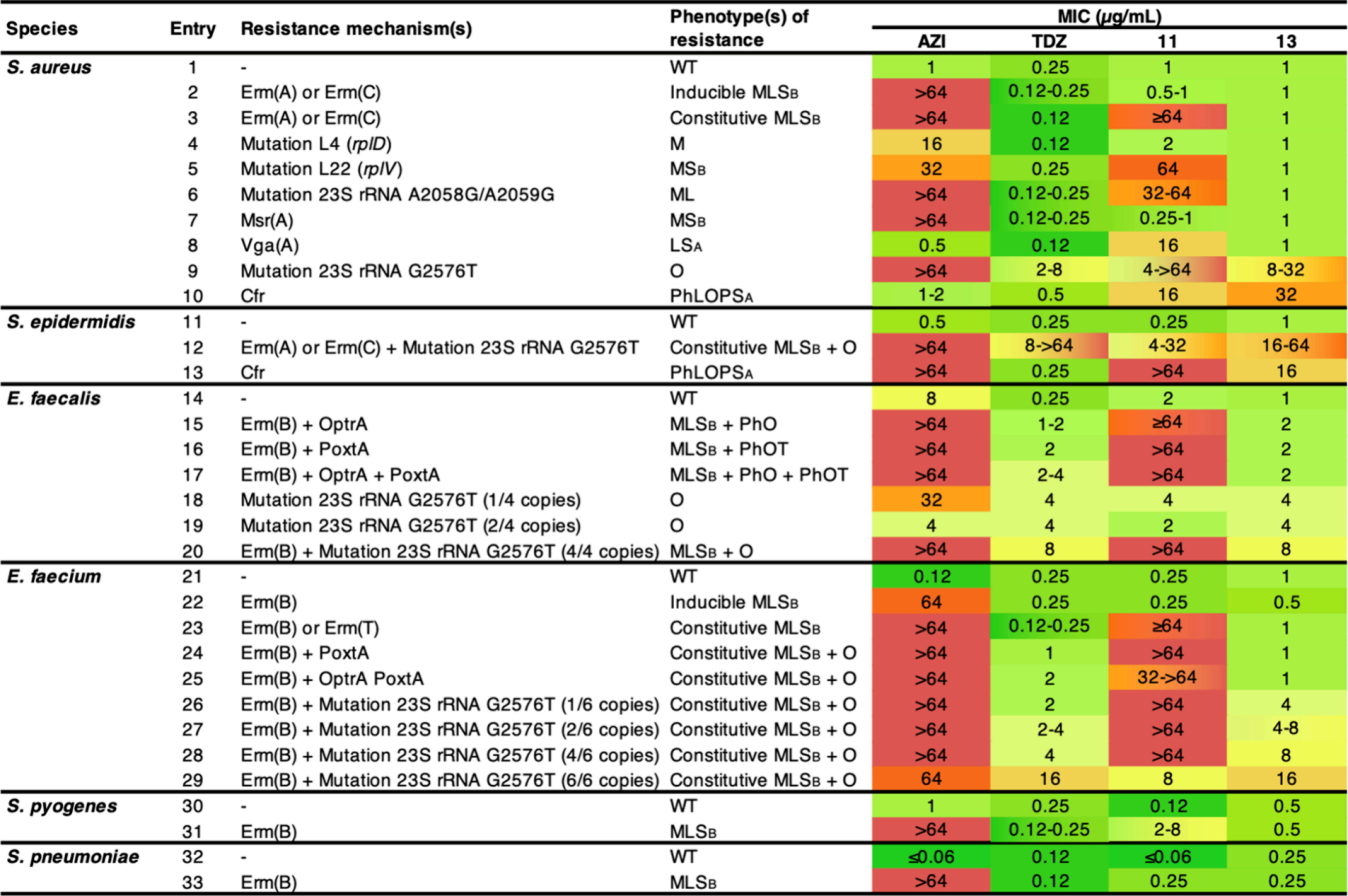
Inhibitory Activity against Bacteria with Macrolide and Oxazolidinone Resistance^a^

a Strains are grouped by phenotype. For data against select individual strains with additional marketed antibiotics, see Table S1. Abbreviations: LS_A_, lincosamides-streptogramins A; ML, macrolides-lincosamides; MLS_B_, macrolides-lincosamides-streptogramins B; MS_B_, macrolides-streptogramins B; O, oxazolidinones; PhLOPS_A_, phenicols-lincosamides-oxazolidinones-pleuromutilins-streptogramins A; PhO, phenicols-oxazolidinones; PhOT, phenicols-oxazolidinones-tetracyclines; WT, wild-type.

Resistance to **AZI** is reflected in high MIC values in all strains that harbor macrolide resistance mechanisms (Table 1). Comparatively, hybrid **11** maintains activity against strains with inducible Erm resistance (entries 2 and 22) but is not active when Erm proteins are constitutively expressed (e.g., entries 3, 12) except against a multidrug-resistant strain of *E. faecium* (entry 29). Hybrid **11** also maintains activity against staphylococci and enterococci with mutations in ribosomal protein L4, mutations in 23S rRNA (A2058G/A2059G), or harboring the macrolide ABC-F protection protein Msr­(A). It is inactive against bacteria with modifications to the PTC such as Cfr methylation of A2503 (entries 10, 13) or ABC-F proteins that affect PTC-binding antibiotics such as Vga­(A) (entry 8). Hybrid **12** was inactive against most strains (Table S1).

Hybrid **13** is active against all strains that harbor Erm-mediated resistance, whether inducible or constitutive. It also maintains activity against strains with mutations in L4 and L22 ribosomal proteins and mutations in 23S rRNA (A2058G/A2059G), all of which typically confer macrolide resistance. It is minimally affected by ABC-F ribosomal protection resistance mediated by Msr­(A), Vga­(A), PoxtA, or OptrA. In most strains with oxazolidinone resistance-associated G2576T mutations, hybrid **13** shows comparable activity to **TDZ**, but methylation of A2503 by Cfr proteins in staphylococci confers resistance to **13** but not **TDZ**.

### Effects on Mammalian Protein Synthesis

We measured the effects of **CHL**, **AZI**, **TDZ**, **11**, and **13** on mammalian protein synthesis and cell viability using quantitative ELISA to measure the production of cyclooxygenase-1 (COX-1, synthesized by the mitoribosome) and succinate dehydrogenase complex flavoprotein subunit A (SDH-A, synthesized by the cytosolic ribosome) in PC3 cells (see Figure S2). **AZI** exhibits minimal effects on mammalian protein synthesis and cell viability at concentrations as high as 50 μM. **CHL** and **TDZ** inhibit mitochondrial protein synthesis (>50% reduction in COX-1 levels at 3 μM **TDZ**) but have minimal effects on cell viability and cytosolic protein synthesis.^39^ Hybrid **11** showed a reduced ability to inhibit mitochondrial protein synthesis (25 μM required for a ∼ 50% reduction in COX-1 expression) compared to **TDZ**. Hybrid **13** inhibited expression of COX-1 in a manner similar to **TDZ** and also showed marked effects on cytosolic protein synthesis and cell viability at higher concentrations (≥25 μM).

## Discussion

The design of active hybrid antibiotics is inherently challenging. Accumulation in bacterial cells can be hard to predict and assess,^40,41^ complicating hybrid design. Differences in size and exposed polar surface area of the hybrids compared to their individual components can have profound effects on cellular accumulation, and thus activity in MIC assays. **AZI-CHL** hybrid **7**, for example, is capable of inhibiting translation in vitro but does not inhibit the growth of *S. aureus*, even at 512 μg/mL.

Additionally, for hybrids that bind to the same target, a suitable linker must be selected to enable the precise placement of the hybrid components into their constituent binding sites. Suboptimal linker design can result in the displacement of the components from their binding poses or in the adoption of multiple conformations of the hybrid molecules. For all hybrids whose binding was characterized by cryo-EM, the **AZI** macrocycle is correctly placed in the NPET, indicating it plays an important role in hybrid positioning. This hypothesis is further supported by lack of activity and displaced sugar positioning in oxazolidinone–desosamine hybrids, which lack the **AZI** macrocycle (Figure S3). We observe displacement of individual components for hybrids **7**, **9**, and **11**. The precise placement of these components is especially important because both **CHL** and oxazolidinone antibiotics (like **TDZ**) form contacts with the nascent peptide chain.^39,42,43^ We also observe multiple conformations for hybrids **7** and **12**, which could present an opportunity to create new molecules that adopt characteristics of both conformations.^44,45^ However, in this case, it appears that the hybrid molecules do not engage in all the productive binding interactions observed in the independent components, leading to reduced activity in cells and in cell-free translation assays.

Prediction and interpretation of hybrid activity is further complicated by the presence of resistance mechanisms. The resistance patterns of the parent classes of antibiotics may or may not be reflected in resistance to a given antibiotic hybrid. Hybrids can overcome resistance of one or both components (or neither), and these patterns can be strain-specific. Hybrid **11**, for example, overcomes some macrolide resistance mechanisms but is still susceptible to oxazolidinone resistance mechanisms. Its ability to overcome inducible Erm resistance may arise from differences in its ability to selectively stall the *erm* leader peptide,^46^ but its lack of activity in constitutive Erm strains suggests it cannot inhibit A2058-methylated ribosomes.^47^ Its activity in strains with macrolide resistance mechanisms such as Msr­(A) (ribosomal protection), L4 mutations, and A2058G mutations suggests that its oxazolidinone portion is playing a functional role in ribosomal inhibition.

Hybrid **13** maintains activity against almost all strains tested, including those with constitutive resistance to macrolides and oxazolidinones. In most strains, it inhibits growth with MICs of 2- to 8-fold that of **TDZ**. Compared to the most commonly prescribed oxazolidinone antibiotic linezolid,^38^ **13** is equally active or more active in all strains tested (Table S1). Against strains harboring ABC-F resistance to phenicols and oxazolidinones (PoxtA and OptrA), **13** shows comparable activity to **TDZ**, and in some cases slightly improved activity (entries 17 and 25). Hybrid **13** is affected less by G2576T resistance than **TDZ**, with a maximum of 8- to 16-fold increase in MIC for **13** versus 32- to 64-fold for **TDZ** (entries 20 and 29). Contrastingly, hybrid **13** is highly susceptible to Cfr-mediated resistance in staphylococci, which has a minimal effect on the activity of **TDZ** (entries 10 and 13). This could arise from the macrolide portion of **13** preventing the tedizolid portion from positionally adapting in the presence of C8-methylated A2503, resulting in decreased binding of the hybrid.

The activity of hybrid **13** against strains with ABC-F-mediated phenicol-oxazolidinone (PhO) resistance mechanisms merits further discussion. Phenicols (such as **CHL**) and oxazolidinones (such as **TDZ**) are context-specific translation inhibitors that preferentially bind to ribosomes occupied by P-site tRNA and a nascent chain that contains alanine (and to a lesser extent, serine or threonine) at the penultimate (−1) position.^42,43^ The ABC-F proteins PoxtA and OptrA bind to stalled ribosomes in the E-site, and their Antibiotic Resistance Domain (ARD) extends toward the catalytic center and interacts with P-site tRNA, which bears the nascent polypeptide.^48^ Upon successful binding, it is hypothesized that the ABC-F protein induces a structural change that leads to antibiotic dissociation, possibly by perturbing the positioning of the nascent chain and disrupting its (context-specific) interaction with the drug. This results in resistance to phenicols and oxazolidinones, which we observe with **TDZ** in both *E. faecalis* (4- to 16-fold increase of MIC compared to WT, entries 15–17) and in *E. faecium* (4- to 8-fold increase in MIC, entries 24–25). Unlike **TDZ** and linezolid (Table S1), hybrid **13** is minimally affected by these ABC-F resistance mechanisms (2-fold increase in MIC in *E. faecalis* and no change in MIC in *E. faecium*). An overlay of our ribosome-bound structure of **13** with the PoxtA/P-site tRNA-bound structure (Figure S4) reveals that **13** does not directly clash with the PoxtA ARD or with P-site tRNA, but it does occupy the space where the nascent chain would reside. Possible structural explanations of this lack of susceptibility to ABC-F resistance include 1) inability of the ARD to effectively position itself in the presence of **13**, 2) the position of P-site tRNA and the nascent chain in the presence of **13** precludes ABC-F binding, or 3) hybrid **13** acts as an initiation inhibitor, and the inhibited initiation complex is not a substrate for PoxtA or OptrA. Future work will focus on microbiological and structural studies to differentiate between these putative molecular mechanisms and on structural modifications to oxazolidinones to achieve the same result without attachment of an entire macrolide antibiotic.

The reduced inhibition of mitochondrial protein synthesis by **11** compared to **TDZ** showcases the potential of hybrid antibiotics to mitigate mitochondrial toxicities of parent classes, which is a longstanding challenge among oxazolidinone antibiotics.^39^ It is unclear why hybrid **11** exhibits reduced mitochondrial toxicity compared to **TDZ**, while hybrid **13** does not. Synthesis of more hybrid molecules like **11** and **13**, with varied linkers and antibiotic components, would reveal structure–toxicity relationships that might lead to a mechanistic understanding of the determinants of mitochondrial vs bacterial protein synthesis inhibition.

## Conclusion

In summary, we have synthesized a collection of hybrid antibiotics based on azithromycin, chloramphenicol, and tedizolid designed to inhibit the bacterial ribosome. We evaluated the ability of these hybrids to inhibit the growth of *S. aureus,* identifying two highly active candidates. We used cryo-electron microscopy to characterize the binding of several hybrids and hybrid fragments and found that effective positioning of the two parent pharmacophores was necessary, but not sufficient, for antimicrobial activity. We evaluated the inhibitory activity of select hybrids against a broad panel of Gram-positive pathogens with clinically relevant resistance mechanisms, revealing that hybrid **13** inhibits the growth of nearly all strains and has a resistance profile that differs from its parent antibiotic components. Finally, we evaluated the effects of hybrids **11** and **13** on protein synthesis in human cells, demonstrating that hybrids can modulate both cytosolic and mitochondrial protein synthesis in a manner distinct from the parent classes. This work provides a framework for the design, synthesis, and evaluation of hybridized molecules that bind to the ribosome. More broadly, this work grants insight into the preparation of beyond-rule-of-5 hybrid inhibitors that bind to adjacent binding sites with short and flexibility-limiting linkers, providing guidelines that can be applied generally to targets beyond the ribosome.

## Supplementary Material









## Data Availability

All data are available in the main text, the documents, and the EMDB and PDB databases.

## Notes

Detailed MIC data for compounds **11**, **12**, **13**, and additional comparators in individual, genetically characterized strains (Table S1), examples of previously described hybrid antibiotics (Figure S1), mitochondrial biogenesis data (Figure S2), desosamine hybrids lacking the macrocycle (Figure S3), overlay of compound **13** in the ribosome with PoxtA ABC-F resistance protein (Figure S4), general experimental procedures for chemical synthesis, in vitro translation, MIC determination, mitochondrial biogenesis, and cryo-EM data collection and processing, detailed synthesis procedures and characterization data for new chemical entities, images of NMR spectra for new chemical entities (PDF)
Table containing data collection, processing, and model refinement statistics for cryo-EM experiments (XLSX)
Unprocessed NMR data as fid and jdf files (ZIP)
Transparent Peer Review report available (PDF)

## Notes

Accession codes Models and maps generated during this study are available in the EMDB and PDB with the following accession codes: Compound **7**: 8E46 (EMD-27881), Compound **9**: 8E44 (EMD-27879), Compound **11**: 9EBD (EMD-47878), Compound **12**: 8E45 (EMD-27880), Compound **13**: 8E47 (EMD-27882), Compound **SLC30**: 8E48 (EMD-27883), Compound **SLC31**: 8E49 (EMD-27884).

## References

[1] Antimicrobial
Resistance Collaborators (2022). Global Burden of Bacterial Antimicrobial Resistance in 2019: A Systematic
Analysis. The Lancet.

[2] 2023 Antibacterial agents in clinical and preclinical development: an overview and analysis https://www.who.int/publications/i/item/9789240094000 (accessed Jul 16, 2024).

[3] World Health Organization . WHO Bacterial Priority Pathogens List, 2024: Bacterial Pathogens of Public Health Importance, to Guide Research, Development, and Strategies to Prevent and Control Antimicrobial Resistance; World Health Organization, 2024.

[4] Wilson D. N. (2014). Ribosome-Targeting
Antibiotics and Mechanisms of Bacterial Resistance. Nat. Rev. Microbiol..

[5] Paternoga H., Crowe-McAuliffe C., Bock L. V., Koller T. O., Morici M., Beckert B., Myasnikov A. G., Grubmüller H., Nováček J., Wilson D. N. (2023). Structural Conservation
of Antibiotic Interaction with Ribosomes. Nat.
Struct. Mol. Biol..

[6] Seiple I. B., Zhang Z., Jakubec P., Langlois-Mercier A., Wright P. M., Hog D. T., Yabu K., Allu S. R., Fukuzaki T., Carlsen P. N. (2016). A Platform
for the Discovery
of New Macrolide Antibiotics. Nature.

[7] Li Q., Pellegrino J., Lee D. J., Tran A. A., Chaires H. A., Wang R., Park J. E., Ji K., Chow D., Zhang N. (2020). Synthetic Group A Streptogramin Antibiotics That Overcome
Vat Resistance. Nature.

[8] Mitcheltree M. J., Pisipati A., Syroegin E. A., Silvestre K. J., Klepacki D., Mason J. D., Terwilliger D. W., Testolin G., Pote A. R., Wu K. J. Y. (2021). A Synthetic Antibiotic Class Overcoming Bacterial Multidrug Resistance. Nature.

[9] Wu K. J. Y., Tresco B. I. C., Ramkissoon A., Aleksandrova E. V., Syroegin E. A., See D. N. Y., Liow P., Dittemore G. A., Yu M., Testolin G. (2024). An Antibiotic
Preorganized for Ribosomal
Binding Overcomes Antimicrobial Resistance. Science.

[10] Sharkey L. K. R., O’Neill A. J. (2018). Antibiotic Resistance ABC-F Proteins:
Bringing Target Protection into the Limelight. ACS. Infect. Dis..

[11] Fostier C. R., Monlezun L., Ousalem F., Singh S., Hunt J. F., Boël G. (2021). ABC-F Translation Factors: From Antibiotic
Resistance
to Immune Response. FEBS Lett..

[12] Saha C., Saha S., Chakraborty A., Dirisala A., Maity A., Bhowmik P., Sikder K., Chakraborti S., Basu A. (2023). Deciphering the Structural and Functional Properties of ABC-F ATPases. Infect. Dis. Diag. Treat..

[13] Chen C.-W., Leimer N., Syroegin E. A., Dunand C., Bulman Z. P., Lewis K., Polikanov Y. S., Svetlov M. S. (2023). Structural Insights
into the Mechanism of Overcoming Erm-Mediated Resistance by Macrolides
Acting Together with Hygromycin-A. Nat. Commun..

[14] Svetlov M. S., Vázquez-Laslop N., Mankin A. S. (2017). Kinetics of Drug-Ribosome
Interactions Defines the Cidality of Macrolide Antibiotics. Proc. Natl. Acad. Sci. U. S. A..

[15] Domalaon R., Idowu T., Zhanel G. G., Schweizer F. (2018). Antibiotic
Hybrids: The Next Generation of Agents and Adjuvants against Gram-Negative
Pathogens?. Clin. Microbiol. Rev..

[16] Sampath
Kumar H. M., Herrmann L., Tsogoeva S. B. (2020). Structural Hybridization
as a Facile Approach to New Drug Candidates. Bioorg. Med. Chem. Lett..

[17] Koh A. J. J., Thombare V., Hussein M., Rao G. G., Li J., Velkov T. (2023). Bifunctional Antibiotic Hybrids: A Review of Clinical
Candidates. Front. Pharmacol..

[18] Locher H. H., Caspers P., Bruyère T., Schroeder S., Pfaff P., Knezevic A., Keck W., Ritz D. (2014). Investigations
of the Mode of Action and Resistance Development of Cadazolid, a New
Antibiotic for Treatment of *Clostridium Difficile* Infections. Antimicrob. Agents Chemother..

[19] Martin J. K., Sheehan J. P., Bratton B. P., Moore G. M., Mateus A., Li S. H.-J., Kim H., Rabinowitz J. D., Typas A., Savitski M. M. (2020). A Dual-Mechanism Antibiotic
Kills Gram-Negative Bacteria and Avoids Drug Resistance. Cell.

[20] Aleksandrova E. V., Ma C.-X., Klepacki D., Alizadeh F., Vázquez-Laslop N., Liang J.-H., Polikanov Y. S., Mankin A. S. (2024). Macrolones Target
Bacterial Ribosomes and DNA Gyrase and Can Evade Resistance Mechanisms. Nat. Chem. Biol..

[21] Zemlicka J., Fernandez-Moyano M. C., Ariatti M., Zurenko G. E., Grady J. E., Ballesta J. P. (1993). Hybrids
of Antibiotics Inhibiting Protein Synthesis.
Synthesis and Biological Activity. J. Med. Chem..

[22] Bulkley D., Innis C. A., Blaha G., Steitz T. A. (2010). Revisiting the Structures
of Several Antibiotics Bound to the Bacterial Ribosome. Proc. Natl. Acad. Sci. U. S. A..

[23] Hanselmann R., Job G. E., Johnson G., Lou R., Martynow J. G., Reeve M. M. (2010). Synthesis of an Antibacterial Compound
Containing a
1,4-Substituted 1*H*-1,2,3-Triazole: A Scaleable Alternative
to the “Click” Reaction. Org.
Process Res. Dev..

[24] Franceschi F., Duffy E. M. (2006). Structure-Based Drug Design Meets the Ribosome. Biochem. Pharmacol..

[25] Li S., Cheng X., Zhou Y., Xi Z. (2011). Sparsomycin-Linezolid
Conjugates Can Promote Ribosomal Translocation. Chembiochem.

[26] Zhou J., Bhattacharjee A., Chen S., Chen Y., Duffy E., Farmer J., Goldberg J., Hanselmann R., Ippolito J. A., Lou R. (2008). Design at the Atomic
Level: Generation of Novel Hybrid Biaryloxazolidinones as Promising
New Antibiotics. Bioorg. Med. Chem. Lett..

[27] Skripkin E., McConnell T. S., DeVito J., Lawrence L., Ippolito J. A., Duffy E. M., Sutcliffe J., Franceschi F. R. (2008). Chi-01,
a New Family of Oxazolidinones That Overcome Ribosome-Based Linezolid
Resistance. Antimicrob. Agents Chemother..

[28] Kanyo, Z. ; Bhattacharjee, A. ; Chen, S. ; Chen, Y. ; Dalton, J. ; Devito, J. ; Farmer, J. ; Franceschi, F. ; Goldberg, J. ; Hanselmann, R. ; Ippolito, J. ; Johnson, G. ; Lawrence, L. ; Lou, R. ; McConnell, T. ; Orbin, A. ; Oyelere, A. ; Park, M. ; Salvino, J. ; Sherer, E. ; Sutcliffe, J. ; Tang, Y. ; Wang, D. ; Wu, Y. ; Duffy, E. Enhanced Macrolides: Overcoming Resistance by Improving Target Affinity, from 49th Interscience Conference on Antimicrobial Agents and Chemotherapy, San Francisco, CA, September 12-15, 2009, abstract F1-2050.

[29] Daher S. S., Lee M., Jin X., Teijaro C. N., Barnett P. R., Freundlich J. S., Andrade R. B. (2022). Alternative Approaches
Utilizing Click Chemistry to
Develop next-Generation Analogs of Solithromycin. Eur. J. Med. Chem..

[30] Barnhill A. E., Brewer M. T., Carlson S. A. (2012). Adverse Effects of Antimicrobials
via Predictable or Idiosyncratic Inhibition of Host Mitochondrial
Components. Antimicrob. Agents Chemother..

[31] Svetlov M. S., Plessa E., Chen C.-W., Bougas A., Krokidis M. G., Dinos G. P., Polikanov Y. S. (2019). High-Resolution Crystal Structures
of Ribosome-Bound Chloramphenicol and Erythromycin Provide the Ultimate
Basis for Their Competition. RNA.

[32] Payne D. J., Gwynn M. N., Holmes D. J., Pompliano D. L. (2007). Drugs for
Bad Bugs: Confronting the Challenges of Antibacterial Discovery. Nat. Rev. Drug Discovery.

[33] Muñoz K. A., Hergenrother P. J. (2021). Facilitating
Compound Entry as a Means to Discover
Antibiotics for Gram-Negative Bacteria. Acc.
Chem. Res..

[34] Locke J. B., Finn J., Hilgers M., Morales G., Rahawi S., Kedar G. C., Picazo J. J., Im W., Shaw K. J., Stein J. L. (2010). Structure-Activity Relationships
of Diverse Oxazolidinones
for Linezolid-Resistant *Staphylococcus Aureus* Strains
Possessing the Cfr Methyltransferase Gene or Ribosomal Mutations. Antimicrob. Agents Chemother..

[35] Zanon P. R. A., Yu F., Musacchio P., Lewald L., Zollo M., Krauskopf K., Mrdović D., Raunft P., Maher T. E., Cigler M. (2021). Profiling
the Proteome-Wide Selectivity of Diverse
Electrophiles. ChemRxiv.

[36] Weisblum B. (1995). Erythromycin
Resistance by Ribosome Modification. Antimicrob.
Agents Chemother..

[37] Leclercq R. (2002). Mechanisms
of Resistance to Macrolides and Lincosamides: Nature of the Resistance
Elements and Their Clinical Implications. Clin.
Infect. Dis..

[38] Bozdogan B., Appelbaum P. C. (2004). Oxazolidinones: Activity, Mode of Action, and Mechanism
of Resistance. Int. J. Antimicrob. Agents.

[39] Bibel B., Raskar T., Couvillion M., Lee M., Kleinman J. I., Takeuchi-Tomita N., Churchman L. S., Fraser J. S., Fujimori D. G. (2025). Context-Specific
Inhibition of Mitochondrial Ribosomes by Phenicol and Oxazolidinone
Antibiotics. Nucleic Acids Res..

[40] Łapinska U., Voliotis M., Lee K. K., Campey A., Stone M R. L, Tuck B., Phetsang W., Zhang B., Tsaneva-Atanasova K., Blaskovich M. A., Pagliara S. (2022). Fast Bacterial Growth
Reduces Antibiotic Accumulation and Efficacy. eLife.

[41] Richter M. F., Drown B. S., Riley A. P., Garcia A., Shirai T., Svec R. L., Hergenrother P. J. (2017). Predictive
Compound Accumulation
Rules Yield a Broad-Spectrum Antibiotic. Nature.

[42] Tsai K., Stojković V., Lee D. J., Young I. D., Szal T., Klepacki D., Vázquez-Laslop N., Mankin A. S., Fraser J. S., Fujimori D. G. (2022). Structural Basis for Context-Specific
Inhibition of Translation by Oxazolidinone Antibiotics. Nat. Struct. Mol. Biol..

[43] Syroegin E. A., Flemmich L., Klepacki D., Vazquez-Laslop N., Micura R., Polikanov Y. S. (2022). Structural
Basis for the Context-Specific
Action of the Classic Peptidyl Transferase Inhibitor Chloramphenicol. Nat. Struct. Mol. Biol..

[44] Flowers J., Echols N., Correy G., Jaishankar P., Togo T., Renslo A. R., van den
Bedem H., Fraser J. S., Wankowicz S. A. (2025). Expanding
automated multiconformer
ligand modeling to macrocycles and fragments. eLife.

[45] van
Zundert G. C. P., Hudson B. M., de Oliveira S. H. P., Keedy D. A., Fonseca R., Heliou A., Suresh P., Borrelli K., Day T., Fraser J. S. (2018). QFit-Ligand
Reveals Widespread Conformational Heterogeneity of Drug-like Molecules
in X-Ray Electron Density Maps. J. Med. Chem..

[46] Arenz S., Ramu H., Gupta P., Berninghausen O., Beckmann R., Vázquez-Laslop N., Mankin A. S., Wilson D. N. (2014). Molecular Basis for Erythromycin-Dependent Ribosome
Stalling during Translation of the ErmBL Leader Peptide. Nat. Commun..

[47] Svetlov M. S., Syroegin E. A., Aleksandrova E. V., Atkinson G. C., Gregory S. T., Mankin A. S., Polikanov Y. S. (2021). Structure
of Erm-Modified 70S Ribosome
Reveals the Mechanism of Macrolide Resistance. Nat. Chem. Biol..

[48] Crowe-McAuliffe C., Murina V., Turnbull K. J. (2022). Structural
basis for
PoxtA-mediated resistance to phenicol and oxazolidinone antibiotics. Nat. Commun..

