# Supplementary material for: Hybrid Antibiotics Targeting the Bacterial Ribosome
Source: ACS Cent Sci. 2025 Sep 17;11(11):2133–42. doi: 10.1021/acscentsci.5c01046 (PMC12670282; doi:10.1021/acscentsci.5c01046)
Supplement: Supplementary file 1 [file oc5c01046_si_001.pdf]

## Supporting Information for

# Hybrid Antibiotics Targeting the Bacterial Ribosome

Seul Ki Yeon,[1] Jenna Pellegrino,[2] Tushar Raskar,[2] Minh L. N. Tran,[1] Mohamad Dandan,[2] François Guérin,[3,4,5] Manuel Einsiedler,[6] Vincent Cattoir,[3,4,5] James S. Fraser,[2] Ian B. Seiple[1,6]*

1 – Department of Pharmaceutical Chemistry, Cardiovascular Research Institute, University of California, San Francisco, San Francisco, CA 94158.
2 – Department of Bioengineering and Therapeutic Sciences, University of California San Francisco, San Francisco, CA 94158.
3 – Department of Clinical Microbiology, University Hospital of Rennes, FR-35033 Rennes, France.
4 – National Reference Center for Antimicrobial Resistance (lab Enterococci), FR-35033 Rennes, France.
5 – Inserm U1230 BRM unit, University of Rennes, FR-35043 Rennes, France.
6 – Department of Chemistry, The Scripps Research Institute, La Jolla, CA 92037.

* to whom correspondence should be addressed: iseiple@scripps.edu

## Table of Contents

Table S1. Detailed MIC data for compounds **11**, **12**, **13** ..................... S3

Figure S1. Examples for previously described hybrid antibiotics ..................... S4

Figure S2. Mitochondrial biogenesis assay results ..................... S5

Figure S3. TDZ-desosamine hybrids lacking the macrolide macrocycle ..................... S6

Figure S4. Overlay of compound **13** in the ribosome with ABC-F protein ..................... S7

General experimental procedures, materials and instrumentation ..................... S8

Methods for biological assays ..................... S9

- Methods for in vitro translation 10-µM screen
- Methods for minimum inhibitory concentration (MIC) test
- Methods for mitochondrial biogenesis assay

Methods for CryoEM sample preparation and image reconstruction ..................... S10

Experimental procedures and data for synthetic compounds ..................... S11

- Synthesis of azithromycin analogs ..................... S11
- Synthesis of chloramphenicol analogs ..................... S12
- Synthesis of tedizolid analogs ..................... S16
- Synthesis of hybrids ..................... S19

NMR Spectra ..................... S26

Literature ..................... S54

## Table S1. Detailed MIC data for compounds 11, 12, 13

| No. | Species | Strain | AZI | ERY | CLI | SPI | LIN | TED | 11 | 12 | 13 |
|---|---|---|---|---|---|---|---|---|---|---|---|
| VCSA1 | *S. aureus* | ATCC29213 | 1 | 0.5 | 0.06 | 16 | 2 | 0.25 | 1 | 16 | 2 |
| VCSA2 | *S. aureus* | NCTC12493 | >64 | >64 | >64 | >64 | 2 | 0.12 | 64 | >64 | 1 |
| VCSA3 | *S. aureus* | BAA-976 (msrA) | >64 | >64 | 0.06 | 16 | 2 | 0.25 | 1 | 32 | 2 |
| VCSA4 | *S. aureus* | BAA-977 (ermA) | >64 | >64 | 0.06 | 8 | 2 | 0.25 | 1 | 16 | 2 |
| VCSA5 | *S. aureus* | HM1054/R (ermC constitutive) | >64 | >64 | >64 | >64 | 4 | 0.25 | >64 | >64 | 2 |
| VCSA6 | *S. aureus* | MLSaSb vat + , vatB -, vatC - ,vga - , vgaB - , vgb + , vgbB - | >64 | >64 | >64 | >64 | 2 | 0.12 | >64 | >64 | 1 |
| VCSA7 | *S. aureus* | MLSaSb vat - , vatB+, vatC - ,vga - , vgaB + , vgb - , vgbB - | >64 | >64 | >64 | >64 | 2 | 0.12 | >64 | >64 | 1 |
| VCSA8 | *S. aureus* | Mu3 | >64 | >64 | >64 | >64 | 4 | 0.12 | >64 | >64 | 1 |
| VCSA9 | *S. aureus* | Mu50 | >64 | >64 | >64 | >64 | 2 | 0.12 | 64 | >64 | 1 |
| VCSA10 | *S. aureus* | HM1055 ermA inducible | >64 | >64 | 0.06 | 4 | 2 | 0.12 | 1 | 16 | 1 |
| VCSA11 | *S. aureus* | ermA constitutive | >64 | >64 | >64 | >64 | 2 | 0.12 | >64 | >64 | 1 |
| VCSA12 | *S. aureus* | RN4220 ermC inducible (pAT28) | >64 | 32 | >64 | 4 | 2 | 0.25 | 0.5 | 16 | 1 |
| VCSA13 | *S. aureus* | ermC constitutive | >64 | >64 | >64 | >64 | 2 | 0.12 | >64 | >64 | 1 |
| VCSA14 | *S. aureus* | RN4220 (msrA) | >64 | >64 | 0.06 | 4 | 2 | 0.12 | 0.25 | 16 | 1 |
| VCSA15 | *S. aureus* | Mutation L4 | 16 | 8 | 0.06 | 64 | 2 | 0.12 | 2 | >64 | 1 |
| VCSA17 | *S. aureus* | Mutation L22 | 32 | 32 | 0.12 | >64 | 2 | 0.25 | 64 | >64 | 1 |
| VCSA18 | *S. aureus* | BM 3002 - BM3002 pIP524 vatA/vgaA/vgbA | 0.5 | 0.25 | 1 | 4 | 2 | 0.12 | 16 | >64 | 1 |
| VCSA19 | *S. aureus* | Mutation A2058G domain V 23S rRNA | >64 | >64 | 32 | >64 | 4 | 0.12 | 64 | >64 | 1 |
| VCSA20 | *S. aureus* | Mutation A2059G domain V 23S rRNA | >64 | >64 | 1 | >64 | 2 | 0.25 | 32 | >64 | 1 |
| | | | | | | | | | | | |
| VCEFL1 | *E. faecalis* | ATCC29212 | 8 | 4 | 32 | 2 | 2 | 0.25 | 2 | 64 | 1 |
| VCEFL2 | *E. faecalis* | erm(B) optrA | >64 | >64 | >64 | >64 | 16 | 1 | 64 | >64 | 2 |
| VCEFL3 | *E. faecalis* | emr(B) cfr optrA poxtA | >64 | >64 | >64 | >64 | 16 | 4 | >64 | >64 | 2 |
| | | | | | | | | | | | |
| VCEFM1 | *E. faecium* | HM1070 | 0.12 | 0.06 | <0.06 | 0.12 | 2 | 0.25 | 0.25 | 4 | 1 |
| VCEFM2 | *E. faecium* | erm(B) poxtA | >64 | >64 | >64 | >64 | 4 | 1 | >64 | >64 | 1 |
| VCEFM3 | *E. faecium* | erm(B) optrA poxtA | >64 | >64 | >64 | >64 | 16 | 2 | >64 | >64 | 1 |
| VCEFM4 | *E. faecium* | Aus0004 (vanB) | >64 | >64 | >64 | >64 | 2 | 0.12 | 64 | >64 | 1 |
| VCEFM5 | *E. faecium* | erm(B) | >64 | >64 | >64 | >64 | 4 | 0.25 | >64 | >64 | 1 |
| VCEFM6 | *E. faecium* | MLS inducible | 64 | 4 | 0.06 | 0.5 | 2 | 0.25 | 0.25 | 16 | 0.5 |
| VCEFM7 | *E. faecium* | Mutant eat(A)v (LSaP phenotype) | 0.12 | 0.06 | 2 | 0.5 | 2 | 0.12 | 8 | 8 | 1 |
| VCEFM8 | *E. faecium* | HM1032 (vatD, vanA, vgb, ermB) | >64 | >64 | >64 | >64 | 2 | 0.12 | 64 | >64 | 1 |
| VCEFM9 | *E. faecium* | erm(B) | >64 | >64 | >64 | >64 | 2 | 0.25 | >64 | >64 | 1 |
| VCEFM10 | *E. faecium* | erm(T) | >64 | >64 | >64 | >64 | 2 | 0.25 | >64 | >64 | 1 |
| VCEFM11 | *E. faecium* | Mutation G2576T 23S rRNA | >64 | >64 | >64 | >64 | 16 | 4 | >64 | >64 | 8 |
| | | | | | | | | | | | |
| SCSPN1 | *S. pneumoniae* | ATCC49619 | ≤0.06 | ND | ND | ND | ND | 0.12 | ≤0.06 | ND | 0.25 |
| SCSPN2 | *S. pneumoniae* | HM28 (erm(B)) | >64 | ND | ND | ND | ND | 0.12 | 0.25 | ND | 0.25 |
| | | | | | | | | | | | |
| VCSEpi1 | *S. epidermidis* | WT CIP8155_TS | 0.5 | 0.12 | 0.06 | 4 | 1 | 0.25 | 0.25 | 2 | 1 |
| VCSEpi2 | *S. epidermidis* | cfr | >64 | ND | ND | ND | ND | 64 | >64 | ND | >64 |

Minimum inhibitory concentrations (in µg/mL) for compounds **11**, **12**, **13**, and comparators in individual genetically characterized strains. Drug abbreviations: AZI, azithromycin; ERY, erythromycin; CLI, clindamycin; SPI, spiramycin; LIN, linezolid; TED, tedizolid.

# Figure S1. Examples for previously described hybrid antibiotics

D. Bulkley et al., *PNAS* **2010**, 17158–17163.
R. Hanselmann et al. (Rib-X), *OPRD* **2010**, 152–158.

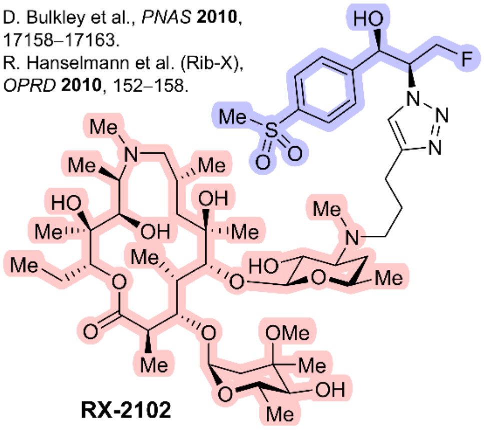

**RX-2102**

Azithromycin–Florfenicol

**MIC [µg/mL]**

| Strain | Azi | Flo | RX-2102 |
|---|---|---|---|
| *S. pneumoniae* ATCC49619 QC | ≤0.25 | 2 | ≤0.25 |
| *S. pneumoniae* 96-018535 (A2058G) | >128 | 2 | 0.5 |
| *S. pneumoniae* 02J1258 (*ermB*, L4) | >128 | 2 | 1 |

F. Franceschi & E.M. Duffy (Rib-X), *Biochem. Pharmacol.* **2006**, 1016–1025.

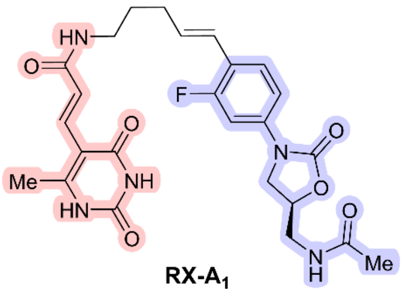

**RX-A$_1$**

Sparsomycin–Linezolid

| Strain | MIC [µg/mL] |
|---|---|
| *S. aureus* QC | 32 |
| *S. pneumoniae* 1175 (*mefa*) | 1 |
| *S. pyogenes* Msr610 (*ermB*) | 1 |
| *E. faecalis* P5 (LNZ-R G2576U) | 32 |
| *H. influenzae* parent strain RD1 | >128 |
| *H. influenzae* 895 (*acrB* KO) | 32 |

J. Zemlicka et al., *J. Med. Chem.* **1993**, 1239–1244.

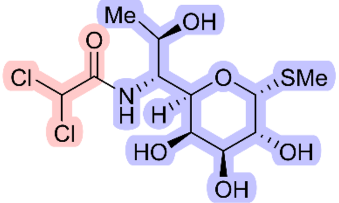

Chloramlincomycin

**MIC [µg/mL]**

| Strain | Chl | Linc | Hybrid |
|---|---|---|---|
| *S. aureus* | 6.25 | 0.78 | 50 |
| *E. faecalis* | 6.25 | 50 | >400 |
| *S. pyogenes* | 3.12 | 0.20 | 6.25 |
| *S. pneumoniae* | 3.12 | 0.78 | 25 |
| *E. coli* | 3.12 | >400 | >400 |
| *K. pneumoniae* | 3.12 | 400 | >400 |
| *S. schottmuelleri* | 6.25 | >800 | >400 |
| *P. aeruginosa* | 100 | >400 | >400 |

S.S. Daher et al., *Eur. J. Med. Chem.* **2022**, 114213.

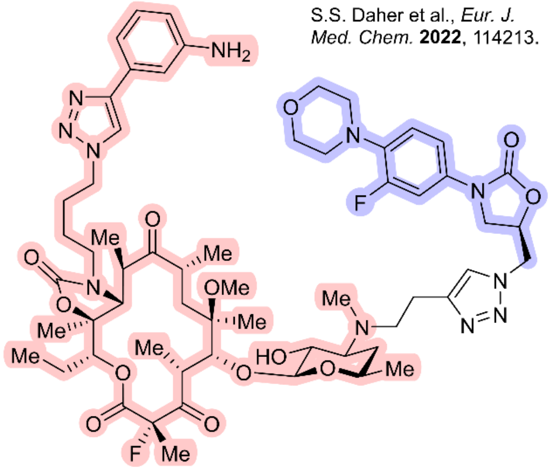

Solithromycin–Linezolid

**MIC [µM]**

| Strain | Sol | Lin | Hybrid |
|---|---|---|---|
| *S. aureus* MRSA ATCC43300 | >200 | 6.0 | 0.8 |
| *E. faecium* NCTC 7171 | 0.2 | 6.0 | 0.2 |
| *P. aeruginosa* HER 1018 | 30 | >200 | >200 |
| *K. pneumoniae* BAA 2146 | 100 | >200 | >200 |
| *A. baumannii* 2208 ATCC19606 | 50 | >200 | >200 |
| *E. coli* MG1655 | 100 | >200 | >200 |
| *E. cloacae* ATCC13047 | 200 | >200 | >200 |

**Figure S2. Mitochondrial biogenesis assay results**

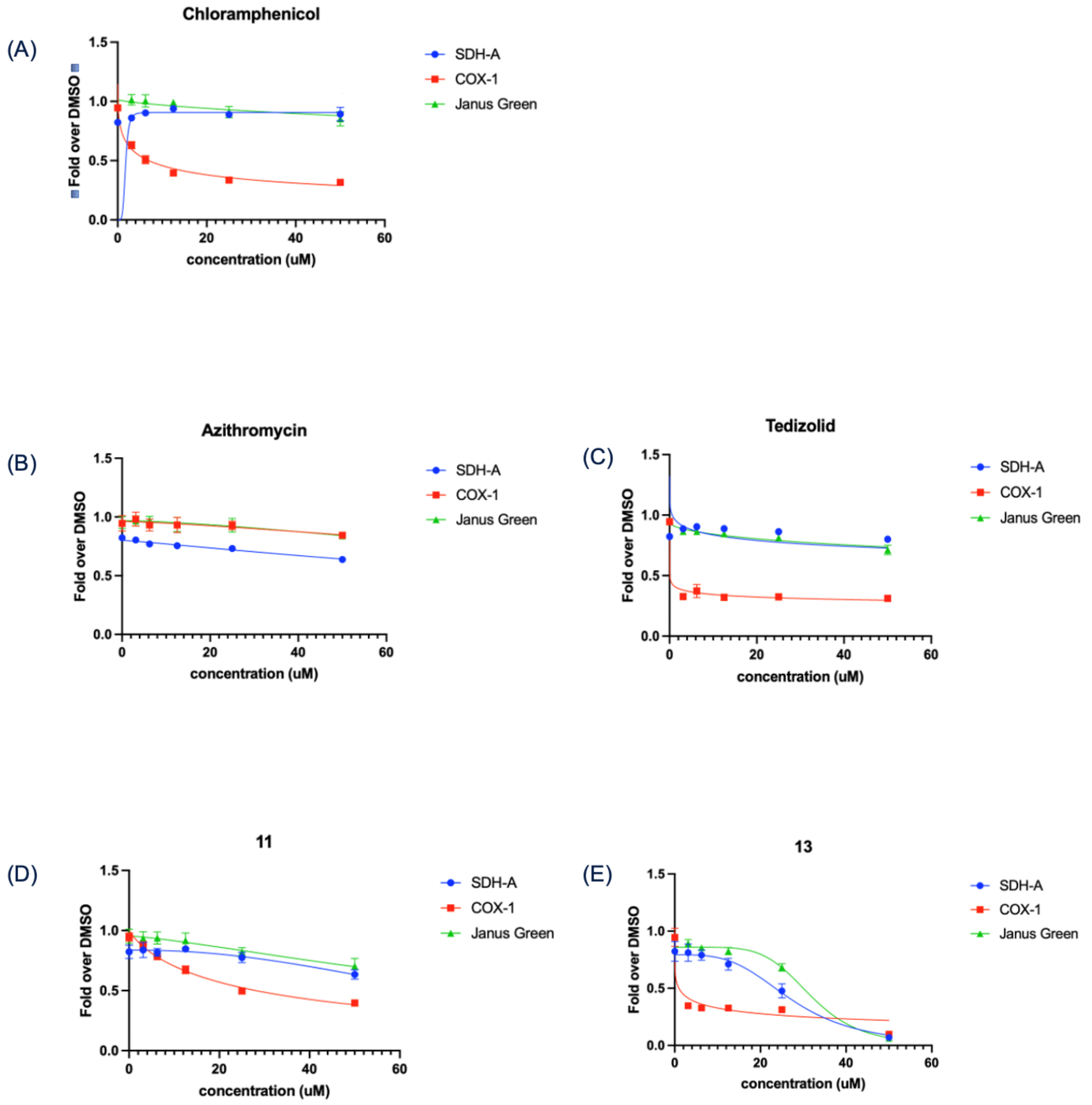


Mitobiogenesis assay (3–50 μM). Quantitative analysis of mitochondrial biogenesis assay upon treatment with (A) Chloramphenicol (**CHL**), (B) Azithromycin (**AZI**), (C) Tedizolid (**TDZ**), (D) compound **11**, and (E) compound **13**. Cells were treated with the indicated compounds at 50 μM, 25 μM, 12.5 μM, 6.25 μM, 3.125 μM, and 0 μM (DMSO vehicle). Mitochondrial biogenesis was assessed by SDH-A and COX-1 level; and staining with Janus green.

**Figure S3. TDZ-desosamine hybrids lacking the macrolide macrocycle**

The two following hybrids that lack the macrolide macrolactone were generated and characterized by cryo-EM in the *E. coli* ribosome:

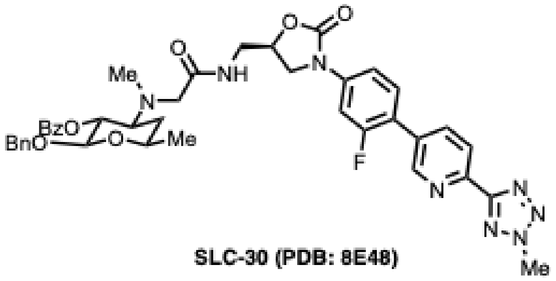

**SLC-30 (PDB: 8E48)**

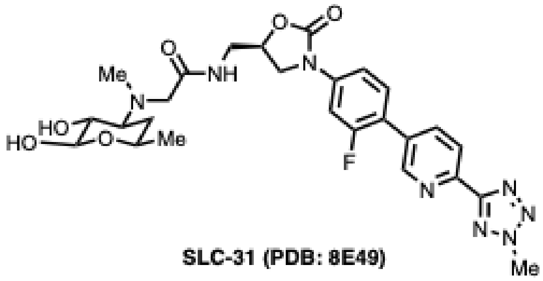

**SLC-31 (PDB: 8E49)**

**Figure S4. Overlay of compound 13 in the ribosome with ABC-F protein**

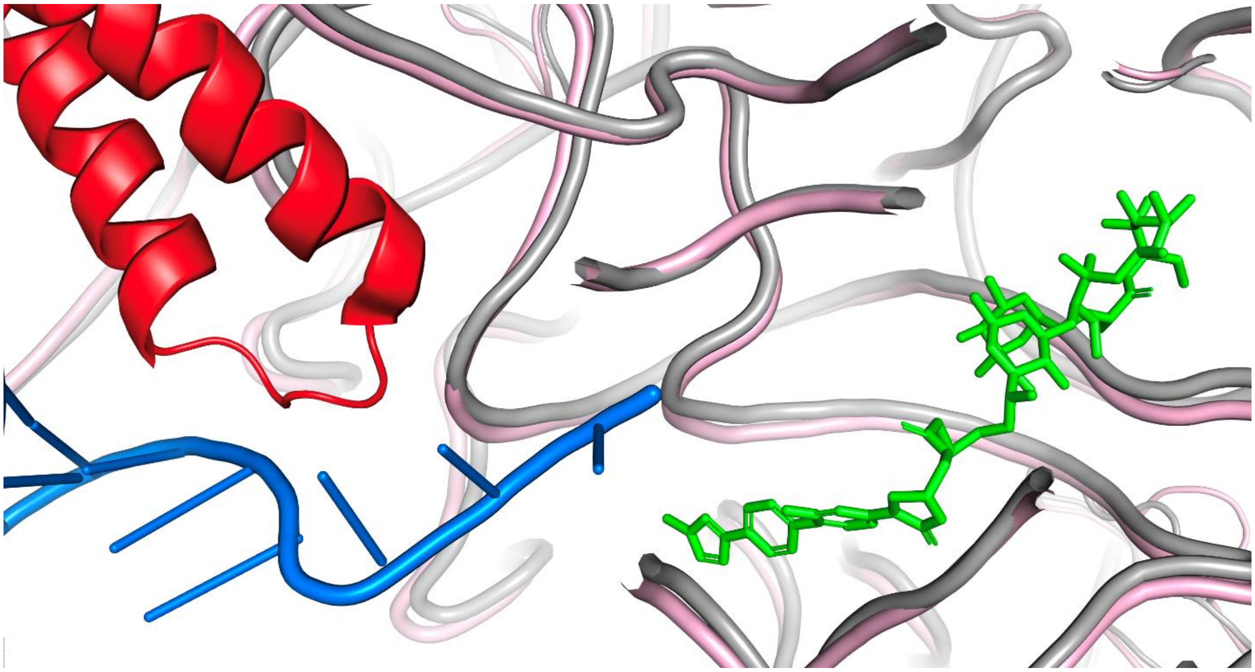

Overlay of the hybrid **13**-bound *E. coli* ribosome structure (PDB: 8E47) with the *E. faecalis* ribosome structure bound to P-site tRNA and the ABC-F resistance protein PoxtA (PDB: 7P7R) from *Nat. Commun.* **2022**, *13*, 1860. Hybrid **13** is green, *E. coli* ribosome is gray, *E. faecalis* ribosome is light pink, P-site tRNA is blue, and the Antibiotic Resistance Domain of PoxtA is red.

**General chemical synthesis procedures:** All reactions were performed in oven-dried glassware fitted with rubber septa under a positive pressure of nitrogen or argon, unless otherwise noted. Procedures were conducted at 23 ºC unless otherwise noted. All reaction mixtures were stirred throughout the duration of each procedure using Teflon-coated magnetic stir bars. Air- and moisture-sensitive liquids were transferred by means of syringe or stainless-steel cannula. Solutions were concentrated by rotary evaporation at or below 35 °C. Analytical thin-layer chromatography (TLC) was performed using glass plates pre-coated with silica gel (0.25-mm, 60-Å pore size, 230−400 mesh, SILICYCLE INC) with a fluorescent indicator (254 nm). TLC plates were visualized by exposure to ultraviolet light (UV) and then were stained by submersion in an acidic ethanolic solution of phosphomolybdic acid or ninhydrin followed by brief heating.

**Chemical synthesis materials:** Dichloromethane (DCM), tetrahydrofuran (THF), dimethylformamide (DMF), and acetonitrile (ACN) to be used in anhydrous reaction mixtures were dried by passage through activated alumina columns immediately prior to use. Other commercial solvents and reagents were used as received, unless otherwise noted. Anhydrous toluene, 2-propanol, methanol, ethyl acetate, and acetone were purchased from Fisher Chemical in Acroseal™ bottles. Anhydrous $^{i}Pr_2EtN$ (DIEA) and $Et_3N$ (TEA) were purchased from Sigma Aldrich in Sure/Seal™ bottles. Hexanes used were ≥85% *n*-hexane.

**Instrumentation:** Proton nuclear magnetic resonance ($^{1}H$ NMR) spectra and carbon nuclear magnetic resonance ($^{13}C$ NMR) spectra were recorded on 300 or 400 MHz Bruker Avance III HD 2-channel instrument NMR spectrometers at 23 °C or 50 ºC, and a AVIII HD 600 spectrometer at 23 °C (600 MHz, for 2D experiments, equipped with CPDCH CryoProbe). Fluorine nuclear magnetic resonance ($^{19}F$ NMR) spectra were recorded on a JEOL JNM-ECZ400R spectrometer. Proton chemical shifts are expressed in parts per million (ppm, δ scale, relative to TMS) and are referenced to residual protium in the NMR solvent ($CDCl_3$: δ 7.26, DMSO-$d_6$: δ 2.50 and MeOD-$d_4$: δ 3.31). Carbon chemical shifts are expressed in parts per million (ppm, δ scale, relative to TMS) and are referenced to the carbon resonance of the NMR solvent ($CDCl_3$: δ 77.0, DMSO-$d_6$: δ 39.52 and MeOD-$d_4$: δ 49.0). Fluorine chemical shifts are expressed in parts per million (ppm, δ scale, relative to $CFCl_3$) and are referenced by unified chemical shift scale. Data are represented as follows: chemical shift, multiplicity (s = singlet, d = doublet, t = triplet, q = quartet, dd = doublet of doublets, dt = doublet of triplets, m = multiplet, br = broad, app = apparent), integration, and coupling constant (*J*) in Hertz (Hz), assuming first-order spin-spin coupling. High-resolution mass spectra (HRMS) were obtained at the QB3/Chemistry Mass Spectrometry Facility at University of California, Berkeley using a Thermo LTQ-FT mass spectrometer or a Waters Acquity UPLC/Xevo G2-XS QTOF mass spectrometer.

**In vitro translation assay 10-µM screen.** The ability of experimental compounds to inhibit the 70S E. coli ribosome was first screened using the PURExpress® In Vitro Protein Synthesis Kit (E6800, NEB), murine RNAse inhibitor (M0314, NEB), and 6.66 ng/µl of template DNA encoding the fluorescent protein mEGFP (extracted by using E.N.Z.A. Plasmid DNA Mini Kit I, omega BIO-TEK). The volume of the reaction mixture was scaled down 5-fold from the NEB protocol for a final reaction volume of 5 µL. Analogs were screened at a final concentration of 10 µM in 10% DMSO. Translation reactions were carried out in triplicate at 37 °C for 1 hour, then transferred to a 0 °C metal block. To assist in the transfer of reactions to 96-well half-area Non-Binding Surface (NBS) microplates (Corning 3993) for final measurements, the reaction volume was increased to 50 µL by adding buffer (20 mM Tris-HCl pH 7.5, 60 mM $NH_4Cl$, 6 mM $MgCl_2$, 0.5 mM EDTA). Using a Cytation 5 plate reader (BioTek), translated mEGFP was excited at 485 nm; its emission was recorded at 535 nm. For comparison of analog activities across multiple initial screens, fluorescence readouts were normalized to the blank. Data were analyzed using Excel.

**Initial MIC screen in *S. aureus*.** *Staphylococcus aureus* Newman cultured and maintained in a Luria-Bertani (LB) agar plate. The MIC of the test compounds was determined using the microdilution method according to the CLSI standards. Cells were subcultured in MH-II broth until OD 0.2 to 0.6. The culture was diluted to $1\times10^5$ cells/mL and the resulting culture was plated at 90 µL per well in a 96-well assay plate. Separately, a test compound stock plate was prepared with serial 2-fold dilutions from 640–0.325 µg/mL in MH-II broth from parent DMSO stock solutions of each test compound (max DMSO concentration <5%). 10 µL of each well from the stock plate was added to corresponding wells in assay plate. The plate was covered with a breathable cover and incubated at 37 ºC at 225 rpm for 18 h. The turbidity of each well was examined, and the MIC was counted as the lowest concentration that lacked turbidity.

**MIC Panel assay against gram-positive pathogens for compounds 11, 12, and 13.** MICs were determined using the broth microdilution reference method according to the guidelines recommended by the European Committee on Antimicrobial Susceptibility (EUCAST; http://www.eucast.org/). Mueller-Hinton (MH; bioMérieux) broth and MH with lysed horse blood (5%) and β-NAD (20 mg/L) (MHF; bioMérieux) were used for staphylococci/enterococci and streptococci, respectively. The concentrations tested ranged from 0.06 to 64 mg/L. All experiments were performed in biological triplicate.

**Mitochondrial biogenesis assay.** The levels of two mitochondrial proteins were measured simultaneously using a colorimetric ELISA-kit, according to manufacturer's protocol (MitoBiogenesis™ In-Cell ELISA Kit- Abcamab110217. The two proteins are each subunit of a different oxidative phosphorylation enzyme complex, one protein being subunit I of Complex IV (Cytochrome c oxidase subunit-COX-IV), which is mitochondrial-DNA-encoded, and Succinate Dehydrogenase Complex Flavoprotein subunit A-SDHA) which is a 70kDa subunit of Complex II and nuclear-DNA-encoded. Details provided by the kit were followed with the general procedure as follows: PC-3 cells were plated in 96-well microplates at a density of 10,000 cells per 100 µL of culture medium in each cell (composed of 90% F-12K, 10% FBS, and 0.1% Penicillin-Streptomycin) and incubated overnight at 37 °C. A 50 µM stock of each test compound in culture medium was prepared from 10 mM parent DMSO stock solution. Cells were treated with 100 µL of experimental compound stock with two-fold serial dilution ranging from 50 µM to 3.13 µM. A vehicle control containing 0.5% DMSO in 100 µL culture medium was included for comparison. The plate was incubated for 72 h at 37 ºC. Cells were incubated with a primary antibody at 4 ºC shaker for overnight, followed by two hours of incubation with a secondary antibody at 23 ºC. Chloramphenicol was tested as a reference to validate the experiment.

**Cryo-EM sample preparation and data collection:** Samples were prepared using purified Ribosomes from *E.coli* MRE600 as stated previously[1]. The dataset was collected with an FEI Talos Arctica electron microscope (200 kV, Thermo Fisher, UCSF cryo-EM core facility) using a nine-shot beam image-shift approach with coma compensation[2]. Automated data collection was carried out using SerialEM (v4.1)[3]. All image stacks were collected in non-super resolution mode.

**Cryo-EM image and data processing:** The image stacks were binned by a factor of two, motion corrected, and dose-weighted using UCSF motioncor2[4]. Dose-weighted micrographs were used to determine contrast transfer function parameters using CTFFIND4[5]. Template picker was used to pick particles corresponding to the 70S ribosome. The structure of 7K00 was used to generate a reference map which was used as an input for generation of 2D classes for the template picker [6]. Box size for particle extraction was decided based on the criterion used in previous works[7]. 2D classification was carried out and only the particles that clearly contained ice were omitted. Homogeneous refinement was carried out using particles corresponding to good classes with both CTF and defocus refinements turned on[8].

**Atomic model building and refinement:** UCSF chimeraX was used to rigid body align 7K00 structure into our map. The initial restraints for ligands were generated using eLBOW[9] within Phenix[10]. Model refinement was carried out using manual model building in Coot[11] and phenix.real_space_refine within Phenix[10]. We combined real space refinement in Phenix with OPLS3e/VSGB2.1 force field based refinement[12] which allows a more accurate assignment of partial charges in case of small molecules. Full unsharpened maps were used for making the figures using PyMol Molecular Graphics System Version 3.0.2[13]

## Synthetic Procedures

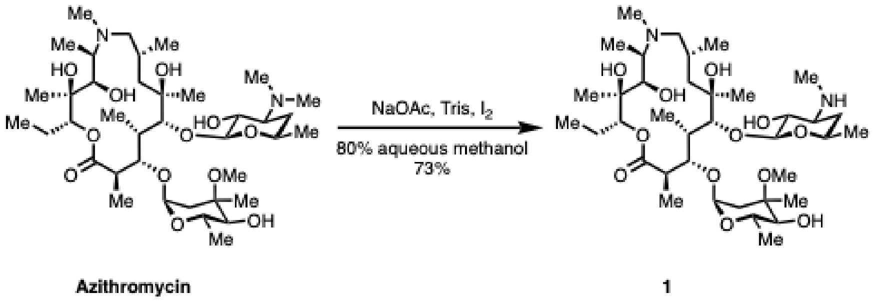

Sodium acetate (820 mg, 10.2 mmol, 1.50 equiv) was added in a single portion to a solution of azithromycin (5.00 g, 6.81 mmol, 1 equiv) in 4:1 methanol:water in (v:v, 68 mL) at 50 ºC. 2-Amino-2-(hydroxymethyl)-1,3-propanediol (Tris, 4.10 g, 34.1 mmol, 5.00 equiv) was added to maintain a reaction solution pH of 8-9 as measured by pH test paper (Fisher brand CAT. NO. 13-640-506). Solid iodine (1.70 g, 6.81 mmol, 1.00 equiv) was added in multiple portions to a reaction mixture resulting in a dark brown suspension. After 3 h, 10% aqueous sodium thiosulfate (50 mL) was added, and the biphasic mixture was stirred rapidly for 5 minutes. The dark brown suspension turned into a colorless mixture and was transferred to a separatory funnel. The mixture was extracted with DCM (2 × 150 mL). The combined organic layers were washed successively with water (2 × 30 mL) and saturated aqueous sodium chloride solution (50 mL), and the washed organic layer was dried ($Na_2SO_4$). The dried solution was filtered, and the filtrate was concentrated. The resulting crude residue was purified by flash chromatography (silica gel, eluent: DCM:MeOH:$NH_4OH$ = 9:0.9:0.1) to afford a product **1** (3.60 g, 73%) as an amorphous white solid.

**TLC** (DCM:MeOH:$NH_4OH$ = 9:0.9:0.1): $R_f$ = 0.38 (UV, PMA).

**HRMS-ESI** *m/z* calcd for $C_{37}H_{71}N_2O_{12}^+$ $[M + H]^+$ 735.5002, found 735.4983.

**$^1$H NMR** (400 MHz, $CDCl_3$) δ 5.11 (d, *J* = 4.7 Hz, 1H), 4.70 (dd, *J* = 9.9, 2.6 Hz, 1H), 4.41 (d, *J* = 7.5 Hz, 1H), 4.24 (dd, *J* = 4.6, 2.0 Hz, 1H), 4.08 (dq, *J* = 9.4, 6.2 Hz, 1H), 3.66 (d, *J* = 1.7 Hz, 1H), 3.63 (d, *J* = 6.9 Hz, 1H), 3.56 (ddq, *J* = 11.1, 6.1, 1.8 Hz, 1H), 3.33 (s, 3H), 3.21 (dd, *J* = 9.7, 7.5 Hz, 1H), 3.03 (d, *J* = 9.4 Hz, 1H), 2.79 – 2.64 (m, 2H), 2.59 – 2.48 (m, 2H), 2.43 (s, 3H), 2.34 (d, *J* = 15.2 Hz, 1H), 2.32 (s, 3H), 2.11 – 1.92 (m, 4H), 1.88 (dqd, *J* = 15.1, 7.6, 2.6 Hz, 1H), 1.74 (d, *J* = 14.5 Hz, 1H), 1.57 (dd, *J* = 15.2, 4.9 Hz, 1H), 1.46 (ddq, *J* = 14.5, 10.1, 7.3 Hz, 1H), 1.32 (d, *J* = 6.2 Hz, 3H), 1.31 (s, 3H), 1.26 – 1.17 (m, 2H),* 1.24 (s, 3H), 1.21 (d, *J* = 6.2 Hz, 3H), 1.19 (d, *J* = 7.5 Hz, 3H), 1.09 (d, *J* = 7.7 Hz, 3H), 1.08 (s, 3H), 0.98 (d, *J* = 7.5 Hz, 3H), 0.91 (d, *J* = 5.5 Hz, 3H), 0.88 (t, *J* = 7.8 Hz, 3H).
*Overlapping signal.

**$^{13}$C{$^1$H} NMR** (100 MHz, $CDCl_3$) δ 178.62, 102.37, 94.75, 83.92, 78.06, 77.95, 77.50, 74.50, 74.20, 73.75, 73.59, 72.96, 70.01, 68.40, 65.50, 62.45, 60.48, 49.40, 45.28, 42.30, 41.88, 37.04, 36.28, 34.67, 33.02, 27.40, 26.70, 21.95, 21.57, 21.22, 21.10, 18.19, 16.21, 14.81, 11.21, 9.48, 7.37.

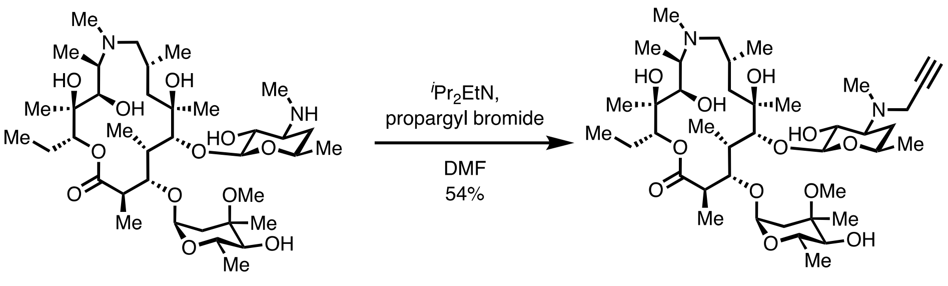


**1** **2**

Propargyl bromide (62.1 μL, 0.817 mmol, 2.00 equiv) and DIEA (214 μL, 1.22 mmol, 3.00 equiv) were added sequentially to a solution of **1** (300 mg, 0.408 mmol, 1.00 equiv) in anhydrous DMF (4 mL) at room temperature. The mixture was heated to 45 ºC resulting in a brown suspension. After 2 h, DCM (30 mL) was added. The mixture was transferred to a separatory funnel and washed with water (2 × 30 mL) and saturated aqueous sodium chloride solution (50 mL), and the organic layer was dried ($Na_2SO_4$). The dried solution was filtered, and the filtrate was concentrated. The resulting crude residue was purified by flash chromatography (silica gel, eluent: DCM:MeOH 17:3) to afford a product **2** (170 mg, 54%) as an amorphous white solid.

**TLC** (DCM:MeOH = 17:3): $R_f$ = 0.39 (UV, PMA).

**HRMS-ESI** *m/z* calcd for $C_{40}H_{73}N_2O_{12}^+$ [M + H]$^+$ 773.5163, found 773.5155.

**$^1$H NMR** (500 MHz, $CDCl_3$) δ 5.03 (d, *J* = 4.7 Hz, 1H), 4.68 (d, *J* = 9.2 Hz, 1H), 4.45 (d, *J* = 7.3 Hz, 1H), 4.25 (dd, *J* = 5.4, 2.3 Hz, 1H), 4.05 (dq, *J* = 12.5, 6.3 Hz, 1H), 3.69 (bs, 1H), 3.63 (d, *J* = 6.7 Hz, 1H), 3.57 – 3.47 (m, 1H), 3.41 (dd, *J* = 16.8, 2.5 Hz, 1H), 3.36 (dd, *J* = 16.8, 2.5 Hz, 1H), 3.33 (s, 3H), 3.25 (dd, *J* = 10.1, 7.3 Hz, 1H), 3.16 (bs, 1H), 3.02 (app t, *J* = 9.7 Hz, 1H), 2.84 – 2.67 (m, 3H), 2.52 (d, *J* = 11.8 Hz, 1H), 2.37 (bs, 3H), 2.34 (s, 3H), 2.33 (d, *J* = 15.2 Hz, 1H), 2.22 (t, *J* = 2.4 Hz, 1H), 2.19 (d, *J* = 10.3 Hz, 1H), 2.07 – 1.92 (m, 2H), 1.88 (dqd, *J* = 14.3, 7.5, 2.3 Hz, 1H), 1.81 – 1.75 (m, 2H), 1.56 (dd, *J* = 15.2, 4.9 Hz, 1H), 1.47 (ddq, *J* = 14.5, 9.5, 7.1 Hz, 1H), 1.36 – 1.25 (m, 2H)*, 1.32 (s, 3H), 1.30 (d, *J* = 6.8 Hz, 3H), 1.23 (s, 3H), 1.20 (d, *J* = 6.1 Hz, 3H), 1.18 (d, *J* = 7.4 Hz, 3H), 1.12 (d, *J* = 6.6 Hz, 3H), 1.07 (s, 3H), 1.03 (d, *J* = 7.5 Hz, 3H), 0.91 (d, *J* = 6.6 Hz, 3H), 0.88 (t, *J* = 7.5 Hz, 3H).
*Overlapping signal.

**$^{13}$C{$^1$H} NMR** (100 MHz, $CDCl_3$) δ 178.52, 102.85, 94.62, 83.55, 80.62, 78.08, 77.72, 77.30, 74.26, 73.75, 73.26, 72.91, 72.79, 70.82, 69.74, 68.64, 65.53, 64.13, 62.87, 49.41, 45.25, 43.84, 42.24, 42.15, 36.36, 35.78, 34.67, 30.88, 27.30, 26.60, 22.01, 21.56, 21.29, 21.26, 18.17, 16.29, 14.65, 11.21, 9.08, 7.64.

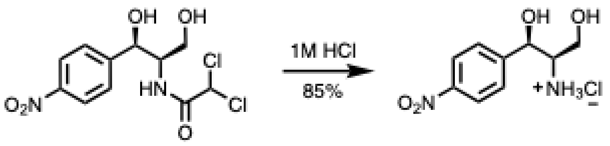


**Chloramphenicol** **S-1**

A solution of chloramphenicol (5.00 g, 15.5 mmol, 1 equiv) in aqueous 1M HCl (150 mL) was refluxed at 100 ºC for 2 h. The solution was cooled down to ambient temperature and was washed

with ether (4 × 50 mL). The washed aqueous layer was concentrated to afford a product **S-1** (3.27 g, 85%) as a white solid.

**TLC** (DCM:MeOH 8:2): $R_f$ = 0.15 (UV).

**HRMS-ESI** m/z calcd for $C_9H_{13}N_2O_4^+$ [M + H]$^+$ 213.0870, found 213.0868.

**$^1$H NMR** (400 MHz, MeOD-*d*$_4$): δ 8.32 – 8.25 (m, 2H), 7.77 – 7.71 (m, 2H), 4.97 (d, *J* = 8.0 Hz, 1H), 3.65 (dd, *J* = 11.7, 3.7 Hz, 1H), 3.47 (dd, *J* = 11.7, 5.6 Hz, 1H), 3.42 – 3.34 (m, 1H).

**$^{13}$C{$^1$H} NMR** (100 MHz, MeOD-*d*$_4$) δ 149.49, 149.38, 129.14 (2C), 124.76 (2C), 71.03, 59.84, 59.66.

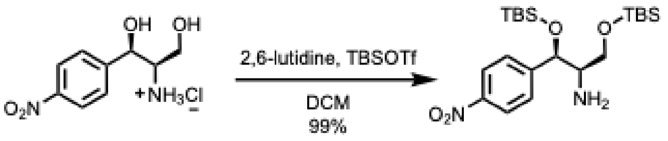


S-1 3

2,6-lutidine (419 μL, 3.62 mmol, 3.00 equiv) and *tert*-Butyldimethylsilyl trifluoromethanesulfonate (TBSOTf) (611 μL, 2.66 mmol, 2.20 equiv) were added sequentially to a solution of **S-1** (300 mg, 1.21 mmol, 1.00 equiv) in anhydrous DCM (12 mL) at room temperature (slightly exothermic). After 2 h, water (50 mL) was added. The clear biphasic mixture was transferred to a separatory funnel and was shaken vigorously and allowed to settle. The washed organic layer was dried ($Na_2SO_4$). The dried solution was filtered, and the filtrate was concentrated. The resulting crude residue was purified by flash chromatography (silica gel, eluent: DCM:hexane 1:3) to afford a product **3** (526 mg, 99%) as an amorphous white solid. The product was contaminated with an unidentified silyl byproduct, which was carried forward in the synthesis.

**TLC** (EtOAc:hexane = 1:3): $R_f$ = 0.38 (UV).

**HRMS-ESI** *m/z* calcd for $C_{21}H_{41}N_2O_4Si_2^+$ [M + H]$^+$ 441.2600, found 441.2593.

**$^1$H NMR** (400 MHz, $CDCl_3$) δ 8.22 – 8.16 (m, 2H), 7.50 – 7.45 (m, 2H), 4.91 (bd, *J* = 4.6 Hz, 1H), 3.54 (dd, *J* = 10.0, 6.2 Hz, 1H), 3.40 (dd, *J* = 10.0, 4.9 Hz, 1H), 2.74 (ddd, *J* = 6.2, 4.9, 4.6 Hz, 1H), 0.92 (s, 9H), 0.90 (s, 9H), 0.08 (s, 3H), 0.07 (s, 3H), 0.01 (s, 3H), -0.18 (s, 3H).

**$^{13}$C{$^1$H} NMR** (100 MHz, $CDCl_3$) δ 150.88, 147.25, 127.23 (2C), 123.38 (2C), 73.99, 63.71, 59.83, 25.85 (3C), 25.78 (3C), 18.14, 18.12, -2.98, -3.59, -4.54, -5.11.

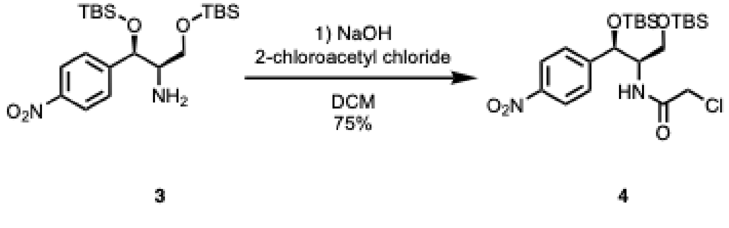


3 4

Solid sodium hydroxide (27.2 mg, 0.68 mmol, 1.00 equiv) and 2-chloroacetyl chloride (65.3 μL, 0.816 mmol, 1.20 equiv) were added sequentially to a solution of **3** (300 mg, 0.680 mmol, 1 equiv) in anhydrous DCM (6.8 mL) at 0 °C. The mixture was allowed to warm to 23 ºC and turned deep brown. After 2 h, water (50 mL) and DCM (20 mL) was added. The biphasic mixture was transferred to a separatory funnel and the layers were separated. The organic layer was washed with water (2 × 30 mL) and saturated aqueous sodium chloride solution (50 mL). The washed organic layer was dried ($Na_2SO_4$). The dried solution was filtered, and the filtrate was concentrated. The resulting crude residue was purified by flash chromatography (silica gel, eluent: EtOAc:hexane 1:3) to afford a product **4** (263 mg, 75%) as a yellow oil. The product was contaminated with an unidentified silyl byproduct, which was carried forward in the synthesis.

**TLC** (EtOAc:hexane = 1:3): $R_f$ = 0.55 (UV).

**HRMS-ESI** *m/z* calcd for $C_{23}H_{42}ClN_2O_5Si_2^+$ [M + H]$^+$ 517.2316, found 517.2306.

**$^1$H NMR** (400 MHz, $CDCl_3$) δ 8.23 – 8.15 (m, 2H), 7.50 – 7.42 (m, 2H), 7.13 (d, *J* = 8.0 Hz, 1H), 5.21 (bd, *J* = 2.5 Hz, 1H), 4.00 (d, *J* = 15.8 Hz, 1H), 4.00 – 3.94 (m, 1H), 3.92 (d, *J* = 15.8 Hz, 1H), 3.61 (dd, J = 9.9, 4.6 Hz, 1H), 3.56 (dd, *J* = 9.9, 8.5 Hz, 1H), 0.96 – 0.92 (m, 18H), 0.12 (s, 3H), 0.10 (s, 3H), 0.09 (s, 3H), -0.14 (s, 3H).

**$^{13}$C{$^1$H} NMR** (100 MHz, $CDCl_3$) δ 165.53, 149.85, 147.59, 126.95 (2C), 123.70 (2C), 70.76, 60.88, 57.37, 42.82, 25.95 (3C), 25.87 (3C), 18.23, 18.20, -3.45, -4.45, -5.14, -5.21.

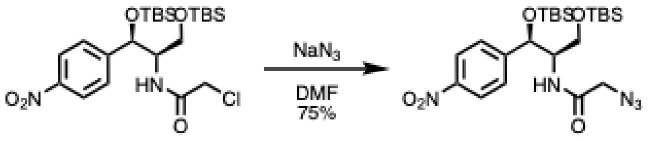


**4** **5**

Sodium azide (104 mg, 1.57 mmol, 3.00 equiv) was added to a solution of **4** (270 mg, 0.523 mmol, 1 equiv) in anhydrous DMF (5.2 mL) resulting in a brown suspension. The reaction mixture was stirred at 90 °C for 3 h. After 3 h, water was added. The biphasic mixture was transferred to a separatory funnel, was shaken vigorously, and allowed to settle. The layers were separated, and the organic phase was washed with water (2 × 30 mL) and saturated aqueous sodium chloride solution (50 mL), and the extracted organic layer was dried ($Na_2SO_4$). The dried solution was filtered, and the filtrate was concentrated. The resulting crude residue was purified by flash chromatography (silica gel, eluent: EtOAc:hexane 1:3) to afford a product **5** (205 mg, 75%) as a yellow oil.

**TLC** (EtOAc:hexane = 1:3): $R_f$ = 0.60 (UV).

**HRMS-ESI** *m/z* calcd for $C_{21}H_{42}N_5O_5Si_2^+$ [M + H]$^+$ 524.2719, found 524.2716.

**$^1$H NMR** (300 MHz, $CDCl_3$) δ 8.23 – 8.14 (m, 2H), 7.50 – 7.40 (m, 2H), 6.78 (d, *J* = 9.0 Hz, 1H), 5.19 (d, *J* = 2.6 Hz, 1H), 4.03 – 3.93 (m, 1H), 3.92 – 3.86 (m, 2H), 3.63 – 3.49 (m, 2H), 0.95 (s, 9H), 0.92 (s, 9H), 0.11 (s, 3H), 0.09 (s, 3H), 0.08 (s, 3H), -0.14 (s, 3H).

The product was contaminated with DMF after a short column chromatography. The product was carried forward in the synthesis.

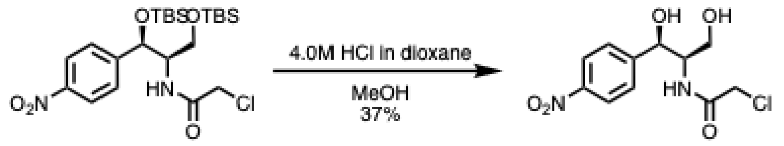


4 S-2

4.0 M HCl in dioxane (730 μL, 2.91 mmol, 10 equiv) was added to a solution of **4** (150 mg, 0.291 mmol, 1.0 equiv) in anhydrous methanol (3 mL) resulting in a brown suspension. After stirring at room temperature for 3 h, the reaction mixture was concentrated. The resulting crude residue was triturated with hexane to give a product **S-2** (31.0 mg, 37%) as a yellow oil.

**TLC** (EtOAc:hexane 1:3): $R_f$ = 0.20 (UV).

**HRMS-ESI** *m/z* calcd for $C_{11}H_{14}ClN_2O_5^+$ [M + H]$^+$ 289.0586, found 289.0585.

**$^1$H NMR** (400 MHz, MeOD-$d_4$) δ 8.22 – 8.15 (m, 2H), 7.67 – 7.60 (m, 2H), 5.14 (bd, *J* = 2.9 Hz, 1H), 4.16 (ddd, *J* = 6.9, 6.0, 2.9 Hz, 1H), 4.00 (d, *J* = 13.8 Hz, 1H), 3.94 (d, *J* = 13.8 Hz, 1H), 3.78 (dd, *J* = 10.9, 7.0 Hz, 1H), 3.60 (dd, *J* = 10.9, 6.0 Hz, 1H).

**$^{13}$C{$^1$H} NMR** (100 MHz, MeOD-$d_4$) δ 169.08, 151.73, 148.55, 128.28 (2C), 124.14 (2C), 71.41, 62.35, 58.18, 43.10.

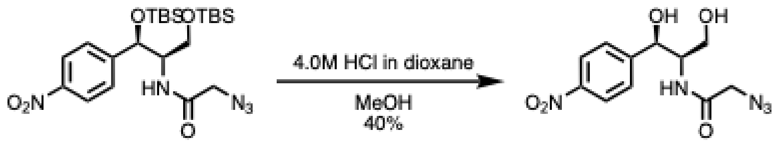


5 S-3

4.0M HCl in dioxane (730 μL, 2.86 mmol, 10.0 equiv) was added to a solution of **5** (150 mg, 0.286 mmol, 1 equiv) in anhydrous methanol (3 mL) resulting in a yellow suspension. After 3 h stirring at room temperature, the reaction mixture was concentrated. The resulting crude residue was triturated with hexane to give a product **S-3** (33.9 mg, 40%) as an amorphous brown solid.

**TLC** (EtOAc:hexane 1:3): $R_f$ = 0.20 (UV).

**HRMS-ESI** *m/z* calcd for $C_{11}H_{14}N_5O_5^+$ [M + H]$^+$ 296.0990, found 296.0987.

**$^1$H NMR** (400 MHz, MeOD-$d_4$) δ 8.23 – 8.12 (m, 2H), 7.69 – 7.58 (m, 2H), 5.14 (d, *J* = 2.7 Hz, 1H), 4.20 – 4.12 (m, 1H), 4.00 (d, *J* = 13.8 Hz, 1H), 3.93 (d, *J* = 13.8 Hz, 1H), 3.78 (dd, *J* = 11.0, 7.0 Hz, 1H), 3.60 (dd, *J* = 10.9, 6.0 Hz, 1H).

**$^{13}$C{$^1$H} NMR** (100 MHz, MeOD-$d_4$) δ 169.14, 151.76, 148.59, 128.31 (2C), 124.17 (2C), 71.43, 62.35, 58.21, 43.10.

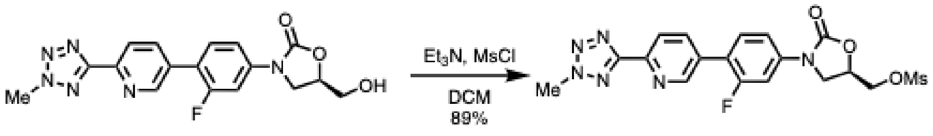


**Tedizolid** **S-4**

Triethylamine (5.64 mL, 40.5 mmol, 3.00 equiv) and anhydrous methanesulfonyl chloride (1.57 mL, 20.3 mmol, 1.50 equiv) were added sequentially to a solution of tedizolid (5.00 g, 13.5 mmol, 1 equiv) in anhydrous DCM (135 mL) at 0 ºC (slightly exothermic) resulting in a dark brown suspension. After 3 h, the reaction mixture was washed with saturated aqueous sodium bicarbonate solution (100 mL) and then aqueous 10% citric acid solution (100 mL). The organic layer was washed with saturated aqueous sodium chloride solution (50 mL) and dried ($Na_2SO_4$). The dried solution was filtered, and the filtrate was concentrated. The resulting crude residue was purified by flash chromatography (silica gel, eluent: ramp from pure EtOAc to EtOAc:MeOH 9:1) to afford a product **S-4** (5.40 g, 89%) as an off-white solid.

**TLC** (EtOAc): $R_f$ = 0.36 (UV).

**HRMS-ESI** *m/z* calcd for $C_{18}H_{18}FN_6O_5S^+$ [M + H]$^+$ 449.1038, found 449.1030.

**$^1$H NMR** (400 MHz, DMSO-$d_6$) δ 8.94 – 8.92 (m, 1H), 8.22 (dd, *J* = 8.2, 1.0 Hz, 1H), 8.18 (ddd, *J* = 8.3, 2.3, 1.3 Hz, 1H), 7.75 (app t, *J* = 8.8 Hz, 1H), 7.68 (dd, *J* = 13.6, 2.3 Hz, 1H), 7.51 (dd, *J* = 8.2, 1.0 Hz, 1H), 5.07 (dddd, *J* = 9.3, 6.1, 5.2, 3.0 Hz, 1H), 4.56 (dd, *J* = 11.5, 3.0 Hz, 1H), 4.51 (dd, J = 11.6, 5.2 Hz, 1H), 4.47 (s, 3H), 4.28 (app t, *J* = 9.3 Hz, 1H), 3.92 (dd, *J* = 9.4, 6.1 Hz, 1H), 3.28 (s, 3H).

**$^{13}$C{$^1$H}NMR** (100 MHz, DMSO-$d_6$) δ 163.92, 159.35 (d, *J* = 245.0 Hz), 153.73, 149.50 (d, *J* = 4.0 Hz), 145.16, 140.16 (d, *J* = 11.0 Hz), 137.27 (d, *J* = 3.0 Hz), 131.62 (d, *J* = 1.9 Hz), 131.04 (d, *J* = 4.4 Hz), 122.17, 119.04 (d, *J* = 13.0 Hz), 114.31 (d, *J* = 3.0 Hz), 105.72 (d, *J* = 29.0 Hz), 70.35, 69.76, 45.89, 39.89, 36.88.

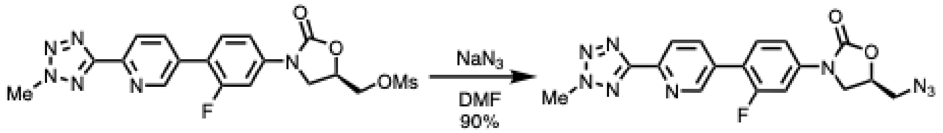


**S-4** **8**

Sodium azide (435 mg, 6.69 mmol, 3.00 equiv) was added to a solution of **S-4** (1.00 g, 2.23 mmol, 1 equiv) in anhydrous DMF (22 mL) and the resulting suspension and heated to 90 ºC. After 3 h, the mixture was cooled and DCM (50 mL) was added. The mixture was transferred to a separatory funnel and washed with water (3 × 50 mL) and saturated aqueous sodium chloride solution (50 mL), and the washed organic layer was dried ($Na_2SO_4$). The dried solution was filtered, and the filtrate was concentrated. The resulting crude residue was purified by flash chromatography (silica gel, eluent: ramp from EtOAc to EtOAc:MeOH 9:1) to afford a product **8** (794 mg, 90%) as a brown solid.

**TLC** (EtOAc:methanol 9:1): $R_f$ = 0.68 (UV).

**HRMS-ESI** *m/z* calcd for $C_{17}H_{15}FN_9O_2^+$ [M + H]$^+$ 396.1328, found 396.1321.

**$^1$H NMR** (400 MHz, DMSO-*d*$_6$) δ 8.94 – 8.92 (m, 1H), 8.24 (dd, *J* = 8.2, 1.0 Hz, 1H), 8.20 (ddd, *J* = 8.3, 2.3, 1.3 Hz, 1H), 7.76 (app t, *J* = 8.8 Hz, 1H), 7.69 (dd, *J* = 13.6, 2.3 Hz, 1H), 7.52 (dd, *J* = 8.2, 1.0 Hz, 1H), 4.95 (dddd, *J* = 9.3, 5.8, 5.8, 3.3 Hz, 1H), 4.47 (s, 3H), 4.22 (app t, *J* = 9.2 Hz, 1H), 3.86 (dd, *J* = 9.3, 6.0 Hz, 1H), 3.80 (dd, *J* = 13.5, 3.3 Hz, 1H), 3.73 (dd, *J* = 13.5, 5.5 Hz, 1H).

**$^{13}$C{$^1$H} NMR** (100 MHz, DMSO-*d*$_6$) δ 163.91, 159.36 (d, *J* = 246 Hz), 153.77, 149.51 (d, *J* = 4.2 Hz), 145.15, 140.24 (d, *J* = 11.4 Hz), 137.28 (d, *J* = 3.5 Hz), 131.65, 131.04 (d, *J* = 4.4 Hz), 122.17, 118.97 (d, *J* = 13.5 Hz), 114.27 (d, *J* = 3.2 Hz), 105.67 (d, *J* = 28.5 Hz), 71.54, 52.72, 46.91, 39.88.

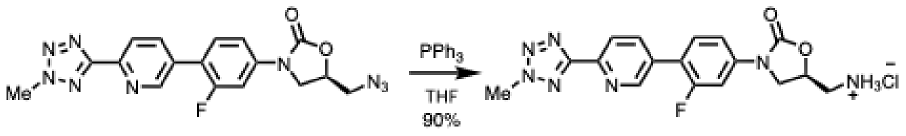


**8** **S-5**

Triphenylphosphine (9.95 g, 37.9 mmol, 3.00 equiv) was added to a solution of **8** (5.00 g, 12.6 mmol, 1 equiv) in THF (126 mL). The mixture was refluxed for 1 h. Water (12.6 mL) was added and refluxing was continued for additional 2 h. The reaction was cooled to room temperature and DCM (50 mL) was added. The biphasic mixture was transferred to a separatory funnel and extracted with aqueous 1 M HCl (2 x 50 mL). The acidic aqueous phases were combined and extracted with EtOAc (3 × 50 mL) to remove any unreacted triphenylphosphine and the by-product phosphine oxide ($Ph_3PO$). The washed aqueous layer was concentrated to afford a product **S-5** (4.62 g, 90%) as a yellow solid.

**TLC** (DCM:methanol 17:3): $R_f$ = 0.12 (UV).

**HRMS-ESI** *m/z* calcd for $C_{17}H_{17}FN_7O_2^+$ [M + H]$^+$ 370.1423, found 370.1420.

**$^1$H NMR** (400 MHz, MeOD-*d*$_4$) δ 9.07 (bs, 1H), 8.75 (ddd, *J* = 8.5, 2.2, 1.0 Hz, 1H), 8.65 (bd, *J* = 8.4 Hz, 1H), 7.81 (app t, *J* = 8.7 Hz, 1H), 7.81 (dd, *J* = 13.6, 2.2 Hz, 1H), 7.56 (dd, *J* = 8.6, 2.2 Hz, 1H), 5.07 (dddd, *J* = 9.3, 9.1, 6.5, 3.3 Hz, 1H), 4.56 (s, 3H), 4.38 (app t, *J* = 9.3 Hz, 1H), 3.98 (dd, *J* = 9.5, 6.5 Hz, 1H), 3.48 (dd, *J* = 13.8, 3.3 Hz, 1H), 3.41 (dd, *J* = 13.7, 9.1 Hz, 1H).

**$^{13}$C{$^1$H} NMR** (100 MHz, MeOD-*d*$_4$) δ 161.36 (d, *J* = 248.0 Hz), 161.25, 155.18, 145.54 (d, *J* = 5.2 Hz), 144.89 (d, *J* = 3.6 Hz), 142.88 (d, *J* = 11.4 Hz), 141.66, 136.12, 132.08 (d, *J* = 3.7 Hz), 125.56, 118.66 (d, *J* = 13.0 Hz), 115.80 (d, *J* = 3.0 Hz), 107.42 (d, *J* = 28.6 Hz), 71.34, 43.52, 40.65, 35.37.

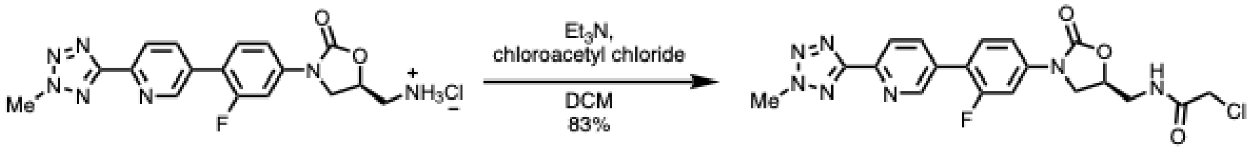

S-5 9

Triethylamine (721 μL, 5.17 mmol, 3.00 equiv) and chloroacetyl chloride (205 μL, 2.58 mmol, 1.50 equiv) were added sequentially to a solution of **S-5** (700 mg, 1.72 mmol, 1 equiv) in anhydrous DCM (19 mL) at 0 °C. The mixture was allowed to warm to ambient temperature resulting in a dark brown suspension. After 2 h, water (30 mL) was added. The biphasic mixture was transferred to a separatory funnel, and the layers were separated. The organic layer was washed with water (2 × 30 mL) and saturated aqueous sodium chloride solution (50 mL), and the washed organic layer was dried ($Na_2SO_4$). The dried solution was filtered, and the filtrate was concentrated. The resulting crude residue was purified by flash chromatography (silica gel, eluent: EtOAc:methanol 19:1) to afford a product **9** (638 mg, 83%) as a pale brown solid.

**TLC** (EtOAc:methanol 19:1): $R_f$ = 0.36 (UV).

**HRMS-ESI** *m/z* calcd for $C_{19}H_{18}ClFN_7O_3^+$ [M + H]$^+$ 446.1138, found 446.1132.

**$^1$H NMR** (400 MHz, DMSO-*d*$_6$) δ 8.94 – 8.91 (m, 1H), 8.65 (t, *J* = 5.9 Hz, 1H), 8.22 (dd, *J* = 8.2, 1.0 Hz, 1H), 8.18 (ddd, *J* = 8.3, 2.2, 1.3 Hz, 1H), 7.74 (app t, *J* = 8.9 Hz, 1H), 7.66 (dd, *J* = 13.6, 2.2 Hz, 1H), 7.48 (dd, *J* = 8.6, 2.3 Hz, 1H), 4.83 (ddt, J = 9.1, 6.4, 5.5 Hz, 1H), 4.47 (s, 3H), 4.21 (app t, *J* = 9.1 Hz, 1H), 4.11 (s, 2H), 3.83 (dd, *J* = 9.3, 6.4 Hz, 1H), 3.53 (app t, *J* = 5.5 Hz, 2H).

**$^{13}$C{$^1$H} NMR** (100 MHz, DMSO-*d*$_6$) δ 166.96, 163.90, 159.33 (d, *J* = 246 Hz), 153.96, 149.47 (d, *J* = 4.0 Hz), 145.11, 140.41 (d, *J* = 11.4 Hz), 137.24 (d, *J* = 3.6 Hz), 131.65 (d, *J* = 1.8 Hz), 130.96 (d, *J* = 4.3 Hz), 122.15, 118.82 (d, *J* = 13.4 Hz), 114.27 (d, *J* = 2.9 Hz), 105.66 (d, *J* = 28.4 Hz), 71.62, 47.16, 42.51, 41.84, 39.88.

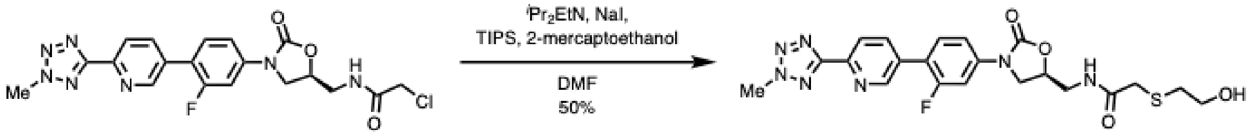

9 S-6

2-mercaptoethanol (30.6 μL, 0.437 mmol, 1.50 equiv), triisopropylsilyl chloride (62.5 μL, 0.292 mmol, 1.00 equiv), sodium iodide (43.8 mg, 0.292 mmol, 1.00 equiv) and diisopropylethylamine (153 μL, 0.876 mmol, 3.00 equiv) were added sequentially to a solution of **9** (130 mg, 0.292 mmol, 1 equiv) in anhydrous DMF (3 mL) resulting in a brown suspension. The mixture was stirred at 50 °C for 18 h. DCM (20 mL) was added and the mixture was transferred to a separatory funnel and washed with water (3 × 30 mL) and saturated aqueous sodium chloride solution (50 mL). The washed organic layer was dried ($Na_2SO_4$). The dried solution was filtered, and the filtrate was concentrated. The resulting crude residue was purified by flash chromatography (silica gel, eluent: EtOAc:methanol 19:1) to afford a product **S-6** (71.2 mg, 50%) as an off-white solid.

**TLC** (EtOAc:methanol 19:1): $R_f$ = 0.20 (UV).

**HRMS-ESI** *m/z* calcd for $C_{21}H_{23}FN_7O_4S^+$ [M + H]$^+$ 448.1511, found 448.1505.

**$^1$H NMR** (400 MHz, DMSO-*d*$_6$) δ 8.95 – 8.92 (m, 1H), 8.42 (t, *J* = 5.9 Hz, 1H), 8.24 (dd, *J* = 8.2, 1.0 Hz, 1H), 8.20 (ddd, *J* = 8.3, 2.2, 1.3 Hz, 1H), 7.75 (app t, *J* = 8.9 Hz, 1H), 7.68 (dd, *J* = 13.6, 2.3 Hz, 1H), 7.48 (dd, *J* = 8.6, 2.3 Hz, 1H), 4.86 – 4.77 (m, 1H), 4.47 (s, 3H), 4.20 (app t, *J* = 9.1 Hz, 1H), 3.82 (d, *J* = 9.3, 6.4 Hz, 1H), 3.53 – 3.46 (m, 3H), 2.59 (dd, *J* = 6.6, 6.1 Hz, 1H). Aliphatic proton signals overlap with solvent/water peak.

**$^{13}$C{$^1$H} NMR** (100 MHz, DMSO-*d*$_6$) δ 170.45, 163.95 , 159.36 (d, *J* = 244 Hz), 154.05, 149.51 (d, *J* = 4.0 Hz), 145.15, 140.47 (d, *J* = 11.0 Hz), 137.28 (d, *J* = 4.0 Hz), 131.71 (d, *J* = 1.7 Hz), 130.98 (d, *J* = 4.1 Hz), 122.20, 118.85 (d, *J* = 13.0 Hz), 114.27 (d, *J* = 2.9 Hz), 105.67 (d, *J* = 29.0 Hz), 71.86, 60.36, 47.14, 41.59, 39.90, 34.62, 34.50.

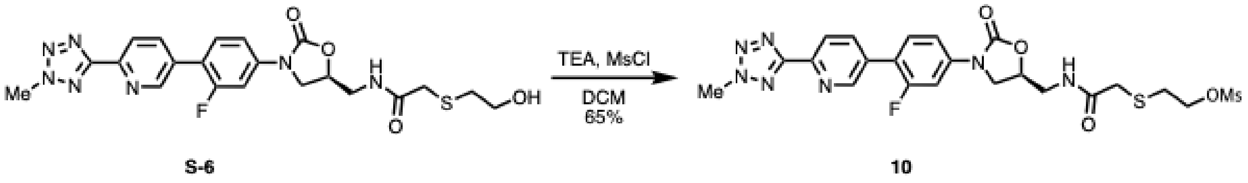


Triethylamine (114 μL, 0.82 mmol, 5.00 equiv) and methanesulfonyl chloride (38.1 μL, 0.492 mmol, 3.00 equiv) were added sequentially to a solution of **S-6** (80.0 mg, 0.164 mmol, 1 equiv) in anhydrous DCM (1.6 mL) at 0 ºC (slightly exothermic) resulting in a dark brown suspension. After 3 h, the mixture was diluted with DCM (30 mL) and washed with water (3 × 30 mL) and saturated aqueous sodium chloride solution (50 mL). The washed organic layer was dried ($Na_2SO_4$). The dried solution was filtered, and the filtrate was concentrated. The resulting crude residue provided **10** (60.3 mg, 65%) as an amorphous yellow solid and was directly consumed for the next step without purification. TLC (EtOAc:methanol 19:1): $R_f$ = 0.38 (UV).

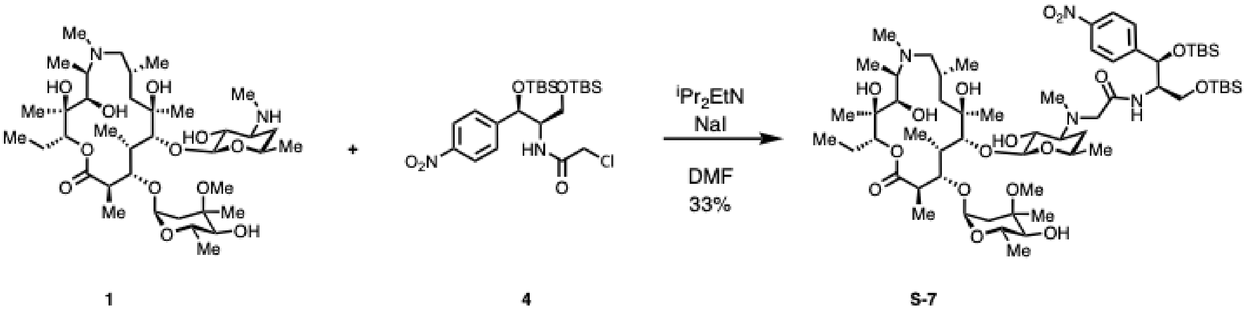


Compound **4** (253 mg, 0.490 mmol, 1.20 equiv), sodium iodide (61.2 mg, 0.408 mmol, 1.00 equiv), and diisopropylethylamine (213 μL, 1.22 mmol, 3.00 equiv) were added sequentially to a stirred solution of **1** (300 mg, 0.408 mmol, 1 equiv) in anhydrous DMF (4 mL). The mixture was heated to 45 ºC resulting in a brown suspension. After 18 h, the mixture was cooled and DCM (30 mL) was added. The biphasic mixture was transferred to a separatory funnel and washed with water (2 × 30 mL) and saturated aqueous sodium chloride solution (30 mL), and the extracted organic layer was dried ($Na_2SO_4$). The dried solution was filtered, and the filtrate was concentrated. The resulting crude residue was purified by flash chromatography (silica gel, eluent: DCM:MeOH 8:2) to afford a product **S-7** (164 mg, 33%) as an amorphous white solid.

**TLC** (DCM:MeOH:$NH_4OH$=19:0.9:0.1): $R_f$ = 0.35 (UV, PMA).

**HRMS-ESI** *m/z* calcd for $C_{60}H_{111}N_4O_{17}Si_2^+$ [M + H]$^+$ 1215.7478, found 1215.7447.

Due to broadening of the AZI portion in the $^1$H- and $^{13}$C-NMR spectra, the compound was carried forward for further characterization.

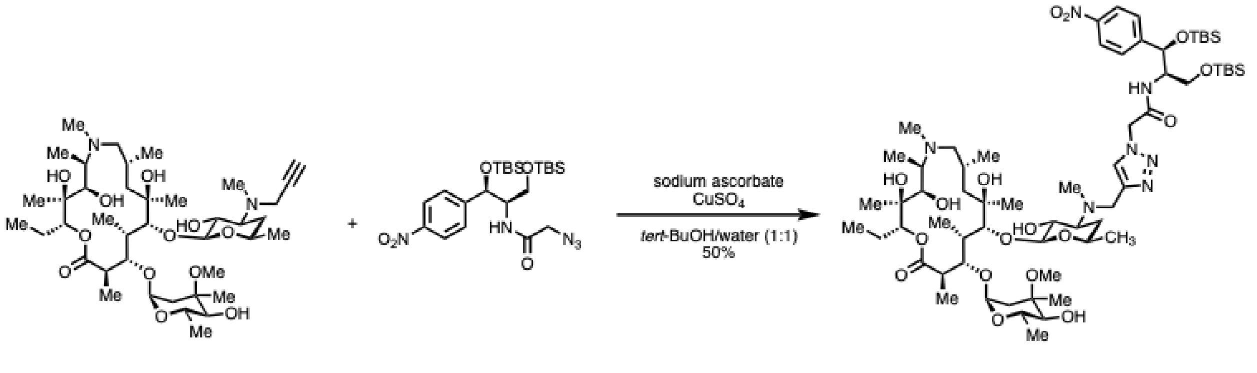


2 5 S-8

Sodium ascorbate (18.8 mg, 0.100 mmol, 1.00 equiv), copper (II) sulfate (1.52 mg, 0.0100 mmol, 0.100 equiv), and **2** (116 mg, 0.150 mmol, 1.50 equiv) were added to a solution of **5** (50.0 mg, 0.100 mmol, 1 equiv) in 1:1 *tert*-butanol and water (2 mL). The reaction mixture was stirred at room temperature for 2 h. The mixture was concentrated and purified by flash chromatography (silica gel, eluent: DCM:methanol 8:2) to afford a product **S-8** (64.8 mg, 50%) as an amorphous white solid.

**TLC** (DCM:methanol 8:2): $R_f$ = 0.40 (UV).

**HRMS-ESI** *m/z* calcd for $C_{63}H_{114}N_7O_{17}Si_2^+$ [M + H]$^+$ 1296.7804, found 1296.7787.

Due to broadening of the AZI portion in the $^1$H- and $^{13}$C-NMR spectra, the compound was carried forward for further characterization.

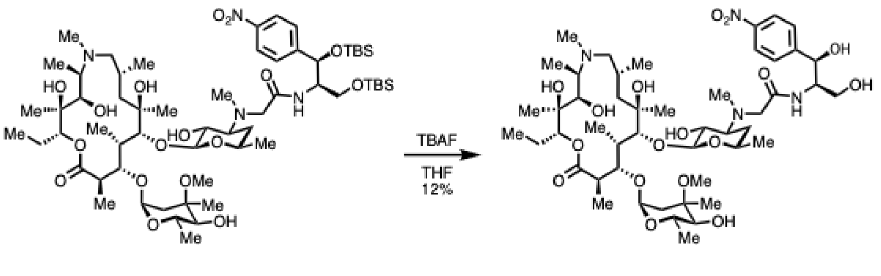


S-7 6

Tetrabutylammonium fluoride (53.8 mg, 0.206 mmol, 2.50 equiv) was added to a solution of **S-7** (100 mg, 0.0823 mmol, 1 equiv) in methanol (1.6 mL) resulting in a pale-yellow suspension. After 3 h stirring at room temperature, the reaction mixture was concentrated. The resulting crude residue was purified by flash chromatography (silica gel, eluent: DCM:MeOH 8:2) to afford a product **6** (9.74 mg, 12%) as an amorphous white solid.

**TLC** (DCM:MeOH:$NH_4OH$ = 19:0.9:0.1): $R_f$ = 0.17 (UV, PMA).

**HRMS-ESI** *m/z* calcd for $C_{48}H_{83}N_4O_{17}^+$ [M + H]$^+$ 987.5748, found 987.5732.

**$^1$H NMR** (300 MHz, MeOD-*d*$_4$) δ 8.19 (d, *J* = 8.8 Hz, 2H), 7.66 (d, *J* = 8.7 Hz, 2H), 5.13 (d, *J* = 3.2 Hz, 1H), 5.04 (d, *J* = 4.7 Hz, 1H), 4.51 (d, *J* = 7.3 Hz, 1H), 4.25 – 4.14 (m, 3H), 3.76 (dd, *J* = 11.1, 6.5 Hz, 2H), 3.69 – 3.54 (m, 3H), 3.40 – 3.32 (m, 6H), 3.25 – 3.14 (m, 2H), 3.06 (d, *J* = 9.5 Hz, 1H), 2.90 – 2.82 (m, 4H), 2.80 – 2.50 (m, 5H), 2.45 (d, *J* = 15.2 Hz, 2H), 2.25 (s, 3H), 2.23 – 2.07 (m, 1H), 1.91 (s, 3H), 1.79 (t, *J* = 13.8 Hz, 1H), 1.60 (dd, *J* = 15.3, 4.9 Hz, 1H), 1.55 – 1.45 (m, 2H), 1.40 (s, 3H), 1.36 (d, *J* = 6.7 Hz, 2H), 1.33 – 1.28 (m, 4H), 1.28 – 1.22 (m, 8H), 1.18 (d, *J* = 6.0 Hz, 3H), 1.10 (d, *J* = 7.5 Hz, 3H), 0.99 (d, *J* = 6.8 Hz, 3H), 0.90 (t, *J* = 7.3 Hz, 3H).
*5.49 (DCM), 3.35 (methanol)

**$^{13}$C{$^1$H} NMR** (100 MHz, MeOD-*d*$_4$) δ 178.92, 173.61, 150.97, 147.64, 127.55 (2C), 123.27 (2C), 103.23, 95.96, 83.60, 78.98, 78.36, 77.12, 74.89, 74.43, 73.95, 73.45, 72.14, 70.92, 68.84, 68.09, 65.83, 64.60, 61.72, 58.62, 57.48, 56.84, 49.24, 45.94, 42.46, 42.03, 37.61, 36.03, 35.05, 32.96, 26.18 (2C), 21.29 (2C), 21.03, 20.75, 18.14, 16.53, 14.87, 10.59, 9.10, 7.21.

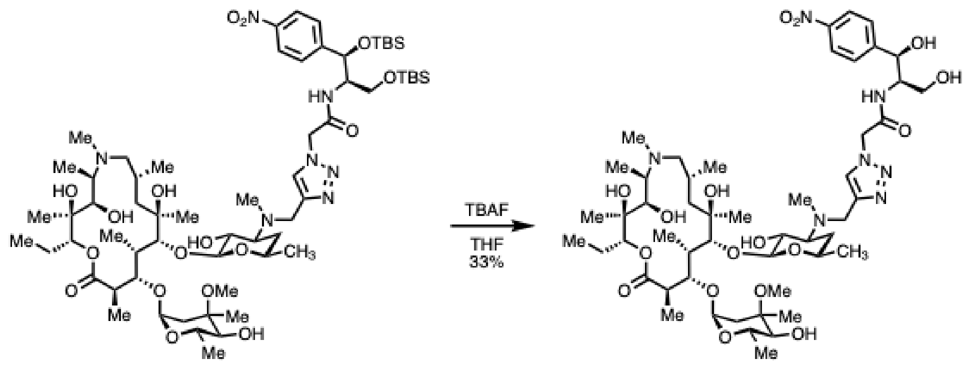


**S-8** **7**

Tetrabutylammonium fluoride (97.6 mg, 0.374 mmol, 2.20 equiv) was added to a solution of **S-8** (220 mg, 0.170 mmol, 1 equiv) in methanol (1.7 mL) resulting in a pale-yellow suspension. After 3 h stirring at room temperature, the reaction mixture was concentrated. The resulting crude residue was purified by flash chromatography (silica gel, eluent: DCM:MeOH 8:2) to afford a product **7** (59.9 mg, 33%) as an amorphous white solid.

**TLC** (DCM:MeOH:$NH_4OH$=19:0.9:0.1): $R_f$ = 0.20 (UV, PMA).

**HRMS-ESI** *m/z* calcd for $C_{51}H_{86}N_7O_{17}^+$ [M + H]$^+$ 1068.6075, found 1068.6054.

**$^1$H NMR** (400 MHz, MeOD-*d*$_4$) δ 8.15 (d, *J* = 8.8 Hz, 2H), 7.76 (s, 1H), 7.61 (d, *J* = 8.7 Hz, 2H), 5.11 (d, *J* = 2.8 Hz, 1H), 5.04 (dd, *J* = 6.9, 4.0 Hz, 3H), 4.90 (d, *J* = 4.0 Hz, 1H), 4.52 (d, *J* = 7.3 Hz, 1H), 4.24 – 4.10 (m, 3H), 3.91 – 3.81 (m, 1H), 3.82 – 3.77 (m, 1H), 3.73 (d, *J* = 13.9 Hz, 2H), 3.67 – 3.58 (m, 3H), 3.40 – 3.32 (m, 3H), 3.29 (d, *J* = 4.0 Hz, 1H), 3.25 (s, 3H), 3.03 (d, *J* = 9.5 Hz, 1H), 2.88 – 2.75 (m, 2H), 2.47 (s, 3H), 2.41 (d, *J* = 15.2 Hz, 1H), 2.26 (s, 3H), 2.07 (s, 1H),

2.03 – 1.96 (m, 1H), 1.91 – 1.75 (m, 3H), 1.59 (dd, $J$ = 15.2, 5.0 Hz, 1H), 1.53 – 1.39 (m, 3H), 1.37 (s, 3H), 1.29 (d, $J$ = 6.1 Hz, 4H), 1.24 (s, 3H), 1.22 (d, $J$ = 7.5 Hz, 4H), 1.19 (d, $J$ = 6.0 Hz, 5H), 1.12 (s, 3H), 1.06 (d, $J$ = 7.5 Hz, 3H), 1.03 –1.00 (m, 1H), 0.96 (d, $J$ = 6.8 Hz, 3H), 0.90 (t, $J$ = 7.4 Hz, 3H).
*3.35 (methanol)

**$^{13}C\{^1H\}$ NMR** (100 MHz, MeOD-$d_4$) δ 179.75, 167.72, 151.92, 148.51, 147.10, 128.33 (2C), 126.43, 124.15 (2C), 103.96, 96.63, 84.43, 79.80, 79.52, 78.07, 75.52, 75.43, 74.29, 72.57, 71.55, 69.15, 69.06, 66.61, 64.71, 62.52, 58.19, 53.05, 49.98, 46.75, 43.41, 36.82, 36.81, 35.99, 32.05, 27.49, 27.32, 22.24, 22.19, 21.98, 21.71, 19.04, 17.34, 15.65, 11.51, 9.83, 7.88.
* Four carbon signals not observed due to peak broadening.

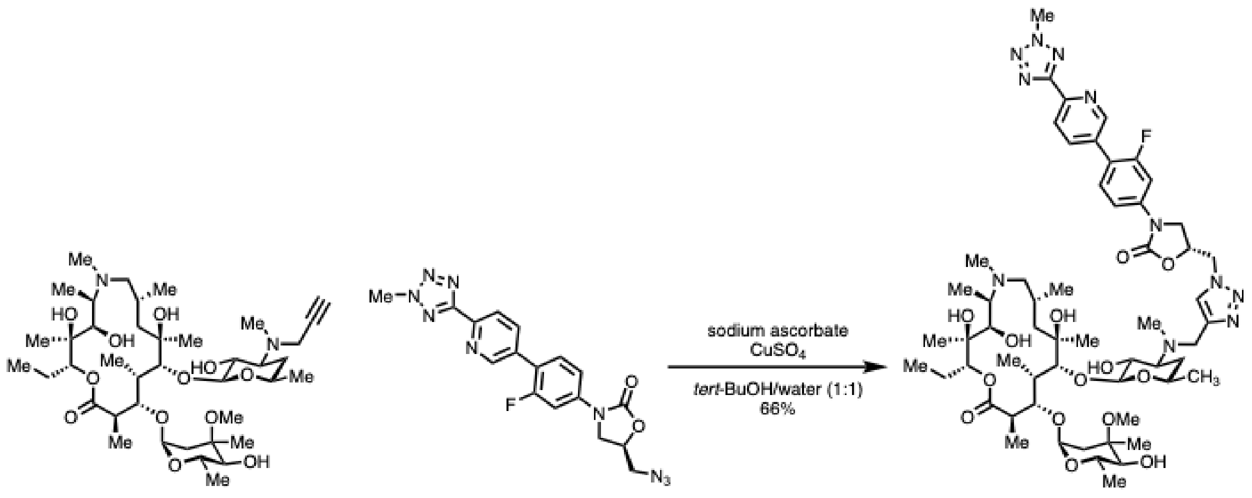


2 8 11

Sodium ascorbate (25.6 mg, 0.129 mmol, 1.00 equiv), copper (II) sulfate (10.3 mg, 0.0647 mmol, 0.500 equiv), and **8** (61.3 mg, 0.155 mmol, 1.20 equiv) were added sequentially to a solution of **2** (100 mg, 0.129 mmol) in 1:1 *tert*-butanol and water (1.3 mL) resulting in a brownish green suspension. The reaction mixture was stirred at room temperature for 2 h. After the reaction completed, the product mixture was concentrated and purified by flash chromatography (silica gel, eluent: DCM:methanol 8:2) to afford a product **11** (99.5 mg, 66%) as a pale yellow solid.

**TLC** (DCM:MeOH=8:2): $R_f$ = 0.10 (UV, PMA).

**HRMS-ESI** *m/z* calcd for $C_{57}H_{87}FN_{11}O_{14}^+$ [M + H]$^+$ 1168.6373, found 1168.6386.

**$^1H$ NMR** (600.1 MHz, $CDCl_3$ + drop of 50% (v/v) $NH_4OH_{aq}$) δ 8.93 – 8.88 (m, 1H), 8.30 (dd, $J$ = 8.3, 0.6 Hz, 1H), 8.03 (ddd, $J$ = 8.3, 2.1, 1.1 Hz, 1H), 7.71 (s, 1H), 7.54 – 7.46 (m, 2H), 7.25 (dd, $J$ = 8.5, 2.2 Hz, 1H), 5.17 (d, $J$ = 4.9 Hz, 1H), 5.21 – 5.14 (m, 1H), 4.77 – 4.72 (m, 2H)*, 4.68 (dd, $J$ = 9.9, 2.7 Hz, 1H), 4.47 (s, 3H), 4.42 (d, $J$ = 7.4 Hz, 1H), 4.26 – 4.19 (m, 2H), 4.13 – 4.00 (m, 2H), 3.87 (d, $J$ = 14.0 Hz, 1H), 3.69 – 3.57 (m, 3H), 3.53 – 3.46 (m, 1H), 3.35 – 3.30 (m, 1H), 3.30 (s, 3H), 3.03 (d, J = 9.4 Hz, 1H), 2.74 – 2.60 (m, 3H), 2.56 – 2.50 (m, 1H), 2.39 – 2.31 (m, 1H), 2.30 (s, 3H), 2.24 (s, 3H), 2.05 – 1.98 (m, 1H), 2.05 – 1.69 (m, 5H)*, 1.63 – 1.53 (m, 1H), 1.47 – 1.40 (m, 1H), 1.32 (d, $J$ = 6.3 Hz, 3H), 1.35 – 1.23 (m, 2H), 1.31 (s, 3H), 1.27 – 0.84 (m, 24H).
* Overlap with $NH_4OH_{aq}$ peaks.

**$^{13}C\{^1H\}$ NMR** (150.9 MHz, $CDCl_3$ + drop of 50% (v/v) $NH_4OH_{aq}$) δ 179.04, 164.65, 159.98 (d, $^1J$ = 249 Hz), 153.02, 149.82 (d, $^4J$ = 3.1 Hz), 146.98, 145.56, 139.06 (d, $^3J$ = 10.9 Hz), 137.06 (d, $^4J$ = 3.8 Hz), 132.00 (d, $^3J$ = 1.8 Hz), 130.66 (d, $^3J$ = 4.4 Hz), 123.88, 121.98, 120.81 (d, $^2J$ = 13.7 Hz), 113.87 (d, $^4J$ = 3.2 Hz), 106.56 (d, $^2J$ = 28.2 Hz), 102.89, 94.44, 83.50, 78.09, 77.72, 77.49, 74.06, 73.63, 73.29, 72.95, 70.95, 70.43, 70.00, 68.63, 65.58, 64.95, 62.55, 52.14, 49.41, 48.84, 47.10, 45.43, 42.52, 42.13, 39.74, 36.90, 36.04, 34.59, 30.44, 27.60, 26.69, 21.96, 21.57, 21.27, 21.26, 18.12, 16.26, 14.54, 11.18, 8.93, 7.00.

**$^{19}F\{^1H\}$ NMR** (376.5 MHz, $CDCl_3$ + drop of 50% (v/v) $NH_4OH_{aq}$) δ –114.09 (s, 0.6F), –114.10 (s, 0.4F).

Notes: In order to deprotonate potentially protonated amine groups, a drop of 50% (v/v) aqueous $NH_4OH$ was added directly into the NMR tube. This significantly narrowed the signals, see the spectra at the end of this document for comparison of $^1H$ NMR before and after $NH_4OH$ addition. A set of two conformers was observed (ratio ~0.6 to 0.4). Where distinguishable, $^1H$ signals of major conformer are given. Only $^{13}C$ shifts of major conformer are listed.

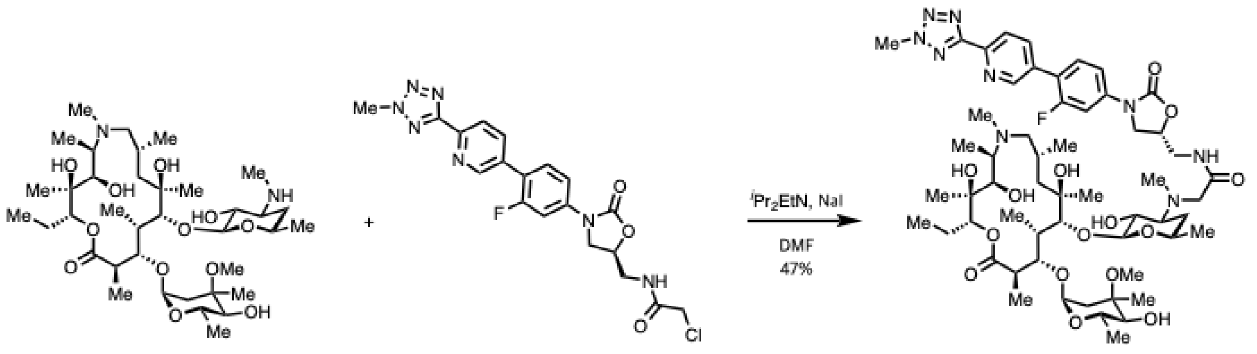


1 9 12

**9** (40.1 mg, 0.0899 mmol, 1.10 equiv), sodium iodide (12.2 mg, 0.0817 mmol, 1.00 equiv), and diisopropylethylamine (42.7 μL, 0.245 mmol, 3.00 equiv) were added to a stirred solution of **1** (60.0 mg, 0.0817 mmol, 1 equiv) in anhydrous DMF (1.6 mL) at room temperature and heated to 45 °C resulting in a dark brown suspension. After overnight reaction, DCM was added. The biphasic mixture was transferred to a separatory funnel and washed with water (2 × 5 mL) and saturated aqueous sodium chloride solution (5 mL), and the washed organic layer was dried ($Na_2SO_4$). The dried solution was filtered, and the filtrate was concentrated. The resulting crude residue was purified by flash chromatography (silica gel, eluent: DCM:MeOH 19:1) to afford a product **12** (43.9 mg, 47%) as a white solid.

**TLC** (DCM:MeOH=17:3): $R_f$ = 0.22 (UV, PMA).

**HRMS-ESI** *m/z* calcd for $C_{56}H_{87}FN_9O_{15}^+$ $[M + H]^+$ 1144.6300, found 1144.6273.

**$^1H$ NMR** (600.1 MHz, $CDCl_3$ + drop of 50% (v/v) $NH_4OH_{aq}$) δ 8.91 (ddd, *J* = 2.2, 1.1, 1.1 Hz, 1H), 8.39 (t, *J* = 6.4 Hz, 1H), 8.29 (dd, *J* = 8.2, 0.9 Hz, 1H), 8.03 (ddd, *J* = 8.2, 2.3, 1.4 Hz, 1H), 7.63 (dd, *J* = 12.8, 2.3 Hz, 1H), 7.50 (dd, *J* = 8.7, 8.5 Hz, 1H), 7.30 (dd, *J* = 8.6, 2.3 Hz, 1H), 5.12 (d, *J* = 4.8 Hz, 1H), 4.85 – 4.80 (m, 1H)*, 4.68 (dd, *J* = 10.0, 2.6 Hz, 1H), 4.46 (s, 3H), 4.35 (d, *J* = 7.3 Hz, 1H), 4.21 (dd, *J* = 3.8, 2.0 Hz, 1H), 4.09 (app t, *J* = 8.9 Hz, 1H), 4.02 (dq, *J* = 9.4, 6.2 Hz, 1H), 3.85 (dd, *J* = 9.1, 6.3 Hz, 1H), 3.72 (ddd, *J* = 14.2, 6.2, 3.7 Hz, 1H), 3.64 – 3.60 (m, 2H), 3.59

(d, $J$ = 7.1 Hz, 1H), 3.48 (ddd, $J$ = 10.8, 5.9, 1.8 Hz, 1H), 3.28 (d, $J$ = 17.1 Hz, 1H), 3.29 – 3.27 (m, 1H), 3.26 (s, 3H), 3.10 (d, $J$ = 17.1 Hz, 1H), 3.02 (d, $J$ = 9.3 Hz, 1H), 2.68 (qd, $J$ = 7.5, 3.8 Hz, 1H), 2.66 (qd, $J$ = 6.9, 1.2 Hz, 1H), 2.57 – 2.51 (m, 2H), 2.33 – 2.28 (m, 1H), 2.31 (s, 3H), 2.29 (s, 3H), 2.06 – 1.99 (m, 2H), 1.99 – 1.93 (m, 1H), 1.88 (dqd, $J$ = 15.0, 7.5, 2.6 Hz, 1H), 1.70 (ddd, $J$ = 13.0, 4.4, 2.0 Hz, 1H), 1.68 (bd, $J$ = 14.7 Hz, 1H), 1.56 (dd, $J$ = 15.2, 5.0 Hz, 1H), 1.43 (ddq, $J$ = 14.5, 9.9, 7.3 Hz, 1H), 1.30 (d, $J$ = 6.2 Hz, 3H), 1.28 (s, 3H), 1.27 – 1.22 (m, 2H), 1.21 (s, 3H), 1.19 (d, $J$ = 6.1 Hz, 3H), 1.16 (d, $J$ = 7.5 Hz, 3H), 1.08 (s, 3H), 1.07 (d, $J$ = 6.8 Hz, 3H), 0.95 (d, $J$ = 7.6 Hz, 3H), 0.90 (d, $J$ = 6.2 Hz, 3H), 0.86 (t, $J$ = 7.4 Hz, 3H).

**$^{13}$C{$^1$H} NMR** (150.9 MHz, $CDCl_3$) δ 178.78, 173.04, 164.63, 160.01 (d, $^1J$ = 249 Hz), 154.00, 149.76 (d, $^4J$ = 3.3 Hz), 145.50, 139.76 (d, $^3J$ = 10.9 Hz), 136.97 (d, $^4J$ = 3.8 Hz), 132.06 (d, $^3J$ = 1.6 Hz), 130.52 (d, $^3J$ = 4.4 Hz), 121.96, 120.24 (d, $^2J$ = 13.8 Hz), 113.69 (d, $^4J$ = 3.1 Hz), 106.33 (d, $^2J$ = 28.6 Hz), 102.72, 94.40, 83.48, 77.96, 77.59, 77.49, 74.02, 73.48, 73.24, 72.99, 72.13, 71.95, 69.92, 68.29, 65.59, 64.12, 62.55, 57.50, 49.31, 47.51, 45.37, 42.22, 42.17, 41.69, 39.72, 38.21, 36.04, 34.51, 32.58, 27.50, 26.63, 21.96, 21.51, 21.20, 21.11, 18.09, 16.22, 14.54, 11.14, 9.55, 7.04.

**$^{19}$F{$^1$H} NMR** (376.5 MHz, $CDCl_3$ + drop of 50% (v/v) $NH_4OH_{aq}$) δ –114.25 (s, 1F).

Notes: In order to deprotonate potentially protonated amine groups, a drop of 50% (v/v) aqueous $NH_4OH$ was added directly into the NMR tube. This significantly narrowed the signals, see the spectra at the end of this document for comparison of $^1$H NMR before and after $NH_4OH$ addition.

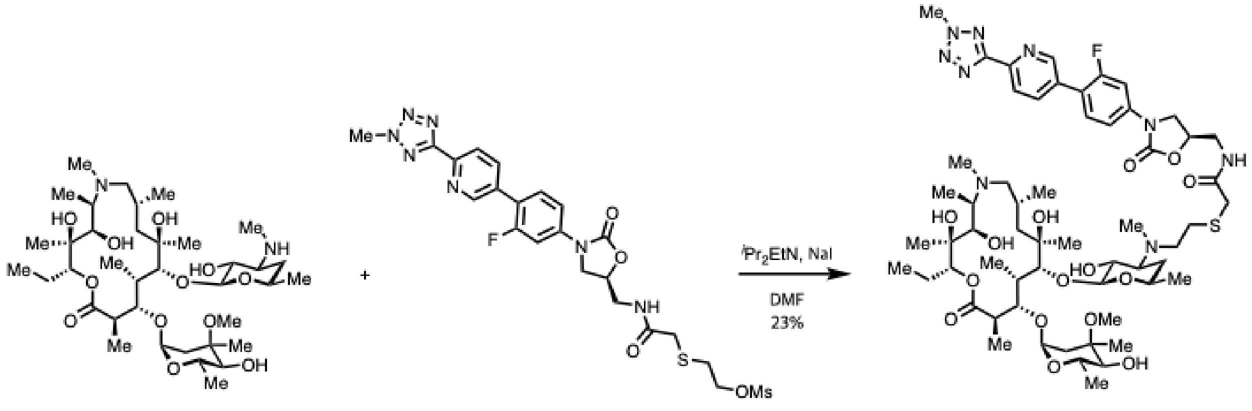


**1** **10** **13**

**1** (156 mg, 0.212 mmol, 1.20 equiv), sodium iodide (26.5 mg, 0.177 mmol, 1.00 equiv), and diisopropylethylamine (154 μL, 0.885 mmol, 5.00 equiv) were added to a solution of **10** (100 mg, 0.177 mmol, 1 equiv) in anhydrous DMF (1.8 mL). The mixture was heated to 45 ºC resulting in a dark brown suspension. After 18 h, DCM (10 mL) was added. The biphasic mixture was transferred to a separatory funnel and washed with water (2 × 10 mL) and saturated aqueous sodium chloride solution (10 mL), and the washed organic layer was dried ($Na_2SO_4$). The dried solution was filtered, and the filtrate was concentrated. The resulting crude residue was purified by flash chromatography (silica gel, eluent: DCM:MeOH 19:1) to afford a product **13** (49.0 mg, 23%) as a white solid.

**TLC** (DCM:MeOH=17:3): $R_f$ = 0.28 (UV, PMA).

**HRMS-ESI** *m/z* calcd for $C_{58}H_{91}FN_9O_{15}S^+$ [M + H]$^+$ 1204.6339, found 1204.6329.

**$^{1}$H NMR** (600.1 MHz, $CDCl_3$): δ 8.93 – 8.88 (m, 1H), 8.30 (dd, *J* = 8.3, 0.6 Hz, 1H), 8.04 (ddd, *J* = 8.3, 2.1, 1.1 Hz, 1H), 7.77 (t, *J* = 6.1 Hz, 1H), 7.65 (dd, *J* = 12.8, 2.3 Hz, 1H), 7.51 (dd, *J* = 8.6, 8.6 Hz, 1H), 7.33 (dd, *J* = 8.6, 2.3 Hz, 1H), 5.16 (d, *J* = 4.9 Hz, 1H), 4.88 – 4.83 (m, 1H), 4.68 (dd, *J* = 9.9, 2.7 Hz, 1H)*, 4.46 (s, 3H), 4.41 (d, *J* = 7.4 Hz, 1H), 4.25 (dd, *J* = 3.0, 2.2 Hz, 1H), 4.13 – 4.03 (m, 2H), 3.86 (dd, *J* = 9.1, 6.1 Hz, 1H), 3.76 (ddd, *J* = 14.4, 6.2, 5.6 Hz, 1H), 3.67 (ddd, *J* = 14.4, 5.6, 4.5 Hz, 1H), 3.65 – 3.60 (m, 1H), 3.59 (d, *J* = 7.5 Hz, 1H), 3.52 – 3.46 (m, 1H), 3.33 (s, 3H), 3.32 (d, *J* = 16.0 Hz, 1H), 3.24 (dd, *J* = 10.1, 7.3 Hz, 1H), 3.21 (d, *J* = 16.0 Hz, 1H), 3.03 (d, *J* = 9.6 Hz, 1H), 2.78 – 2.73 (m, 1H), 2.74 – 2.59 (m, 4H), 2.57 – 2.48 (m, 3H), 2.38 – 2.30 (m, 1H), 2.29 (s, 3H), 2.26 (s, 3H), 2.09 – 1.83 (m, 4H), 1.75 – 1.63 (m, 2H), 1.57 (dd, *J* = 15.1, 5.0 Hz, 1H), 1.47 – 1.40 (m, 1H), 1.32 (d, *J* = 6.3 Hz, 3H), 1.29 (s, 3H), 1.34 – 1.24 (m, 2H), 1.24 (s, 3H), 1.20 (d, *J* = 6.0 Hz, 3H), 1.14 – 1.04 (m, 9H), 0.95 (d, *J* = 7.5 Hz, 3H), 0.91 (d, *J* = 6.7 Hz, 3H), 0.86 (t, *J* = 7.4 Hz, 3H).
* Overlap with $NH_4OH_{aq}$ peaks.

**$^{13}$C{$^{1}$H} NMR** (150.9 MHz, $CDCl_3$ + drop of 50% (v/v) $NH_4OH_{aq}$) δ 179.02, 170.45, 164.63, 160.03 (d, $^{1}J$ = 249 Hz), 153.89, 149.77 (d, $^{4}J$ = 3.3 Hz), 145.52, 139.78 (d, $^{3}J$ = 11.1 Hz), 136.98 (d, $^{4}J$ = 4.3 Hz), 132.07 (d, $^{3}J$ = 1.7 Hz), 130.58 (d, $^{3}J$ = 4.4 Hz), 121.98, 120.27 (d, $^{2}J$ = 13.8 Hz), 113.66 (d, $^{4}J$ = 2.8 Hz), 106.27 (d, $^{2}J$ = 28.5 Hz), 102.77, 94.37, 83.37, 78.07, 77.56, 77.46, 74.04, 73.56, 73.23, 72.97, 71.40, 70.84, 69.85, 68.64, 65.76, 65.58, 62.48, 52.09, 49.49, 47.44, 45.44, 42.56, 42.34, 42.15, 39.73, 37.27, 36.30, 36.00, 34.56, 32.24, 30.19, 27.61, 26.66, 21.98, 21.55, 21.26, 21.24, 18.12, 16.25, 14.49, 11.17, 8.90, 6.95.

**$^{19}$F{$^{1}$H} NMR** (376.5 MHz, $CDCl_3$ + drop of 50% (v/v) $NH_4OH_{aq}$) δ –114.19 (s, 1F).

Notes: In order to deprotonate potentially protonated amine groups, a drop of 50% (v/v) aqueous $NH_4OH$ was added directly into the NMR tube. This significantly narrowed the signals, see the spectra at the end of this document for comparison of $^{1}$H NMR before and after $NH_4OH$ addition. A set of two conformers was observed (ratio ~0.6 to 0.4). Where distinguishable, $^{1}$H signals of major conformer are given. Only $^{13}$C shifts of major conformer are listed.

## NMR Spectra

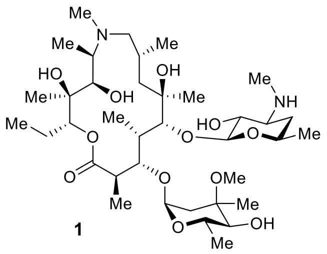

**1**

NMR solvent: $CDCl_3$
Field strength: 400 MHz ($^1H$)
100 MHz ($^{13}C$)

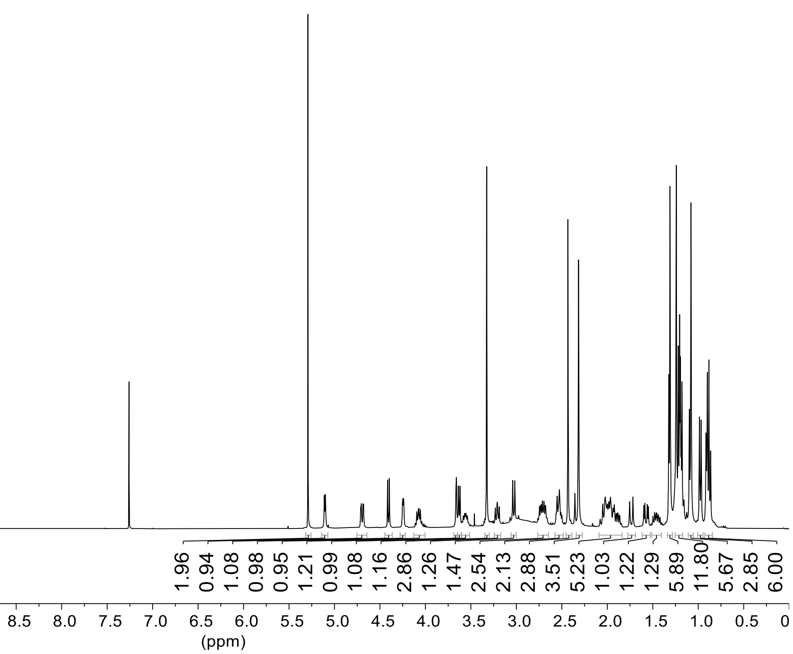


Figure S5. $^1H$-NMR spectrum of compound **1**.

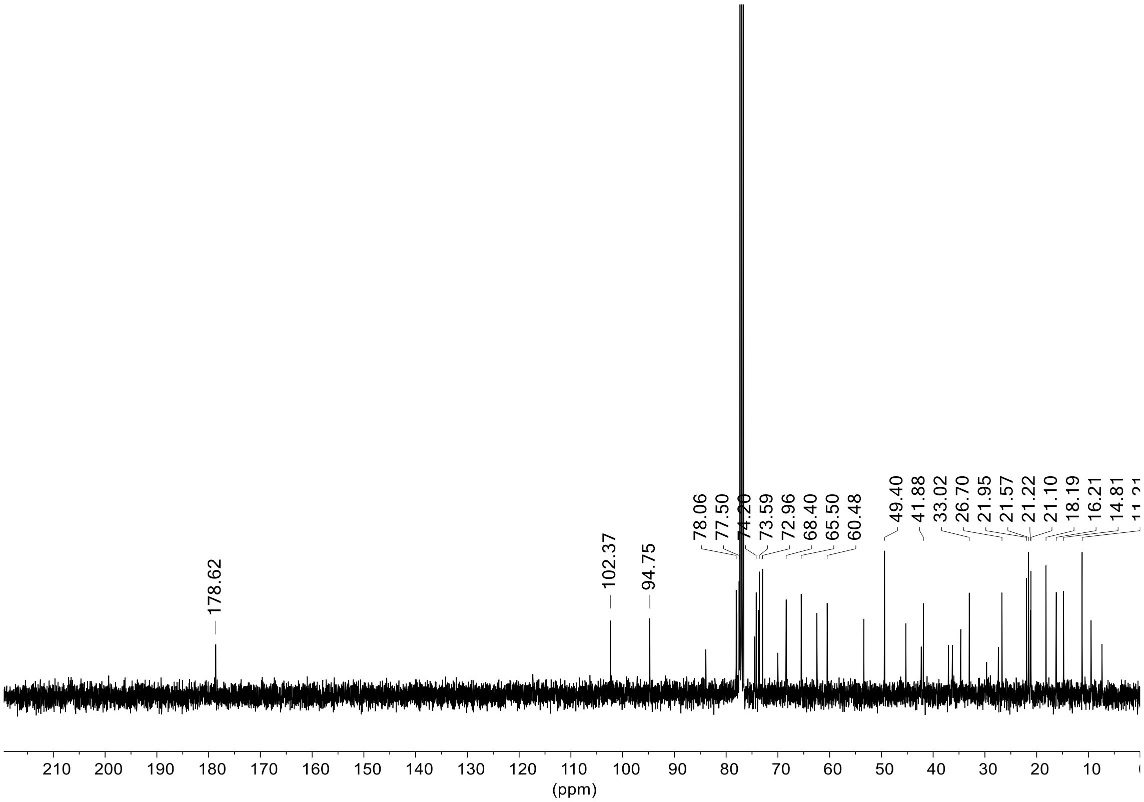


Figure S6. $^{13}C\{^1H\}$-NMR spectrum of compound **1**.

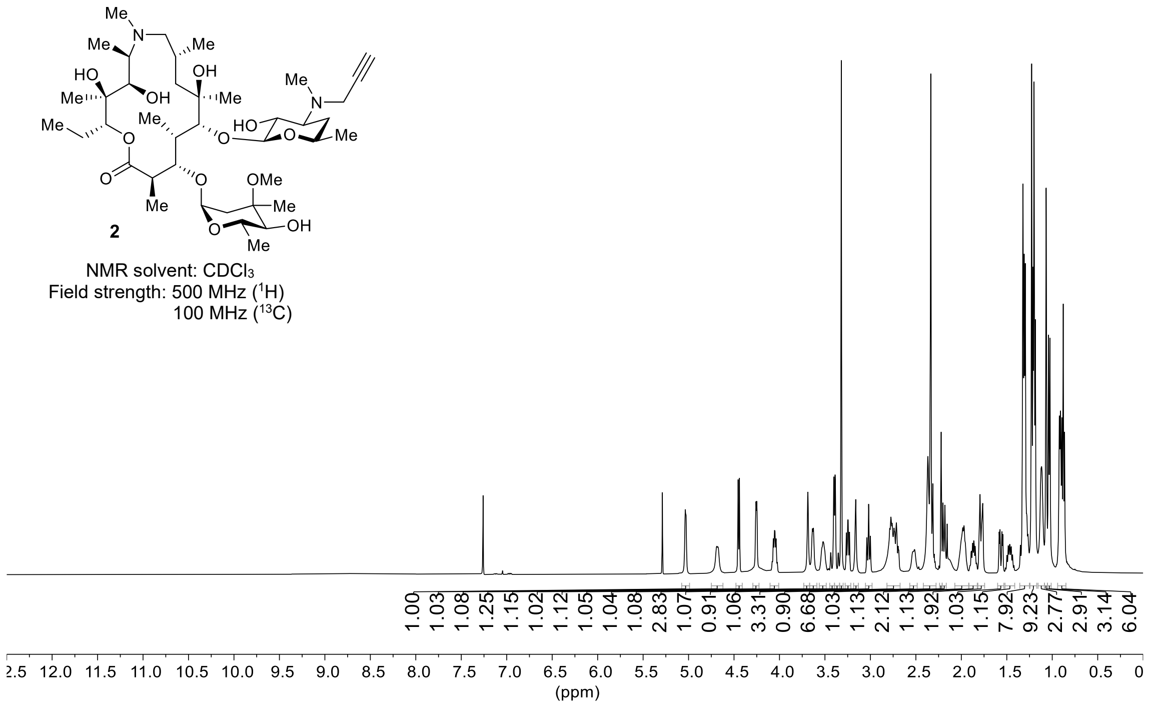

**2**

NMR solvent: $CDCl_3$
Field strength: 500 MHz ($^1H$)
100 MHz ($^{13}C$)



Figure S7. $^1H$-NMR spectrum of compound **2**.

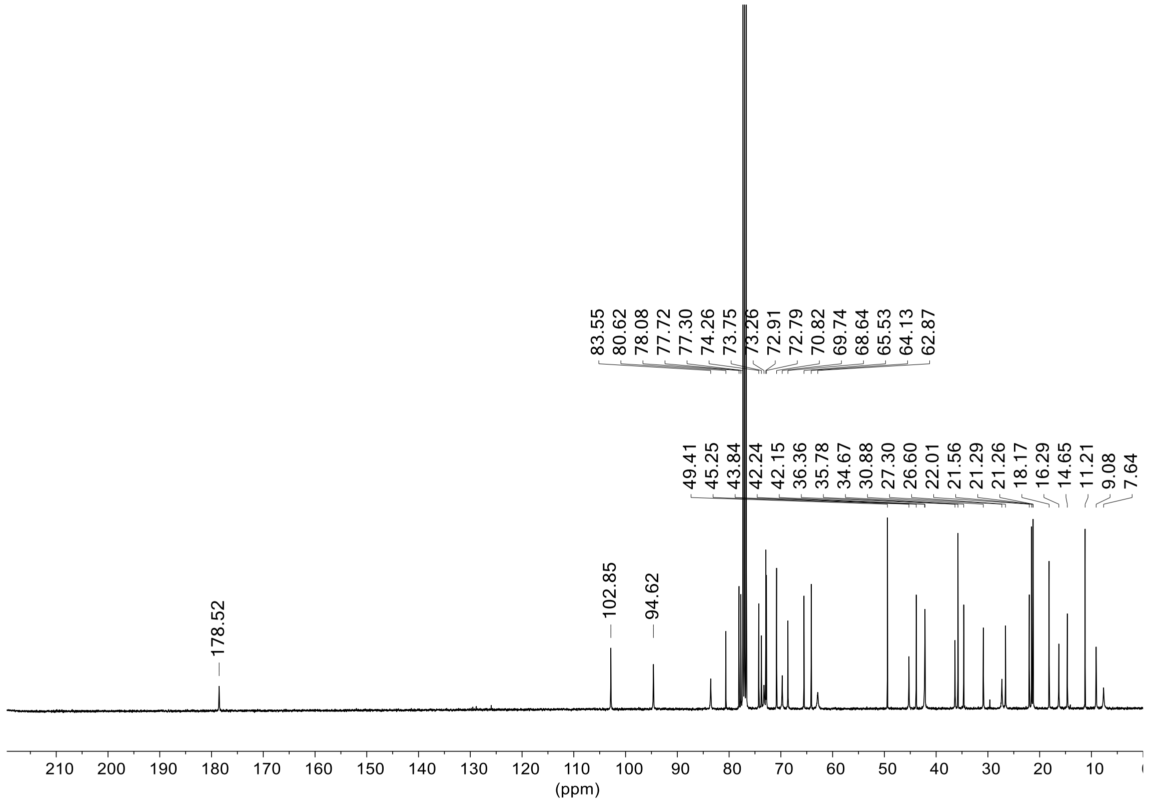


Figure S8. $^{13}C\{^1H\}$-NMR spectrum of compound **2**.

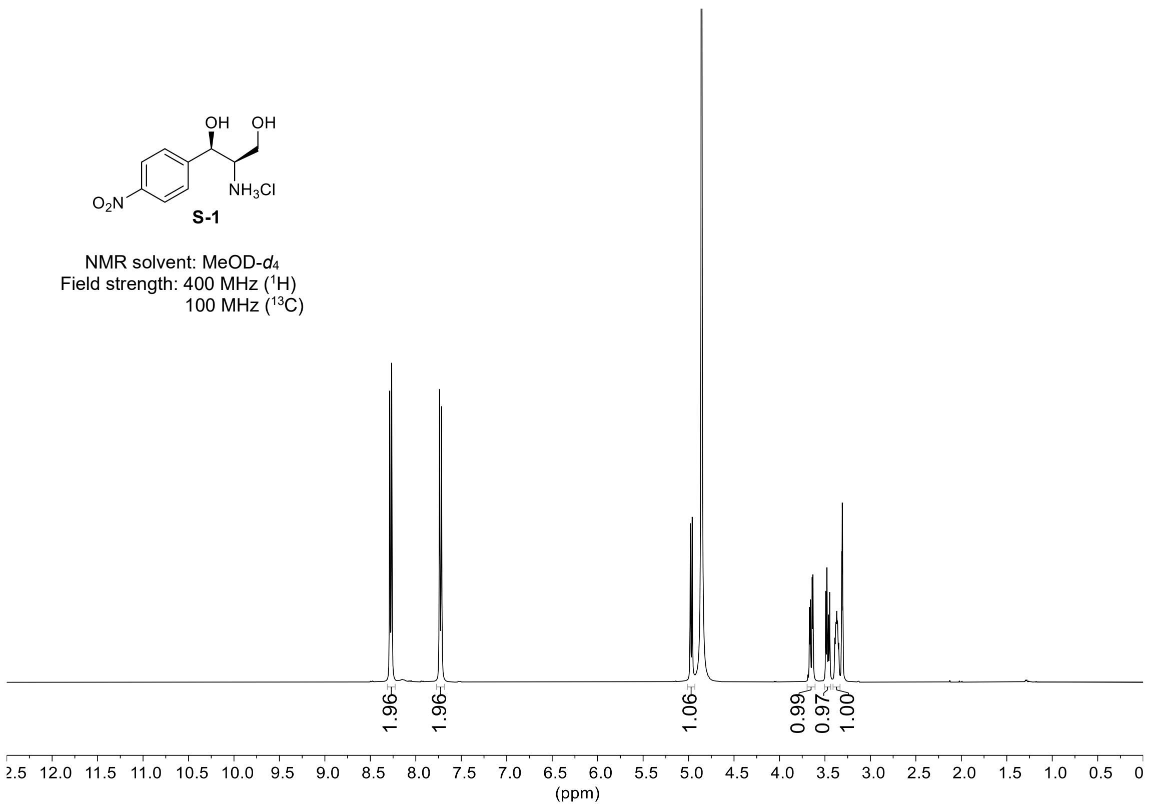


Figure S9. $^{1}$H-NMR spectrum of compound **S-1**.

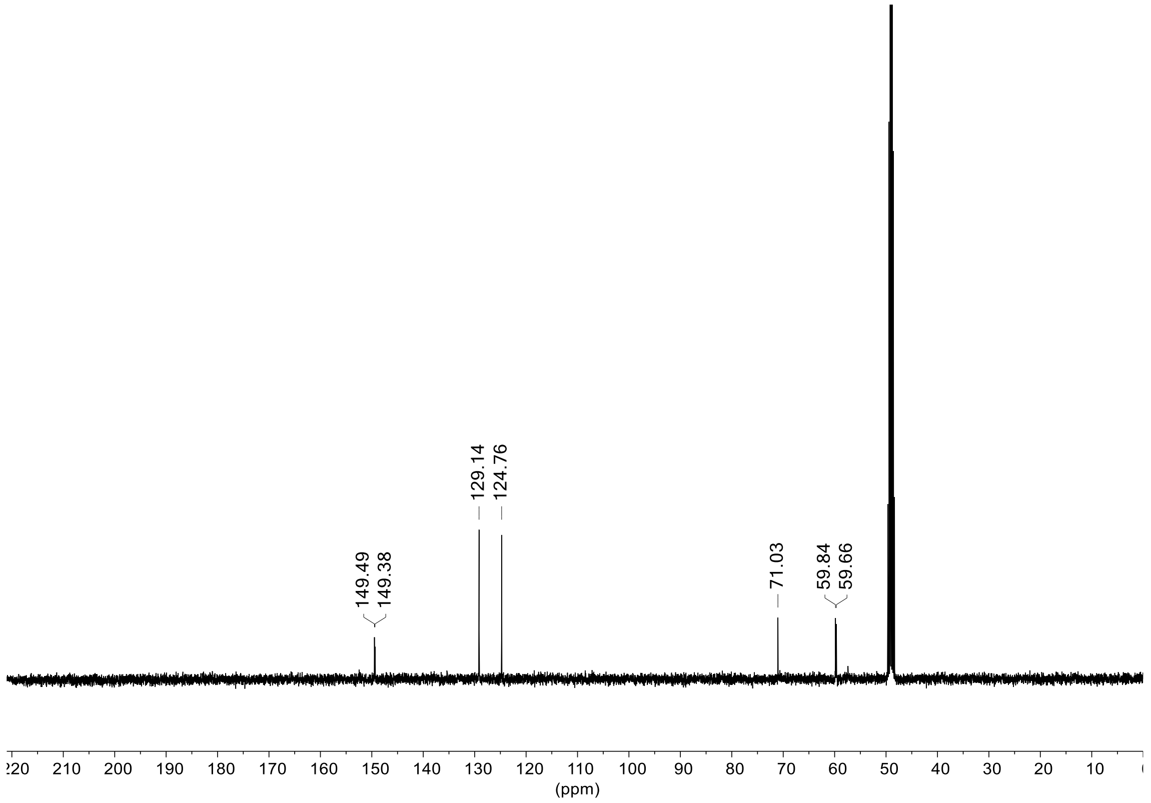


Figure S10. $^{13}$C{$^{1}$H}-NMR spectrum of compound **S-1**.

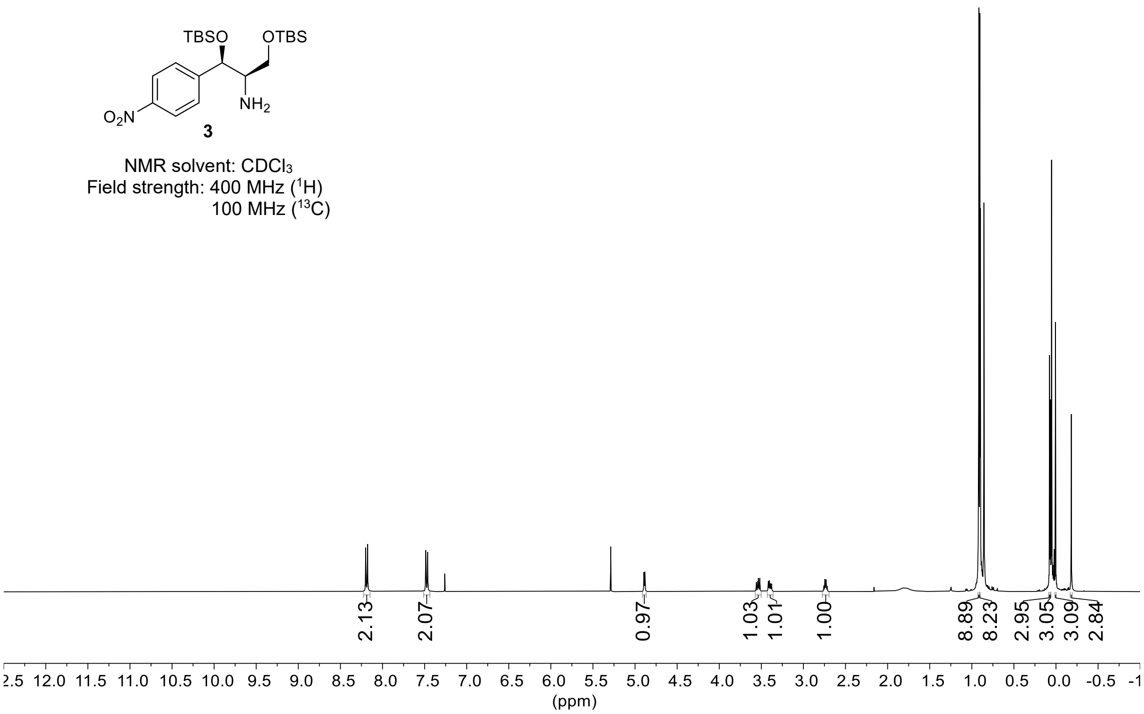


Figure S11. $^{1}$H-NMR spectrum of compound **3**.

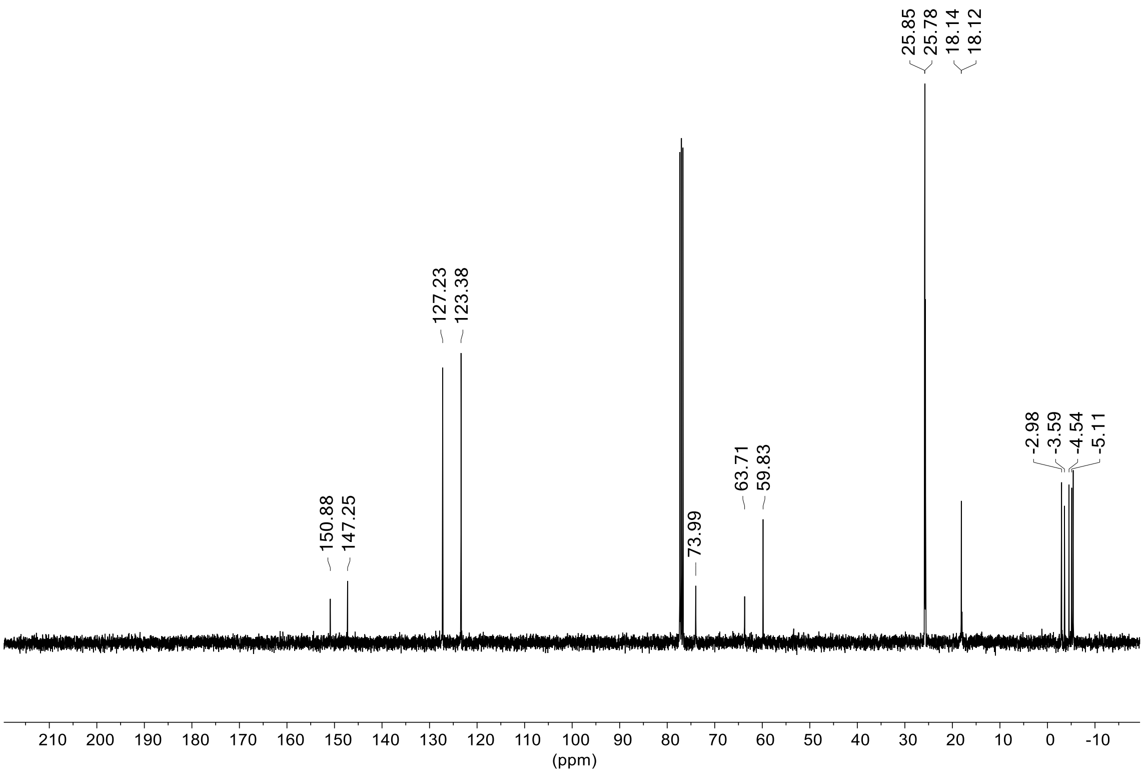


Figure S12. $^{13}$C{$^{1}$H}-NMR spectrum of compound **3**.

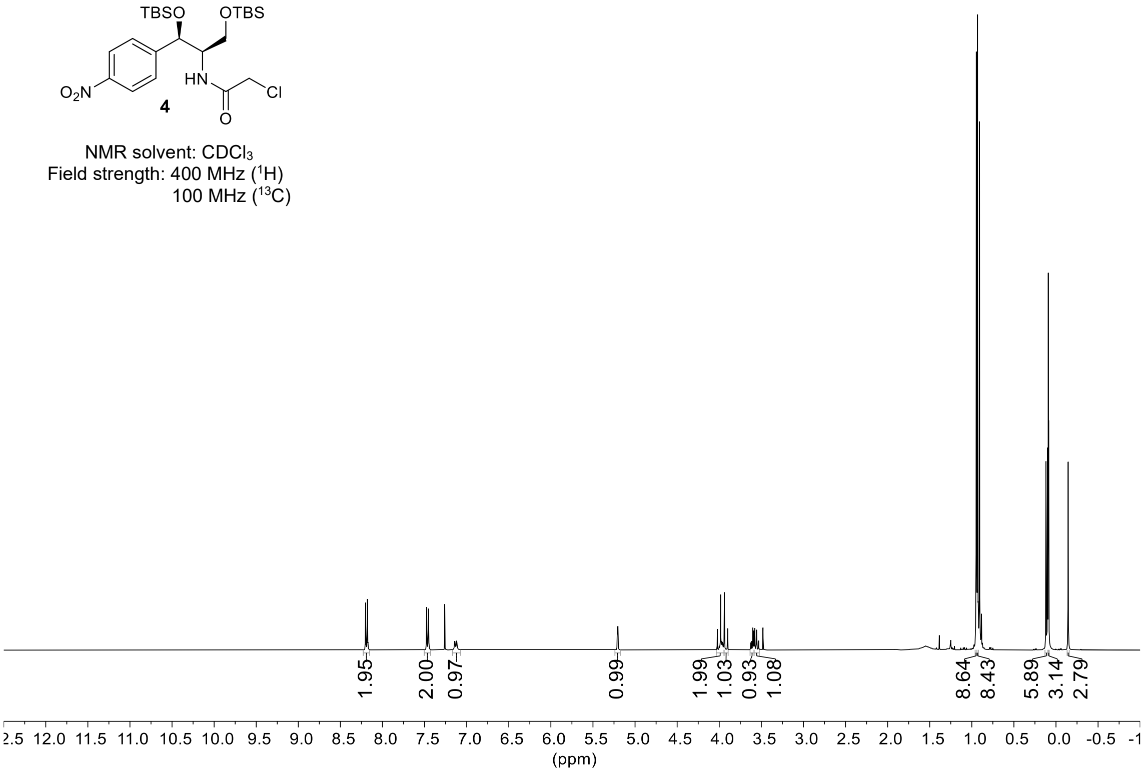


Figure S13. $^1$H-NMR spectrum of compound **4**.

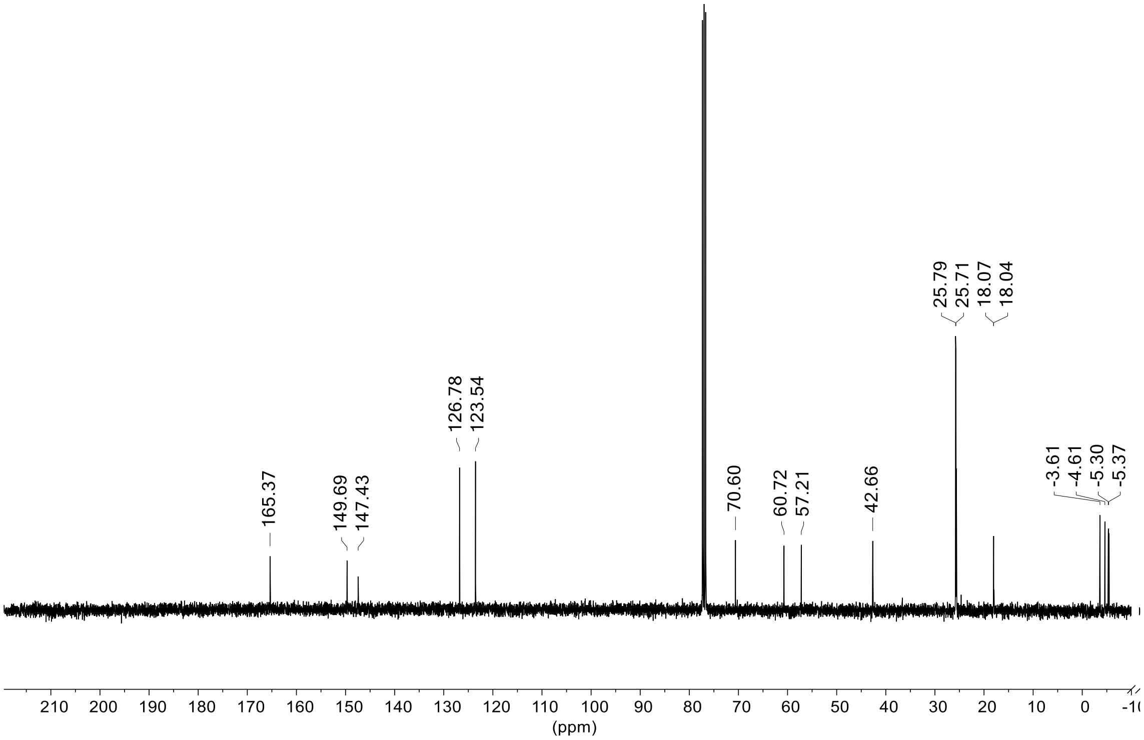


Figure S14. $^{13}$C{$^1$H}-NMR spectrum of compound **4**.

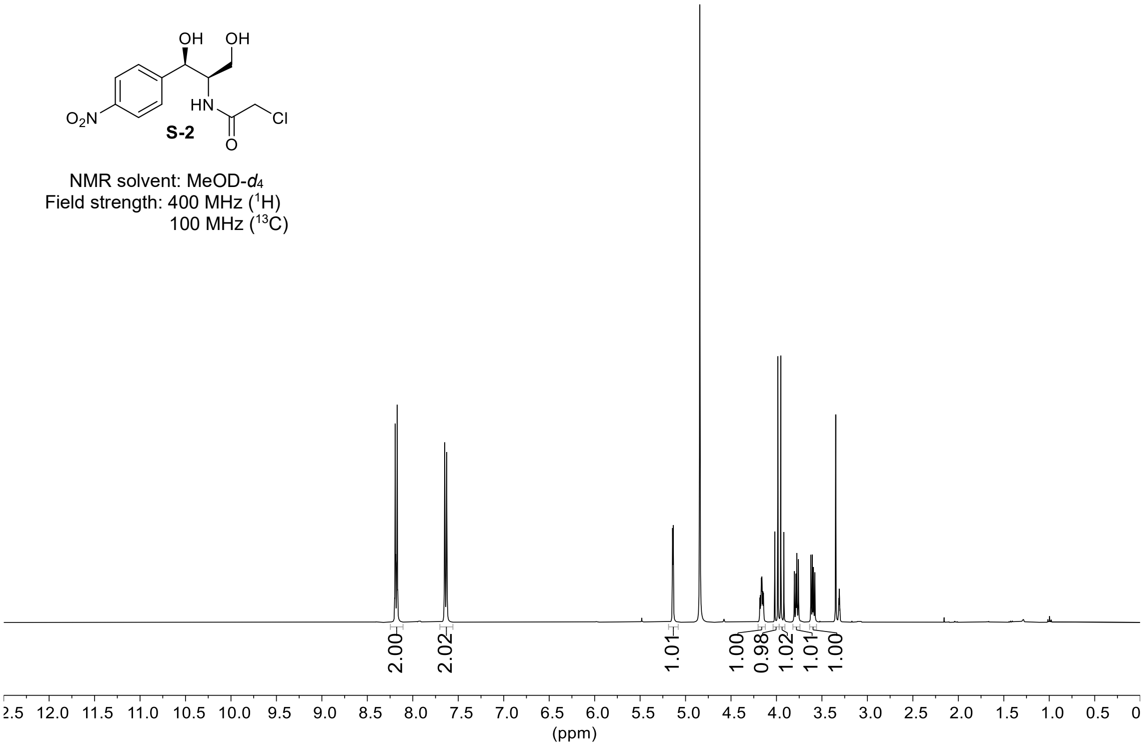


Figure S15. $^1$H-NMR spectrum of compound **S-2**.

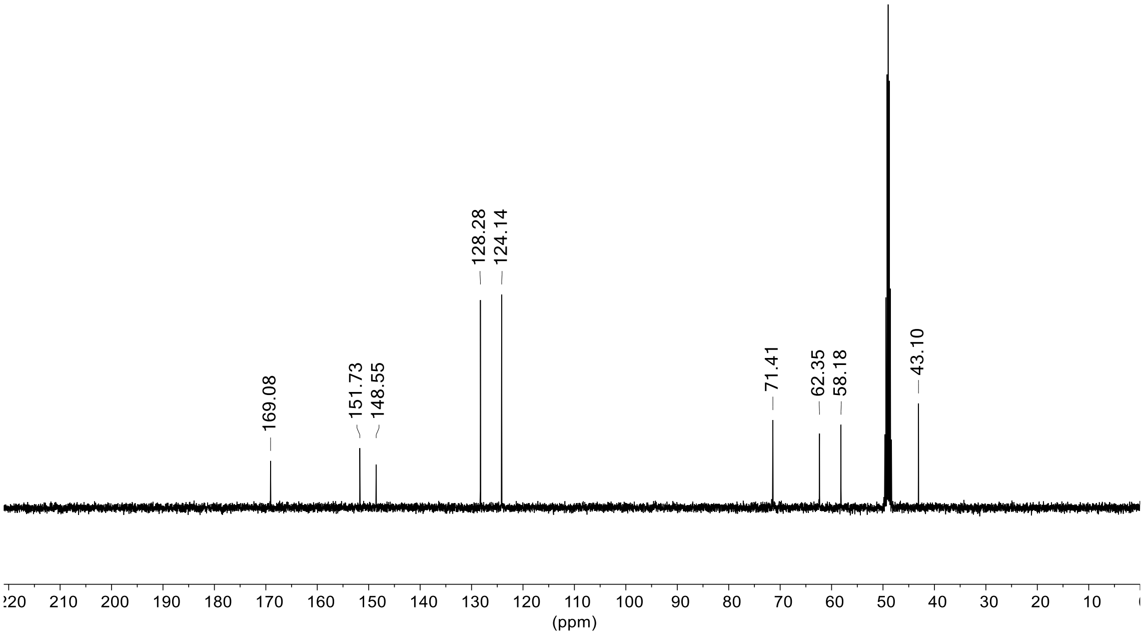


Figure S16. $^{13}$C{$^1$H}-NMR spectrum of compound **S-2**.

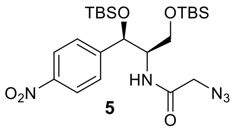

NMR solvent: $CDCl_3$
Field strength: 300 MHz ($^1H$)

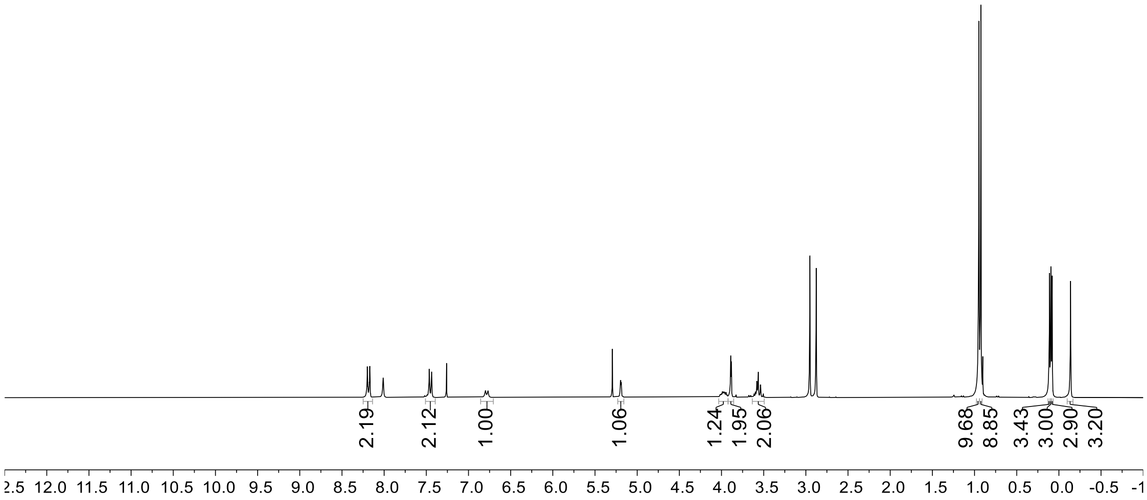


Figure S17. $^1H$-NMR spectrum of compound **5**.

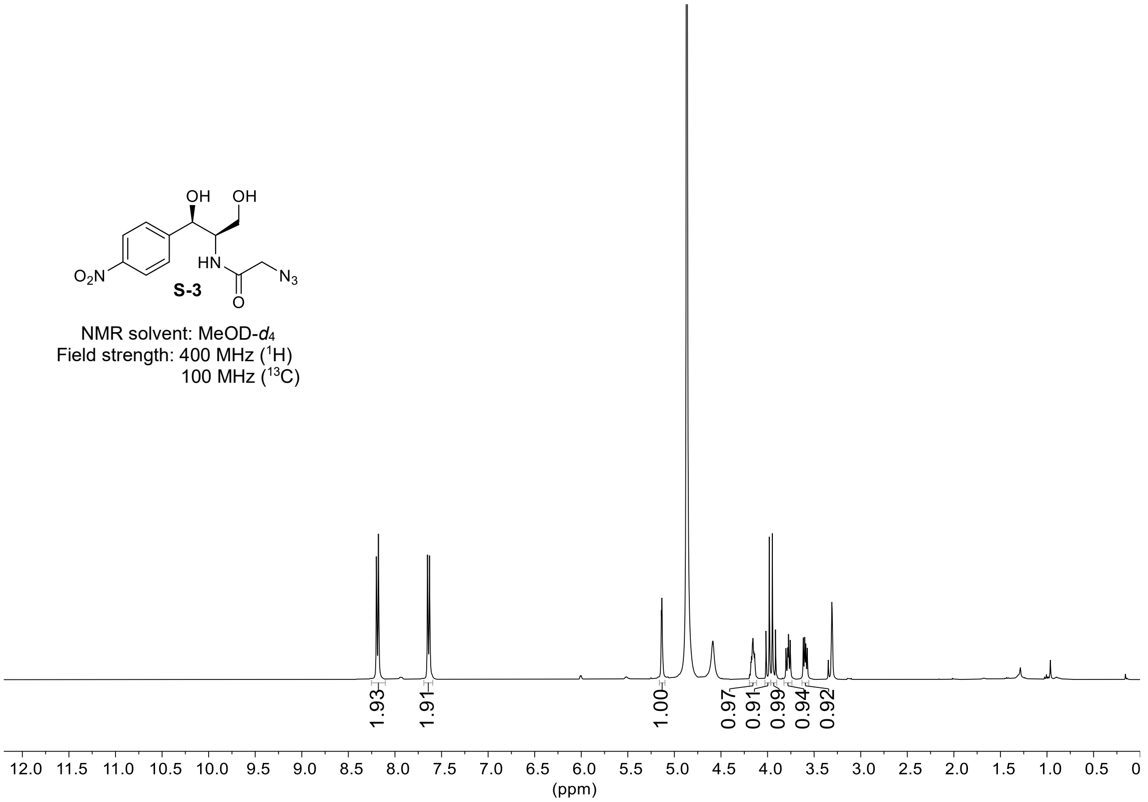


Figure S18. $^1$H-NMR spectrum of compound **S-3**.

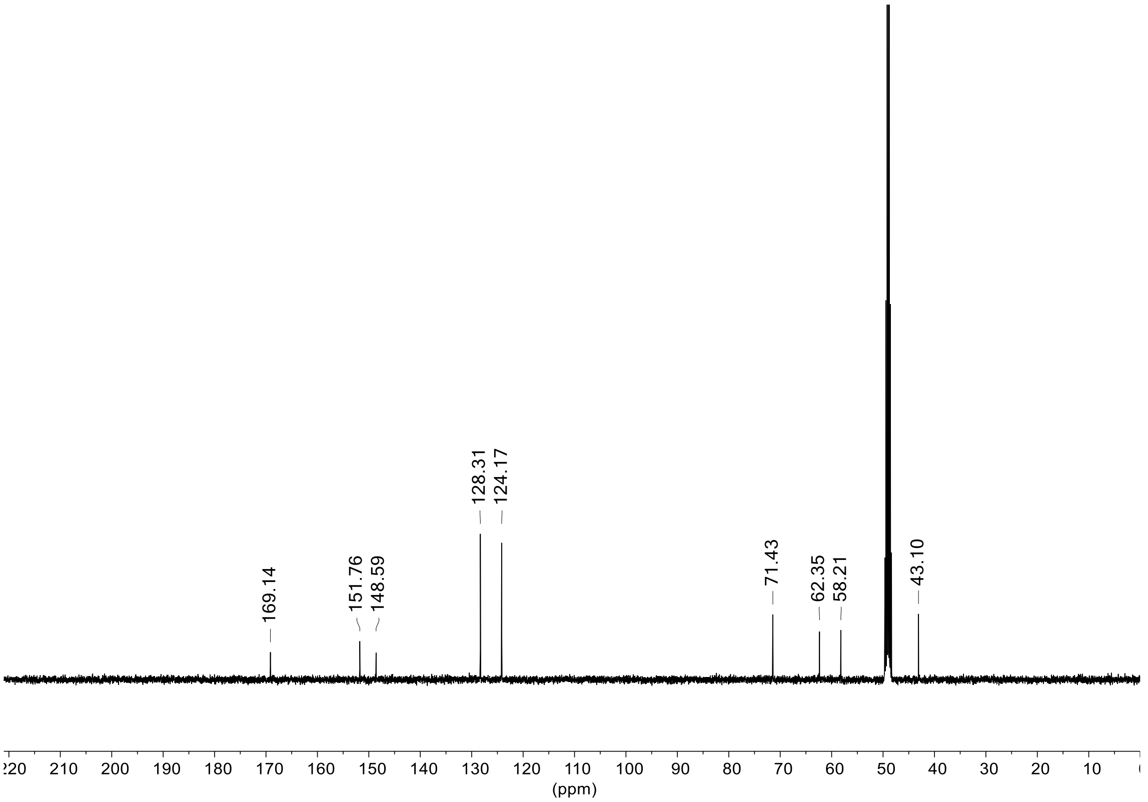


Figure S19. $^{13}$C{$^1$H}-NMR spectrum of compound **S-3**.

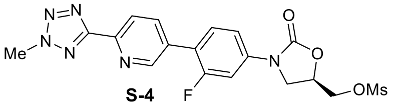

**S-4**

NMR solvent: DMSO-$d_6$
Field strength: 400 MHz ($^1$H)
100 MHz ($^{13}$C)

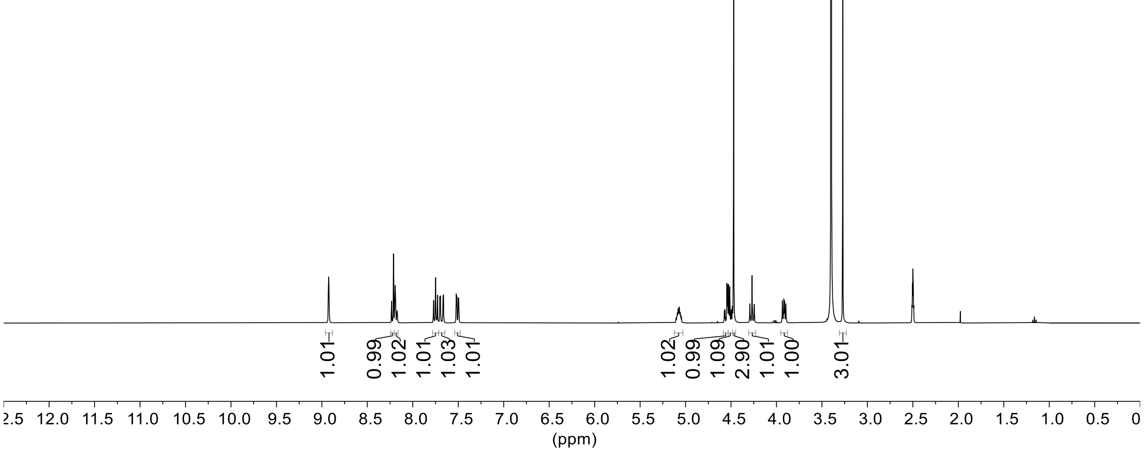


Figure S20. $^1$H-NMR spectrum of compound **S-4**.

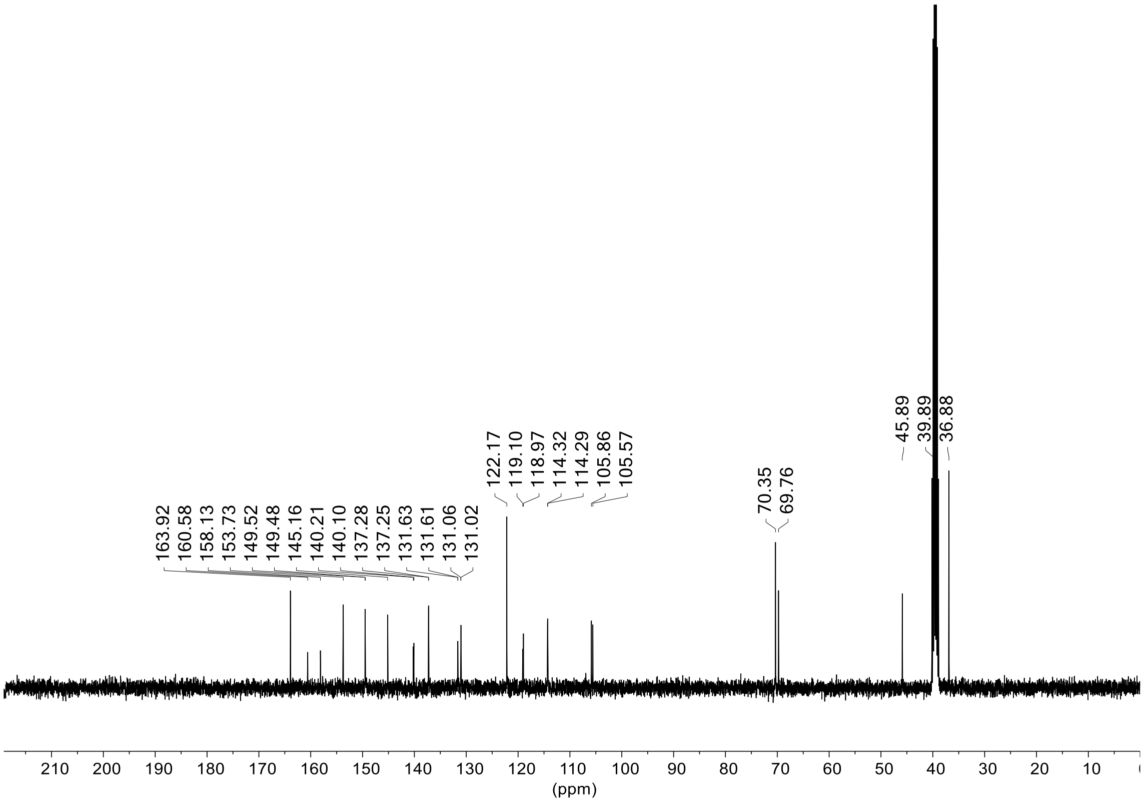


Figure S21. $^{13}$C{$^1$H}-NMR spectrum of compound **S-4**.

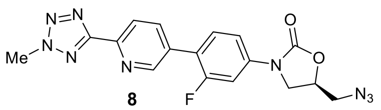


**8**

NMR solvent: DMSO-$d_6$
Field strength: 400 MHz ($^1$H)
100 MHz ($^{13}$C)

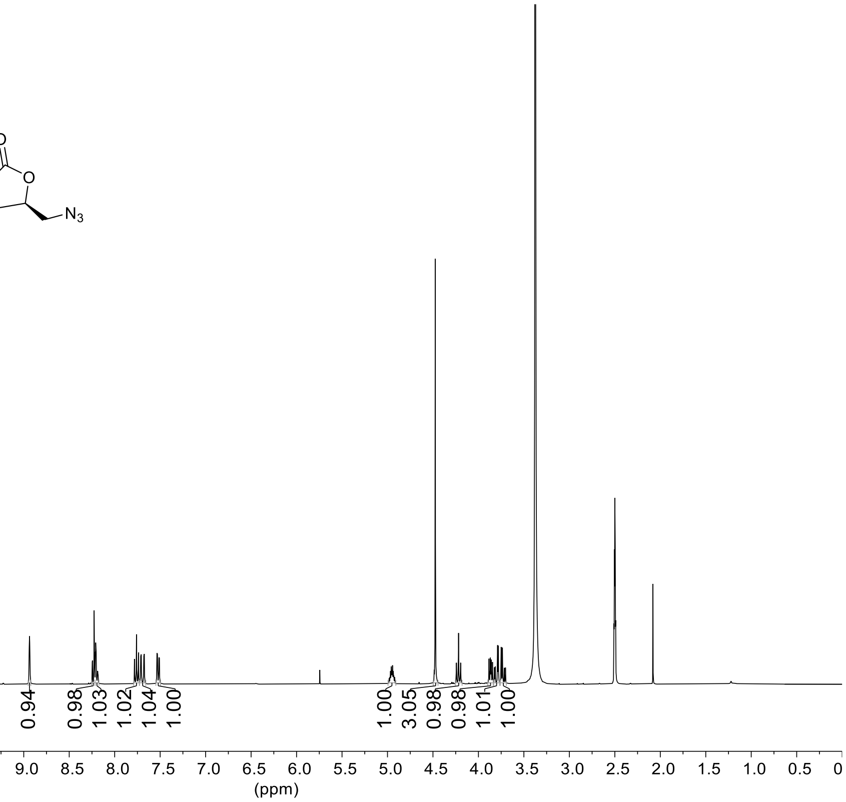


Figure S22. $^1$H-NMR spectrum of compound **8**.

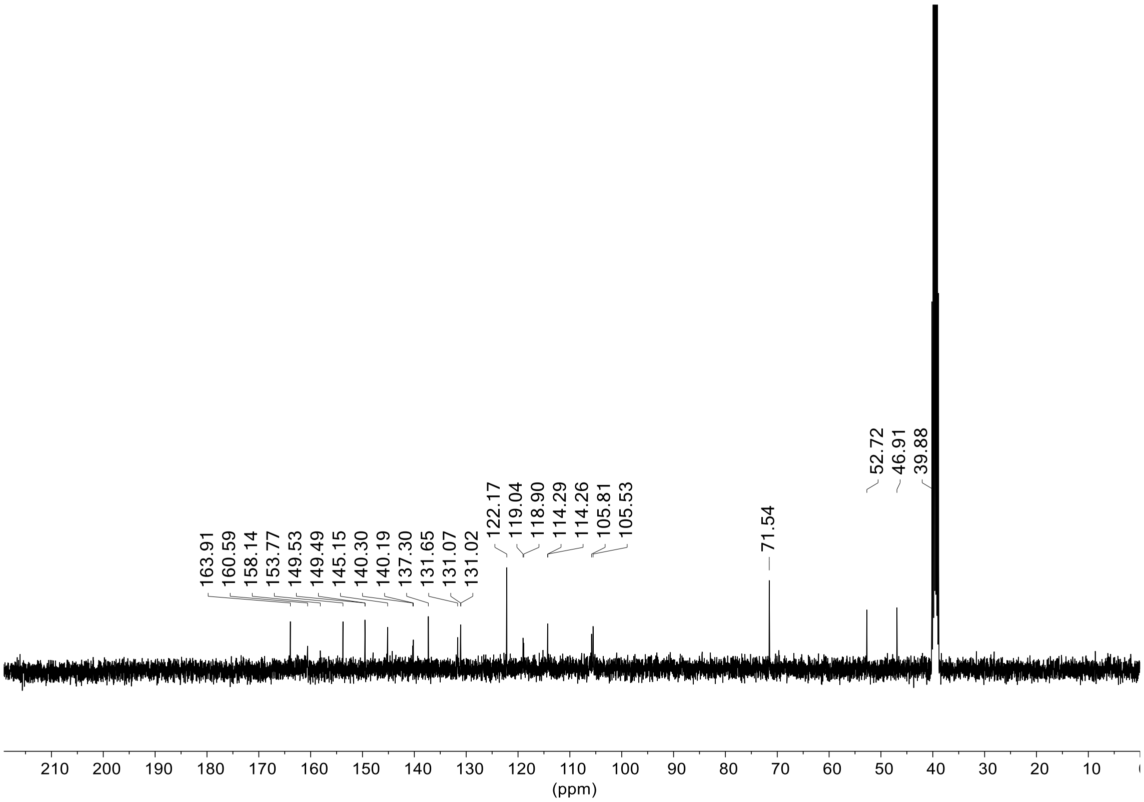


Figure S23. $^{13}$C{$^1$H}-NMR spectrum of compound **8**.

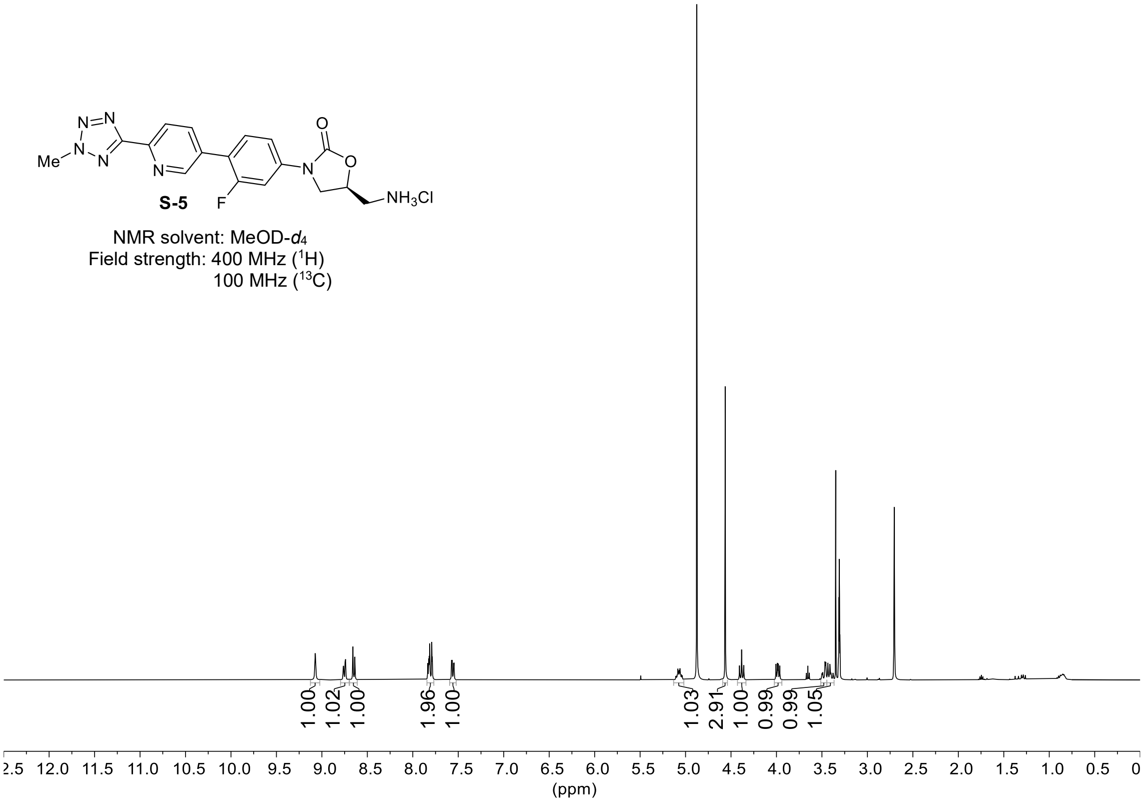

Figure S24. $^1$H-NMR spectrum of compound **S-5**.

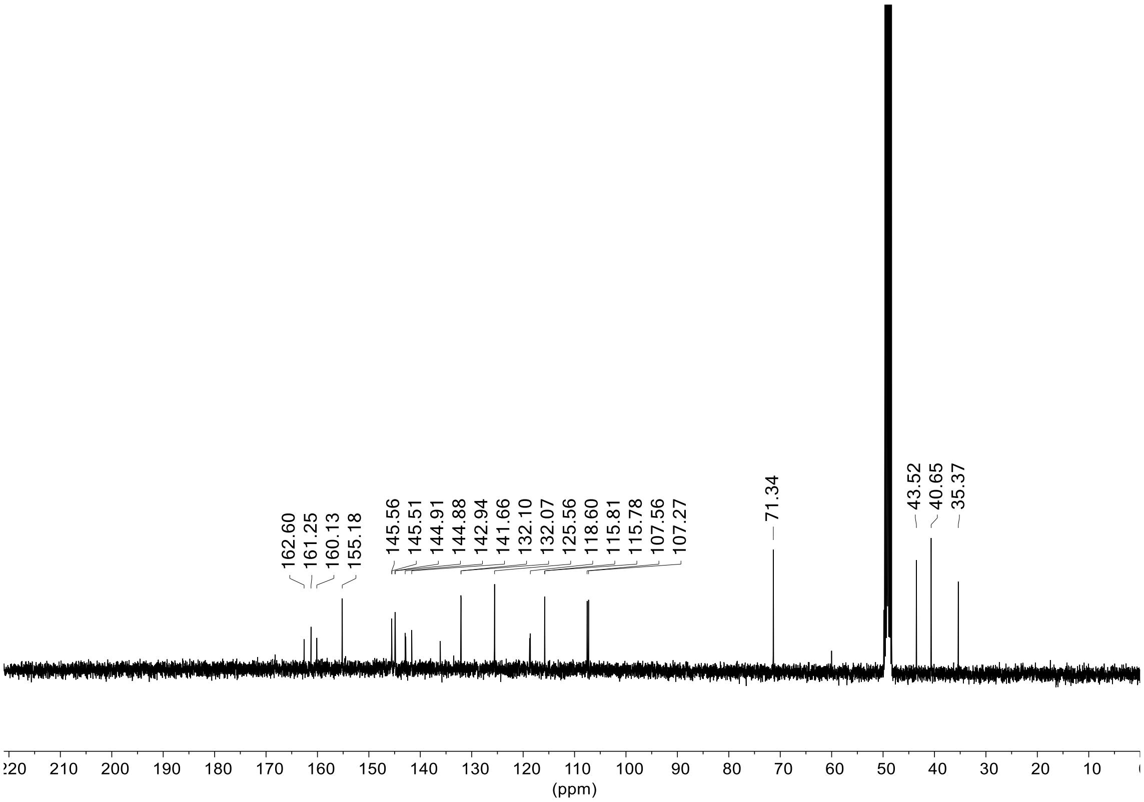

Figure S25. $^{13}$C{$^1$H}-NMR spectrum of compound **S-5**.

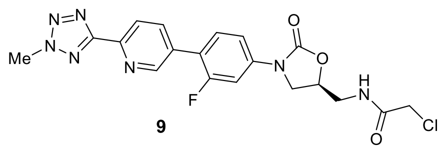


**9**

NMR solvent: DMSO-$d_6$
Field strength: 400 MHz ($^1$H)
100 MHz ($^{13}$C)

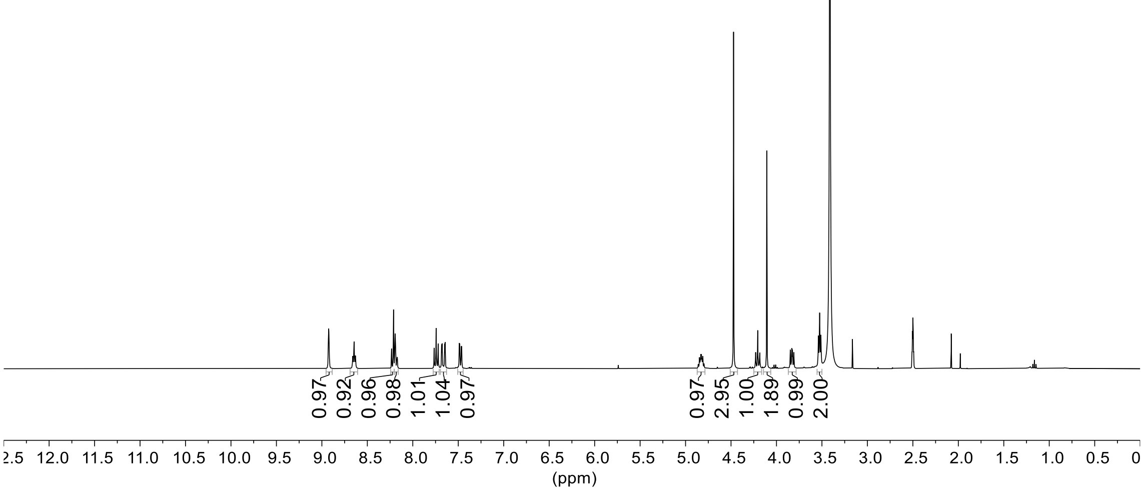


Figure S26. $^1$H-NMR spectrum of compound **9**.

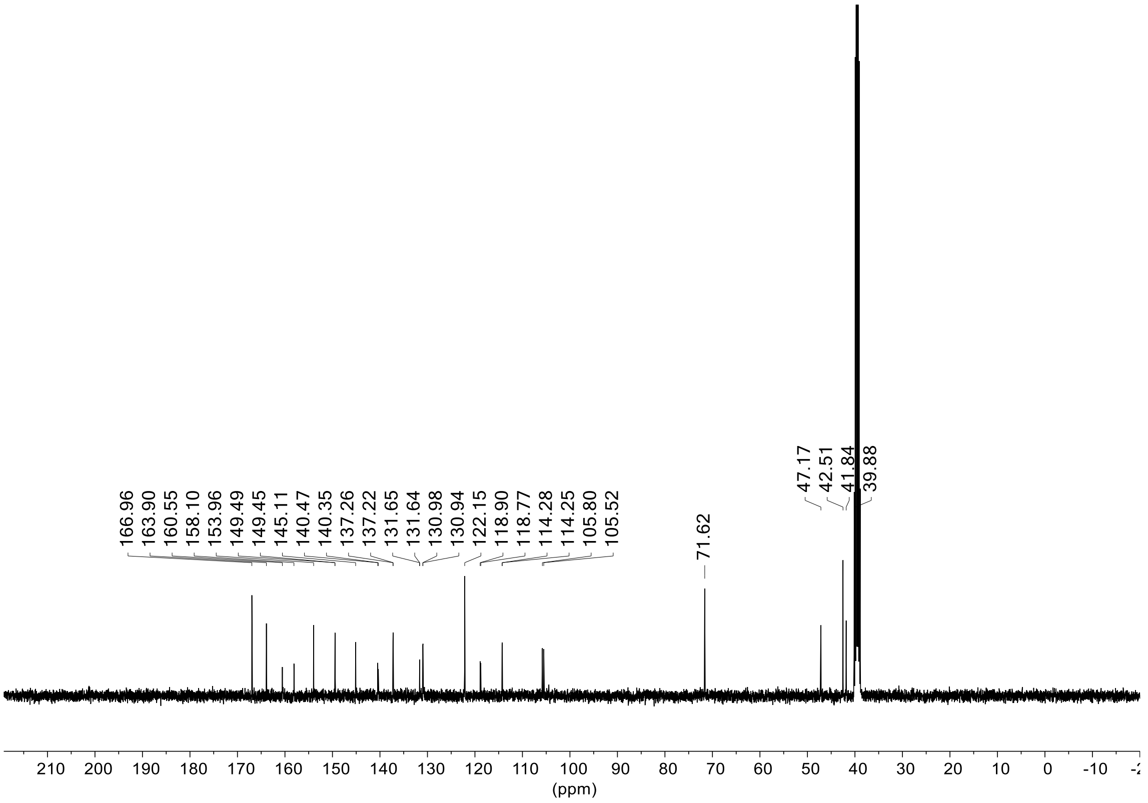


Figure S27. $^{13}$C{$^1$H}-NMR spectrum of compound **9**.

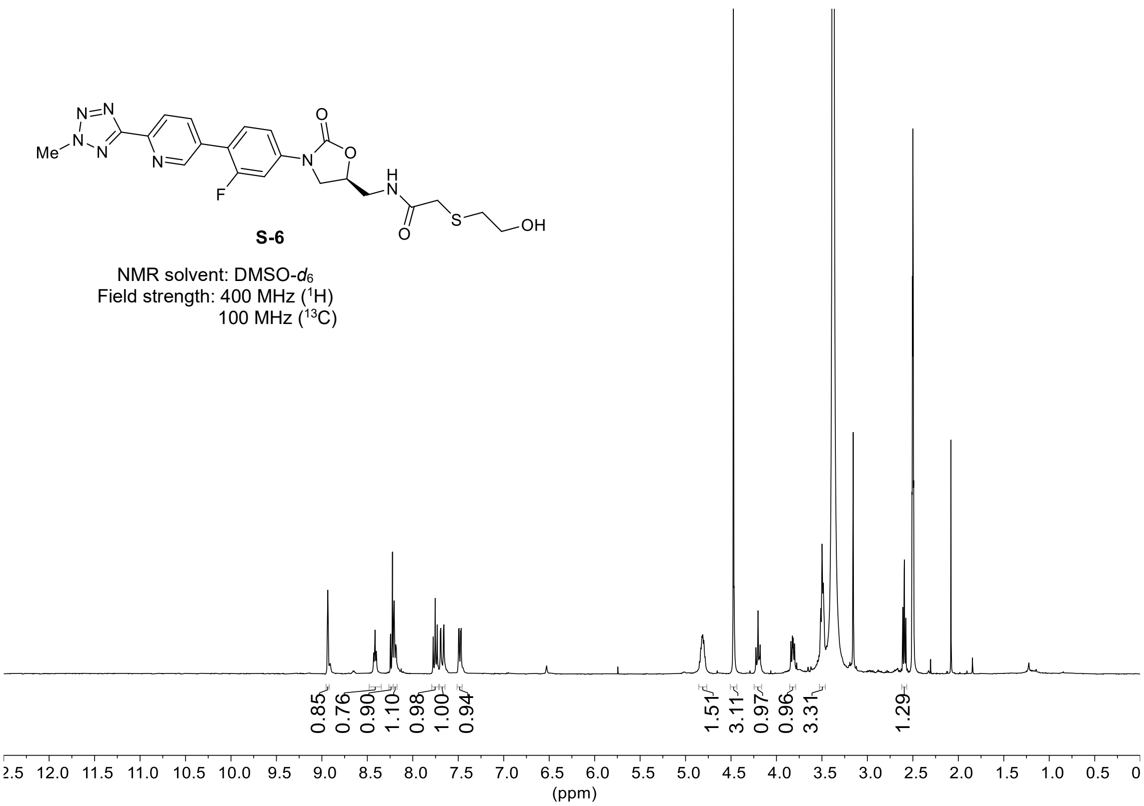


Figure S28. $^{1}$H-NMR spectrum of compound **S-6**.

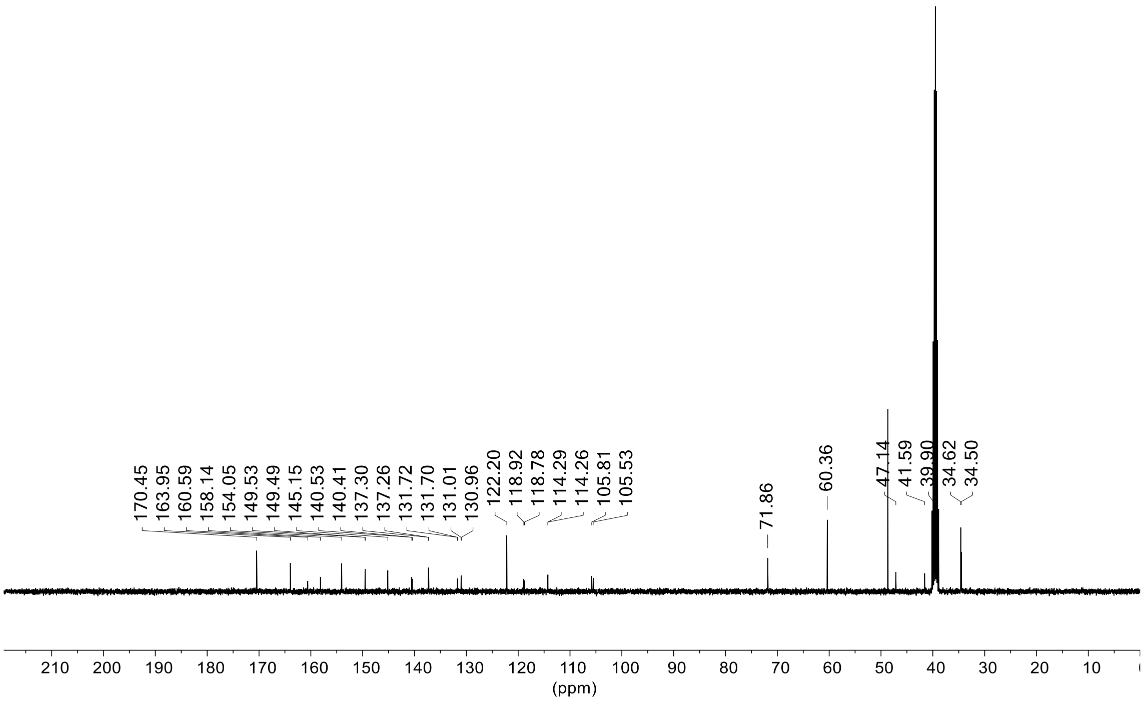


Figure S29. $^{13}$C{$^{1}$H}-NMR spectrum of compound **S-6**.

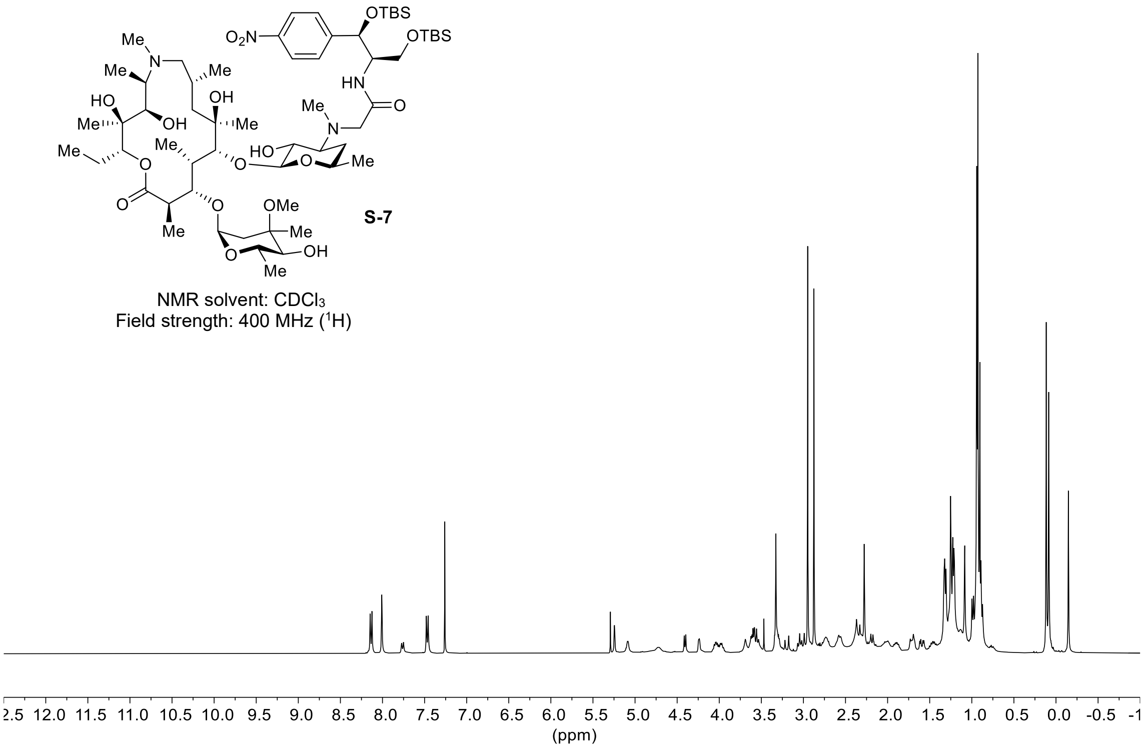


Figure S30. $^{1}$H-NMR spectrum of compound **S-7**.

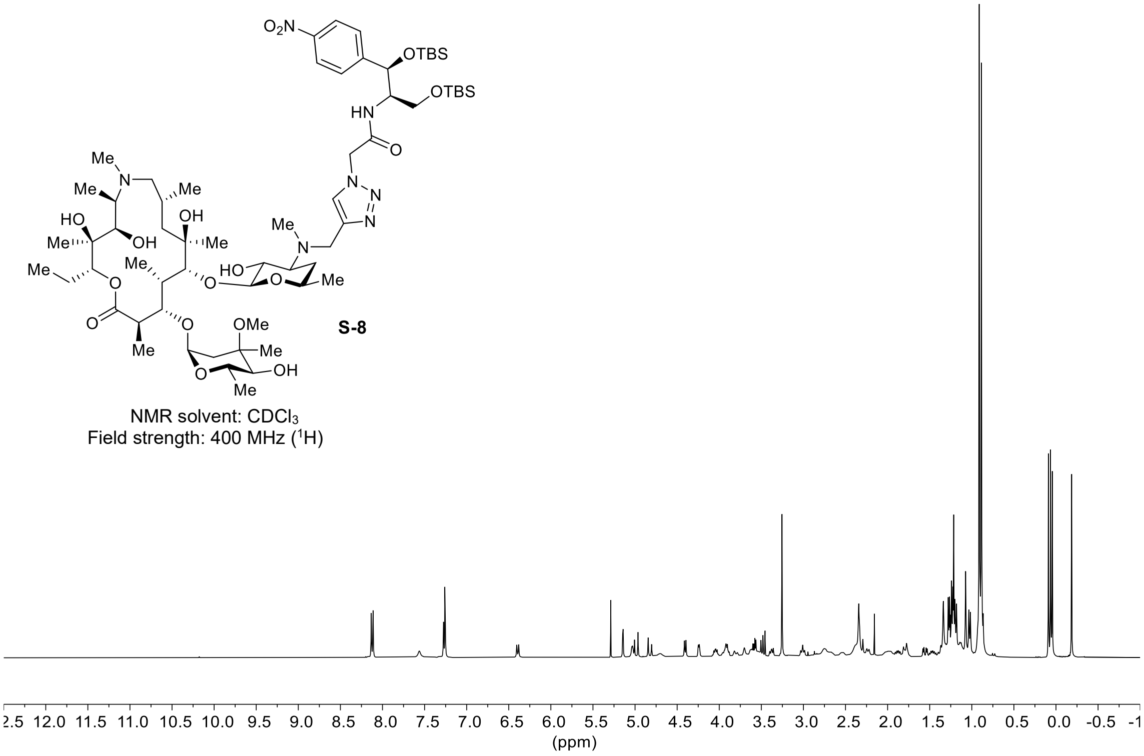


Figure S31. $^{1}$H-NMR spectrum of compound **S-8**.

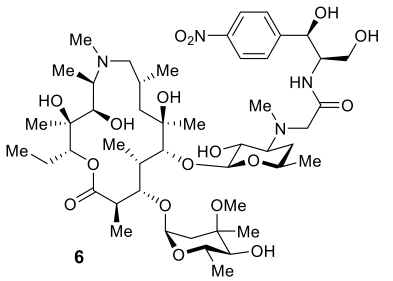


NMR solvent: MeOD-$d_4$
Field strength: 300 MHz ($^1$H)
100 MHz ($^{13}$C)

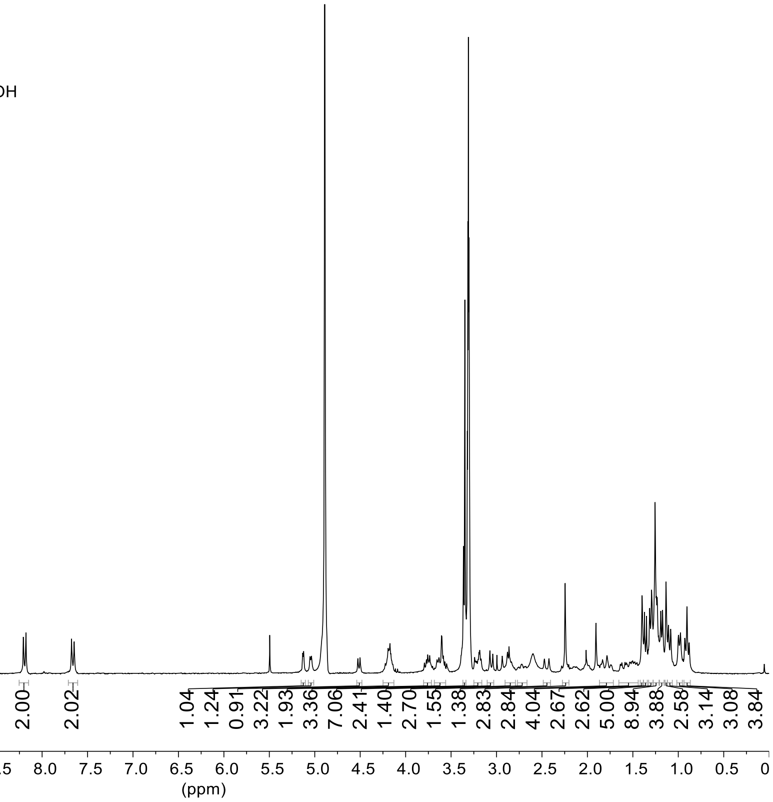


Figure S32. $^1$H-NMR spectrum of compound **6**.

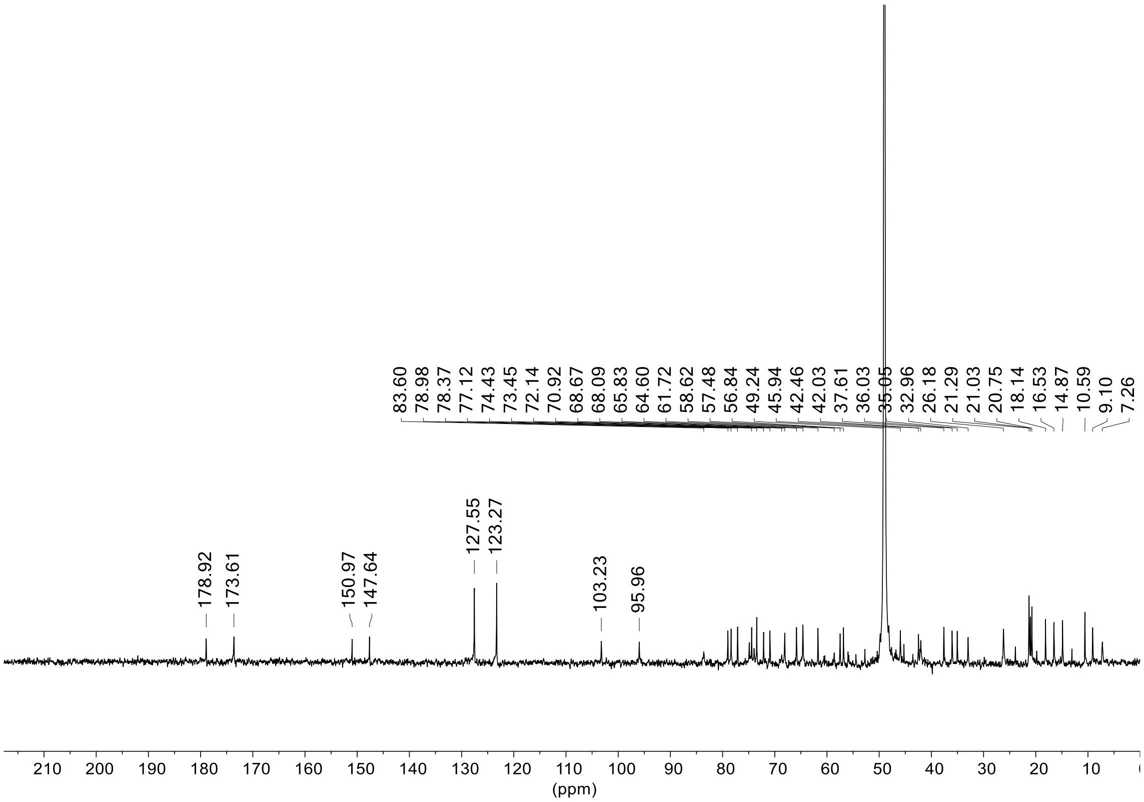


Figure S33. $^{13}$C{$^1$H}-NMR spectrum of compound **6**.

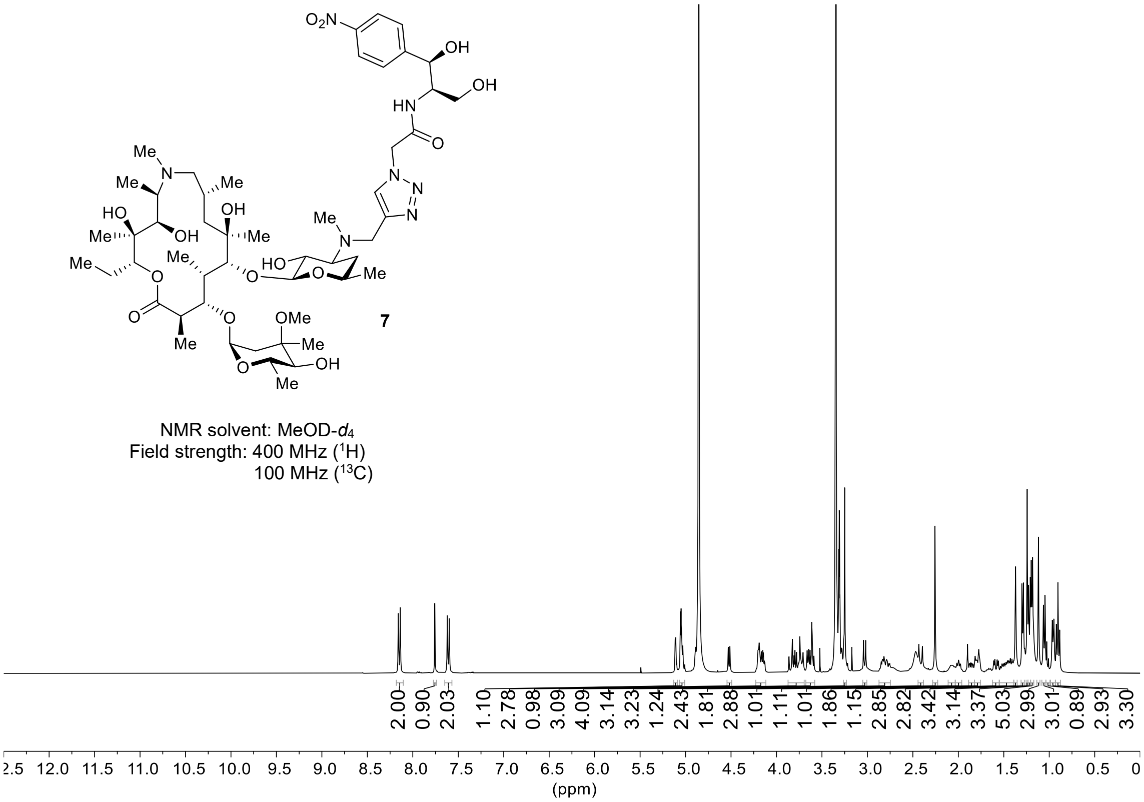


Figure S34. $^1$H-NMR spectrum of compound **7**.

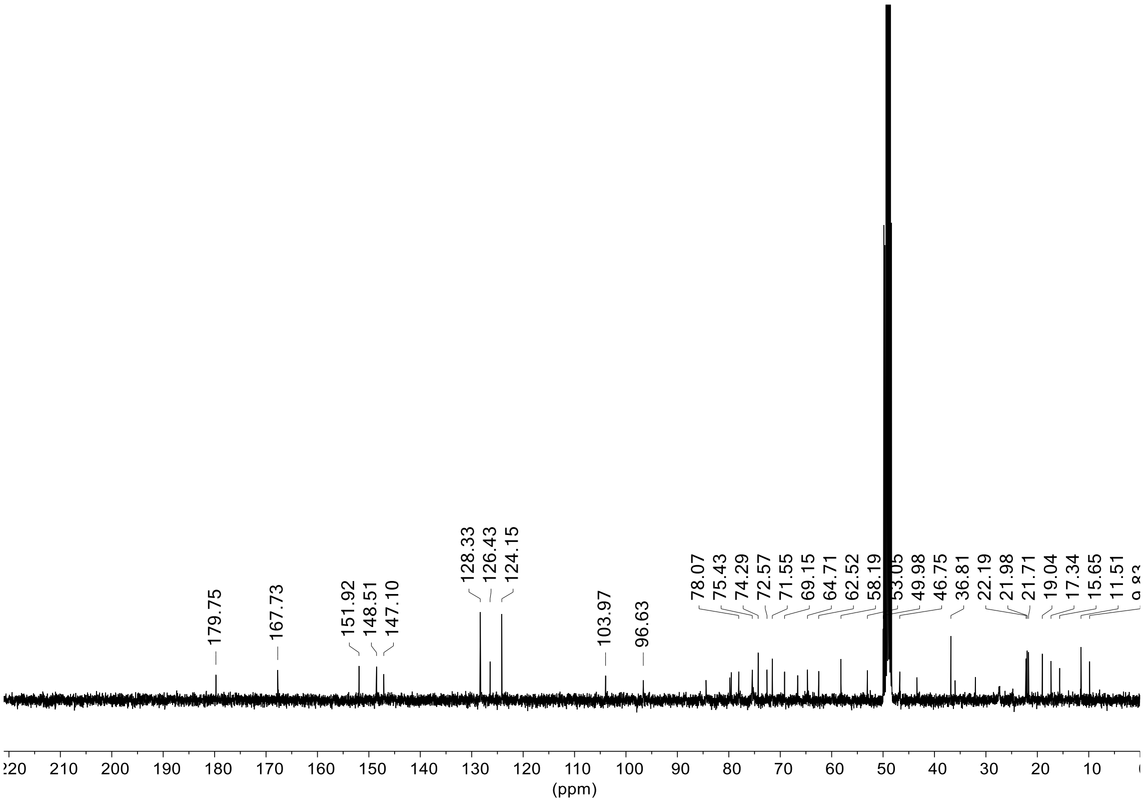


Figure S35. $^{13}$C{$^1$H}-NMR spectrum of compound **7**.

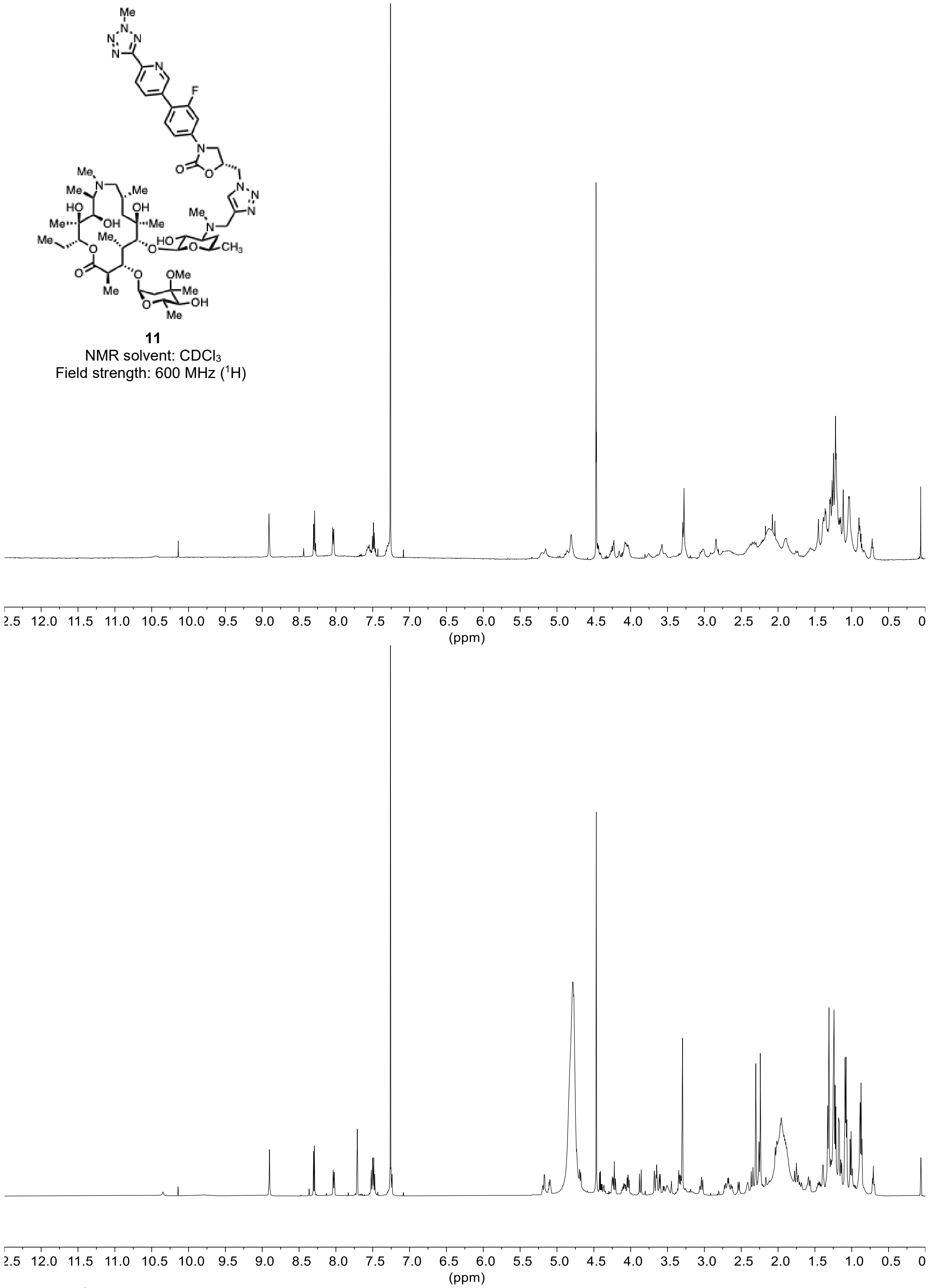


Figure S36. $^{1}H$-NMR spectra (600.1 MHz, $CDCl_3$) of **11** before (top) and after (bottom) addition of one drop of 50% (v/v) $NH_4OH_{aq}$.

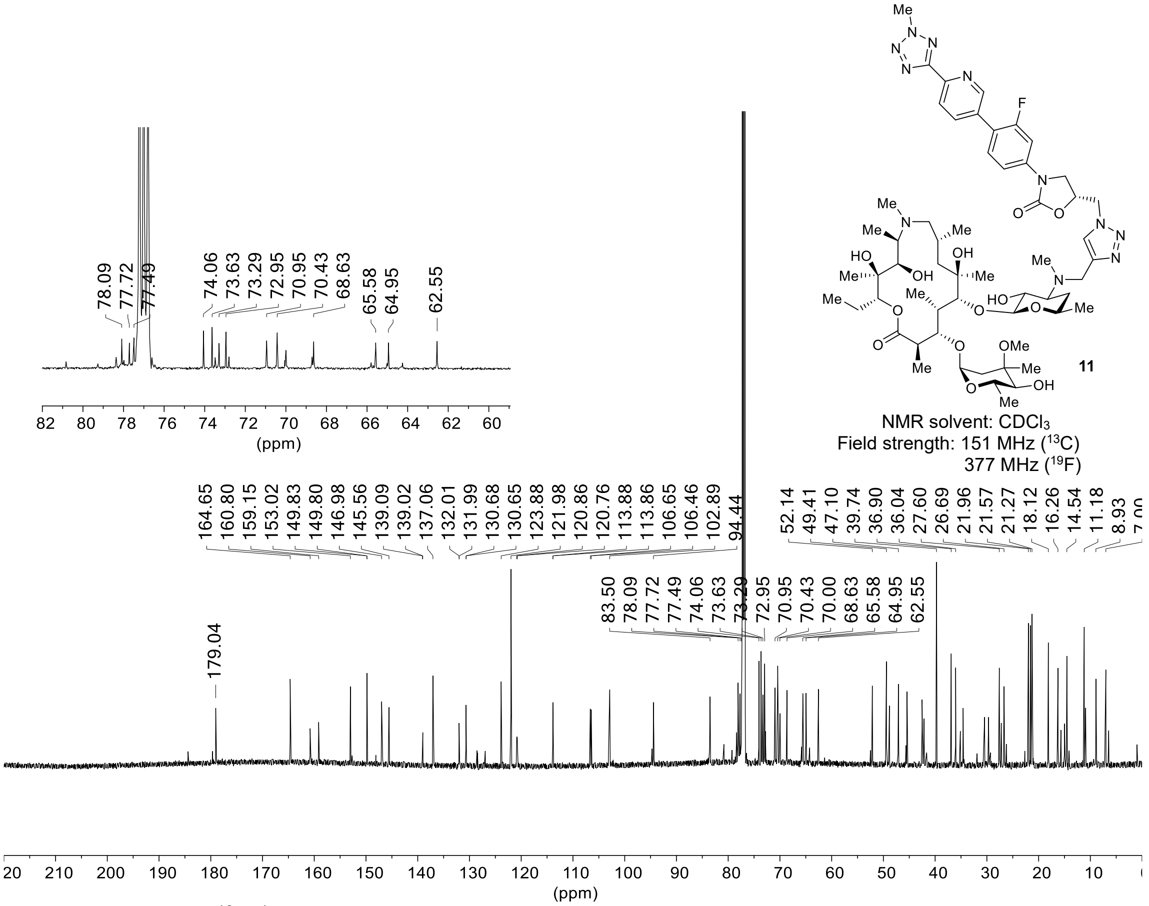


Figure S37. $^{13}C\{^1H\}$-NMR spectrum (150.9 MHz; CDCl$_3$ + drop of 50% (v/v) NH$_4$OH$_{aq}$) of **11**.

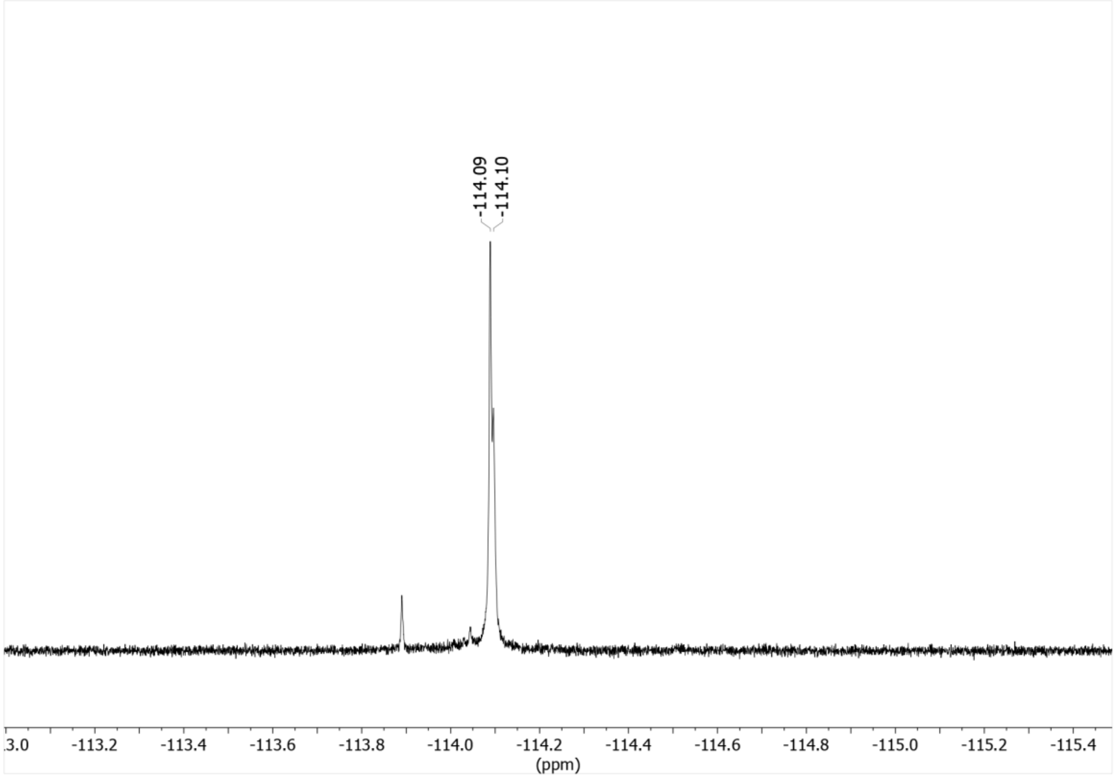


Figure S38. $^{19}F\{^1H\}$-NMR spectrum (376.5 MHz; CDCl$_3$ + drop of 50% (v/v) NH$_4$OH$_{aq}$) of **11**.

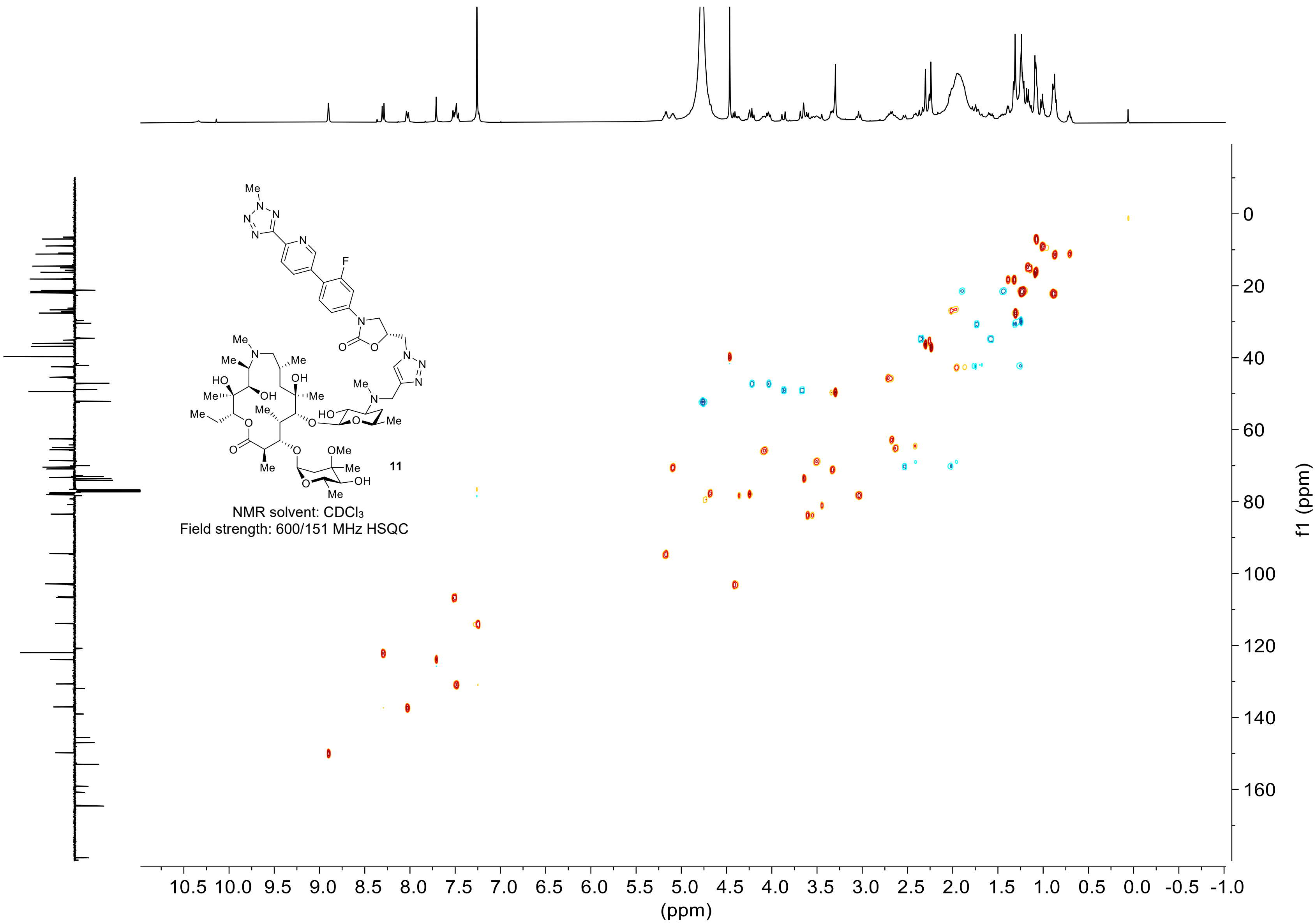


Figure S39. $^{1}$H–$^{13}$C-HSQC NMR spectrum (600.1/150.9 MHz; $CDCl_3$ + drop of 50% (v/v) $NH_4OH_{aq}$) of **11**.

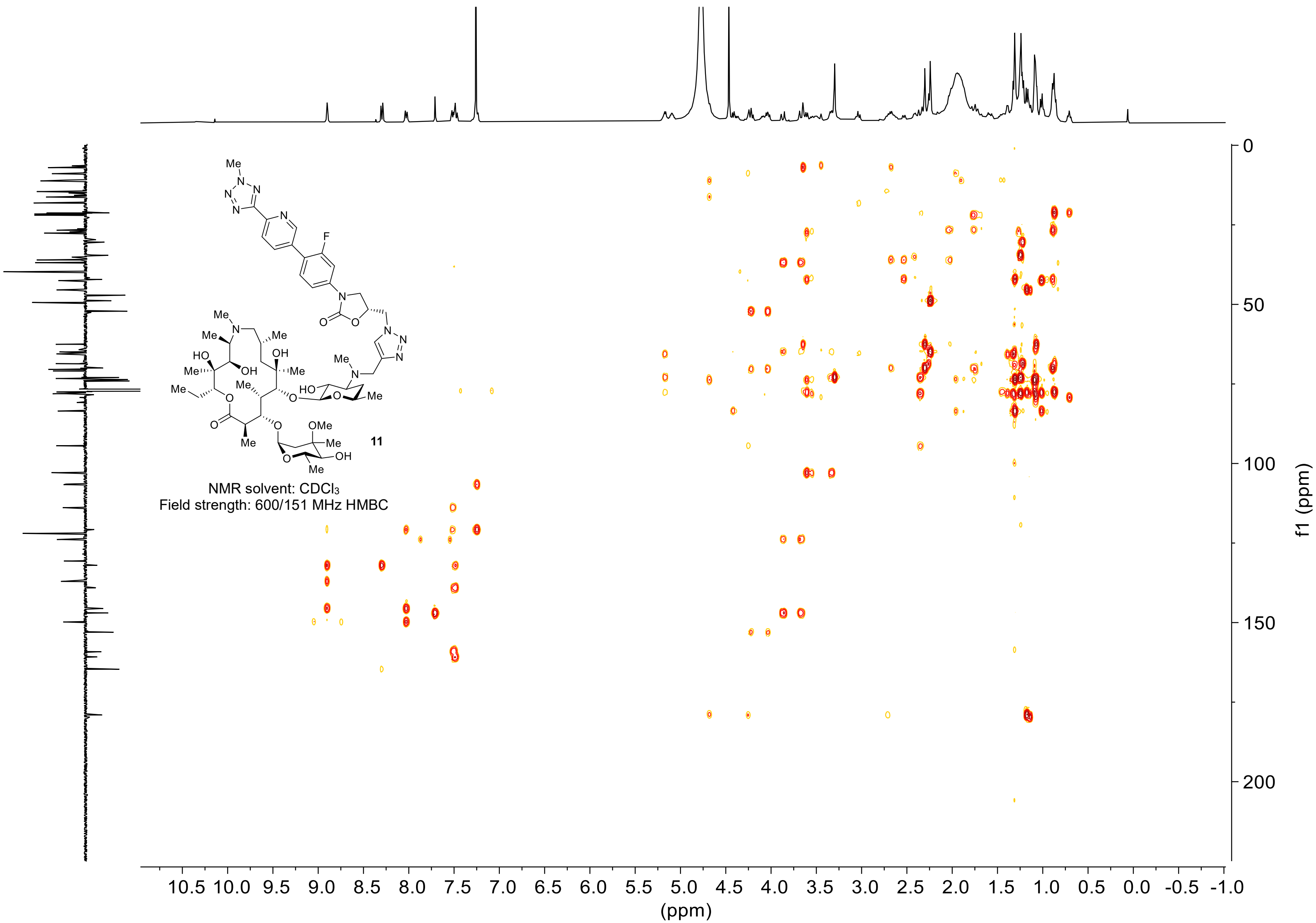


Figure S40. $^{1}H$–$^{13}C$-HMBC NMR spectrum (600.1/150.9 MHz; $CDCl_3$ + drop of 50% (v/v) $NH_4OH_{aq}$) of **11**.

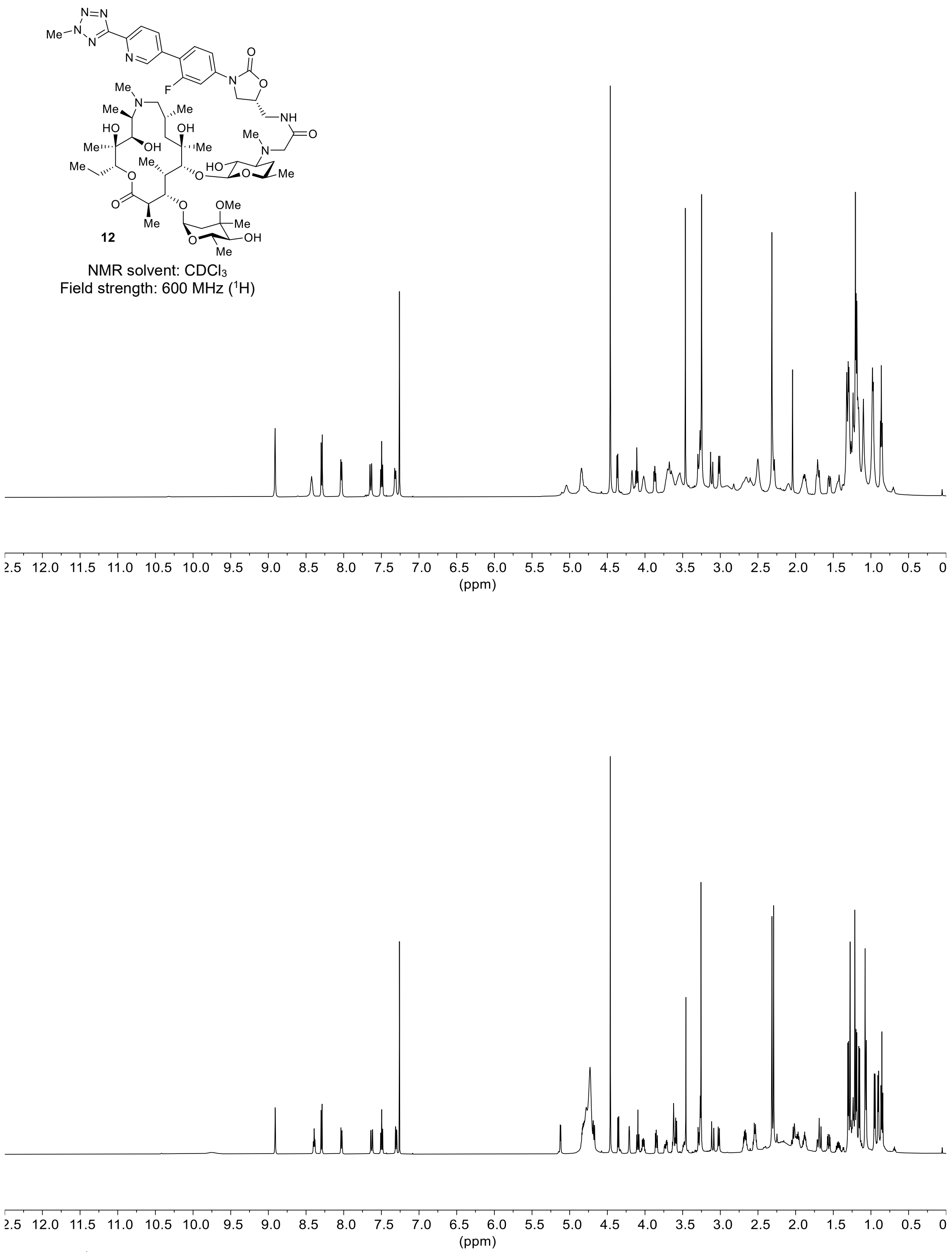


Figure S41. $^{1}$H-NMR spectra (600.1 MHz, $CDCl_3$) of **12** before (top) and after (bottom) addition of one drop of 50% (v/v) $NH_4OH_{aq}$.

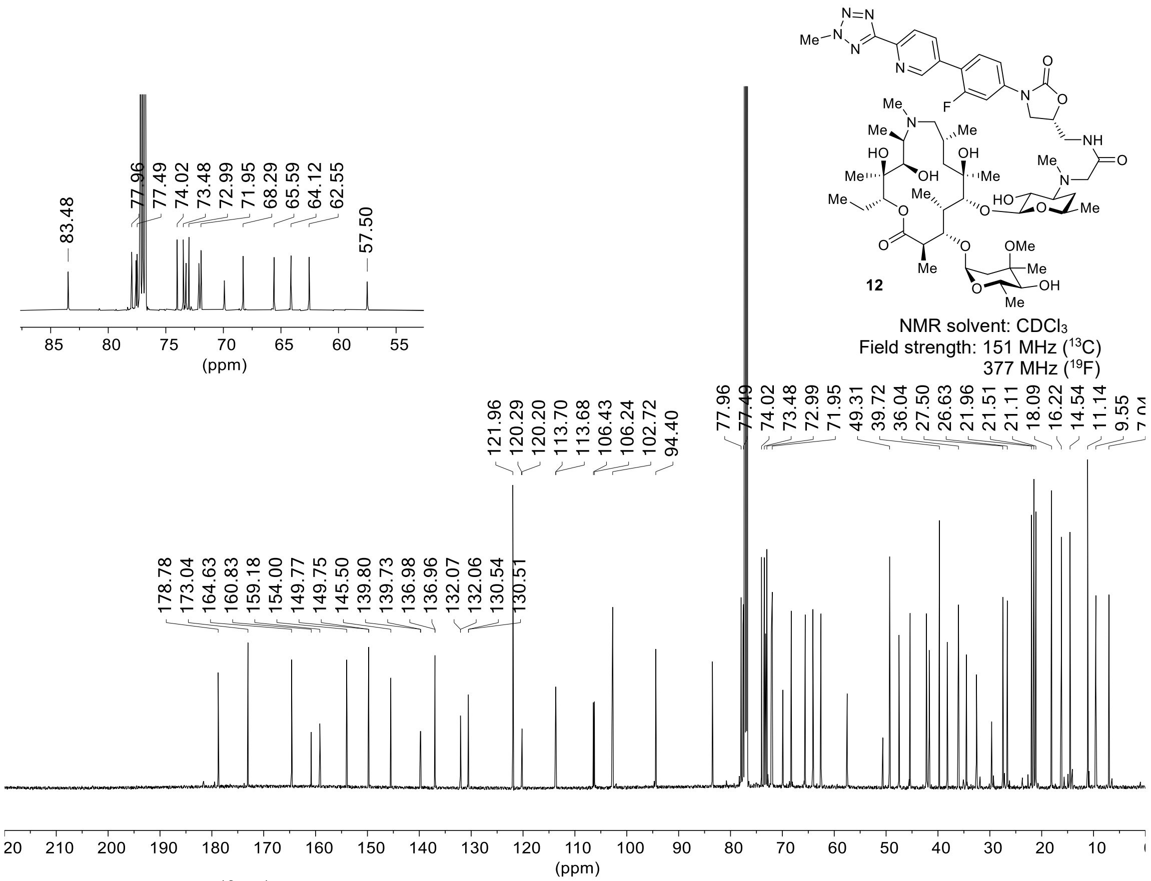


Figure S42. $^{13}C\{^{1}H\}$-NMR spectrum (150.9 MHz; $CDCl_3$ + drop of 50% (v/v) $NH_4OH_{aq}$) of **12**.

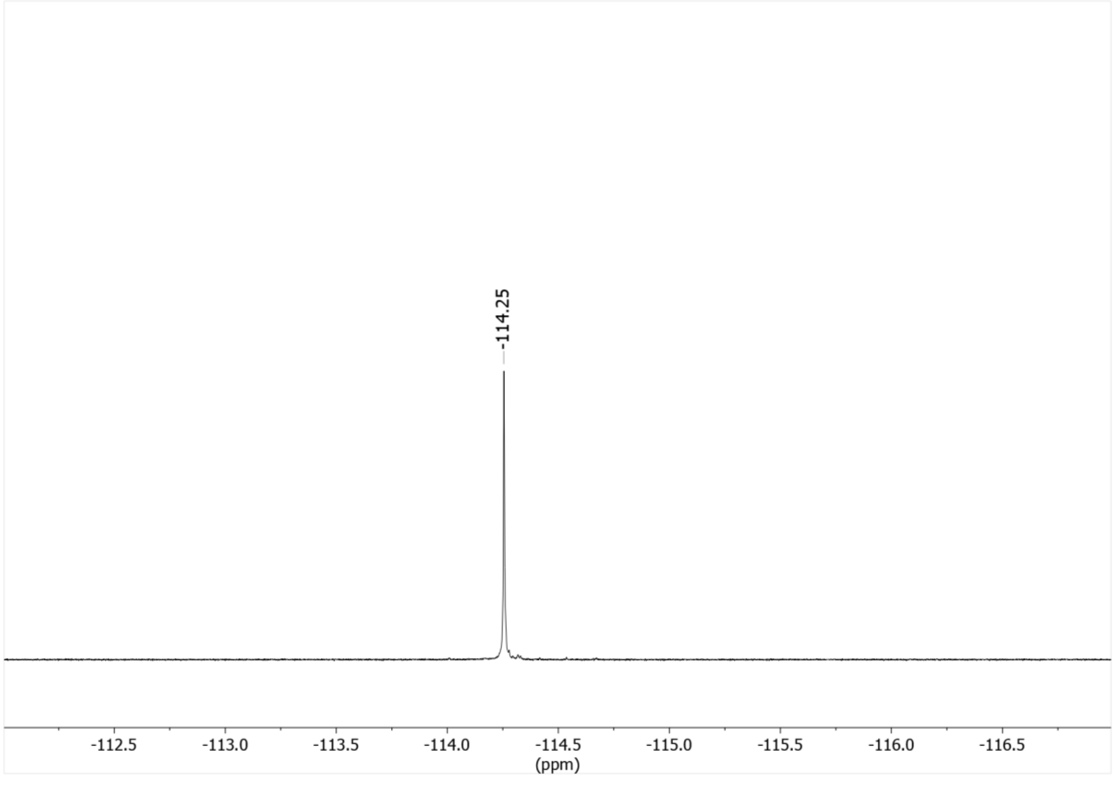


Figure S43. $^{19}F\{^{1}H\}$-NMR spectrum (376.5 MHz; $CDCl_3$ + drop of 50% (v/v) $NH_4OH_{aq}$) of **12**.

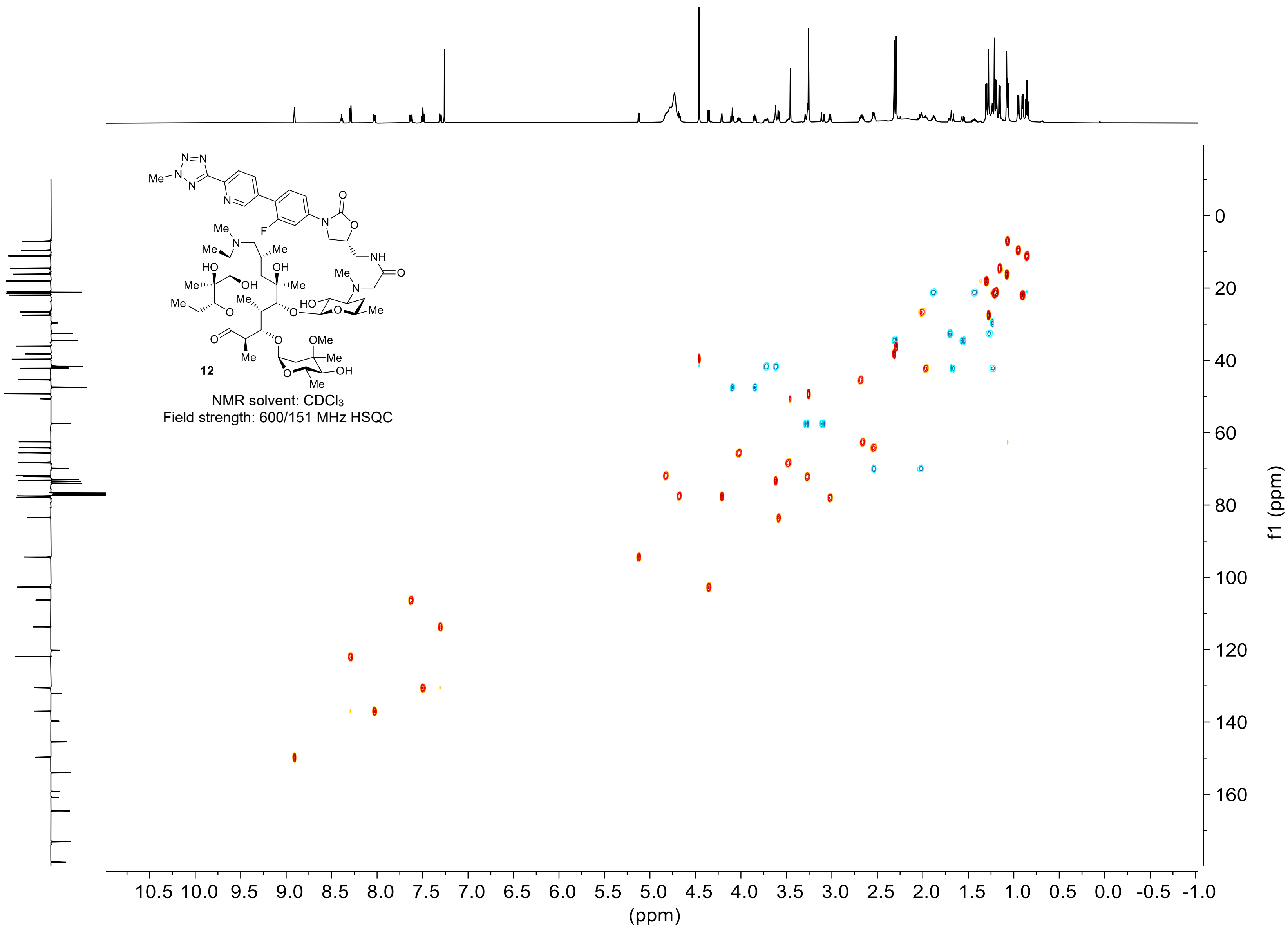

Figure S44. $^1H$–$^{13}C$-HSQC NMR spectrum (600.1/150.9 MHz; $CDCl_3$ + drop of 50% (v/v) $NH_4OH_{aq}$) of **12**.

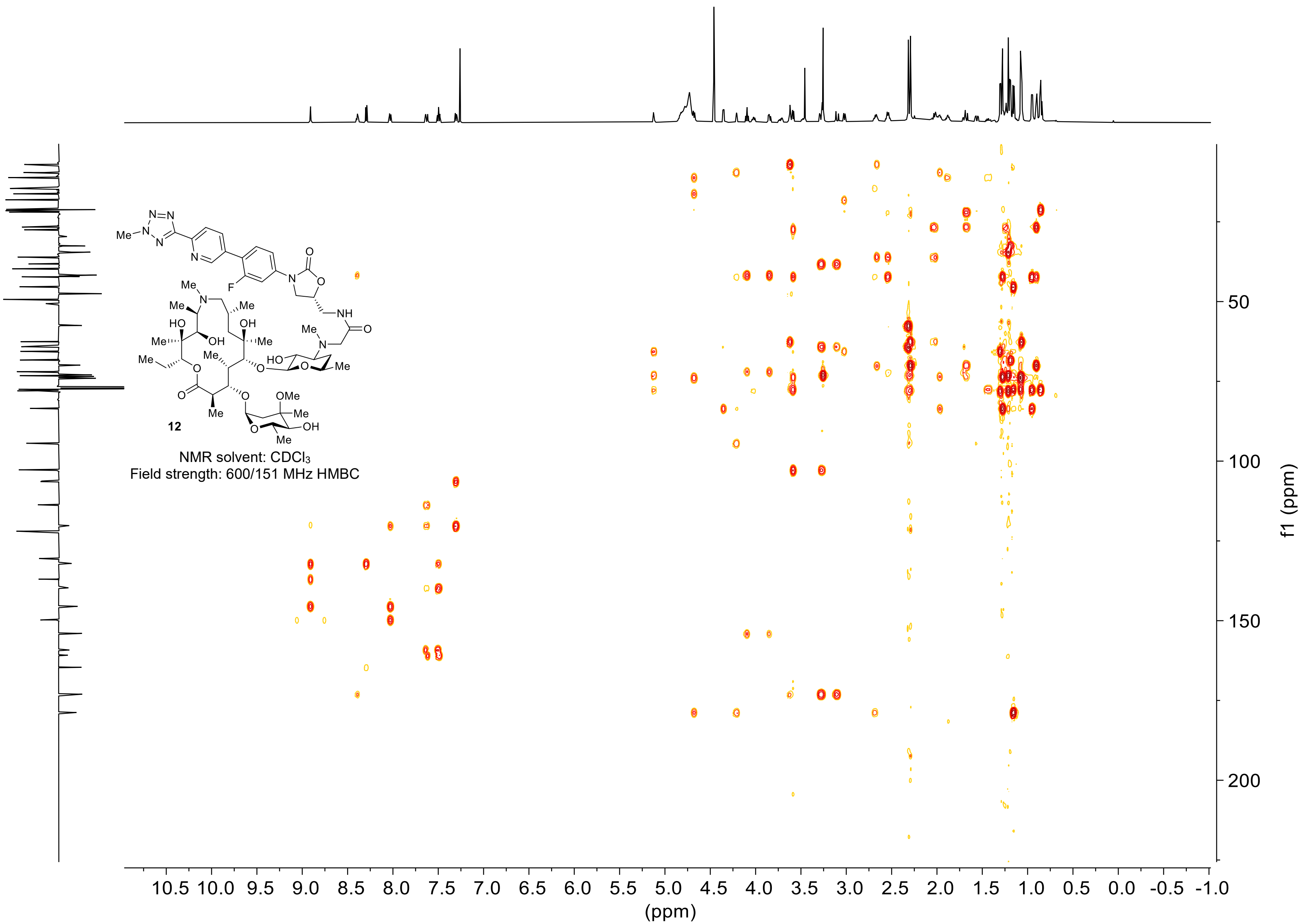


Figure S45. $^{1}H$–$^{13}C$-HMBC NMR spectrum (600.1/150.9 MHz; $CDCl_3$ + drop of 50% (v/v) $NH_4OH_{aq}$) of **12**.

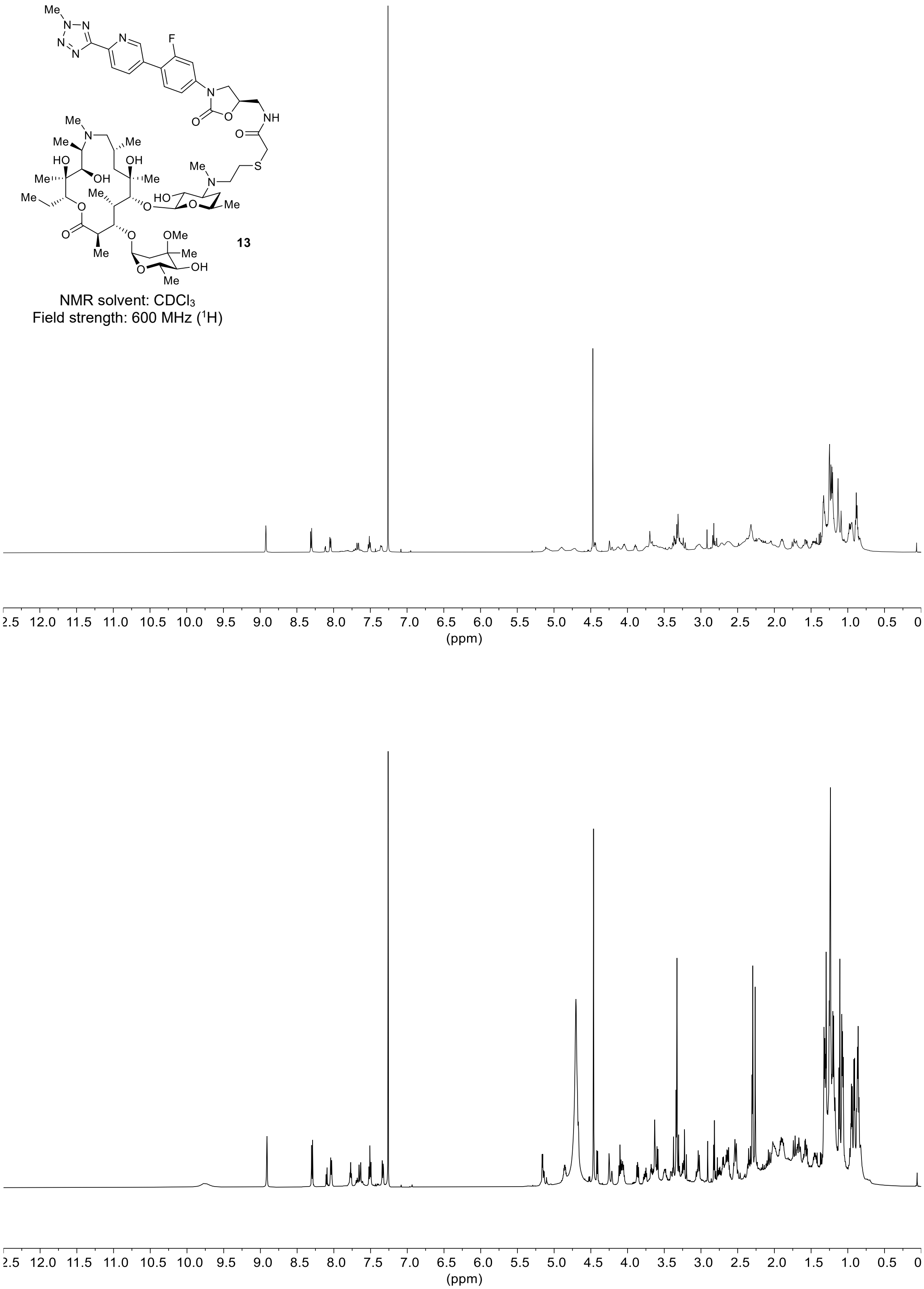


Figure S46. $^{1}$H-NMR spectra (600.1 MHz, $CDCl_3$) of **13** before (top) and after (bottom) addition of one drop of 50% (v/v) $NH_4OH_{aq}$.

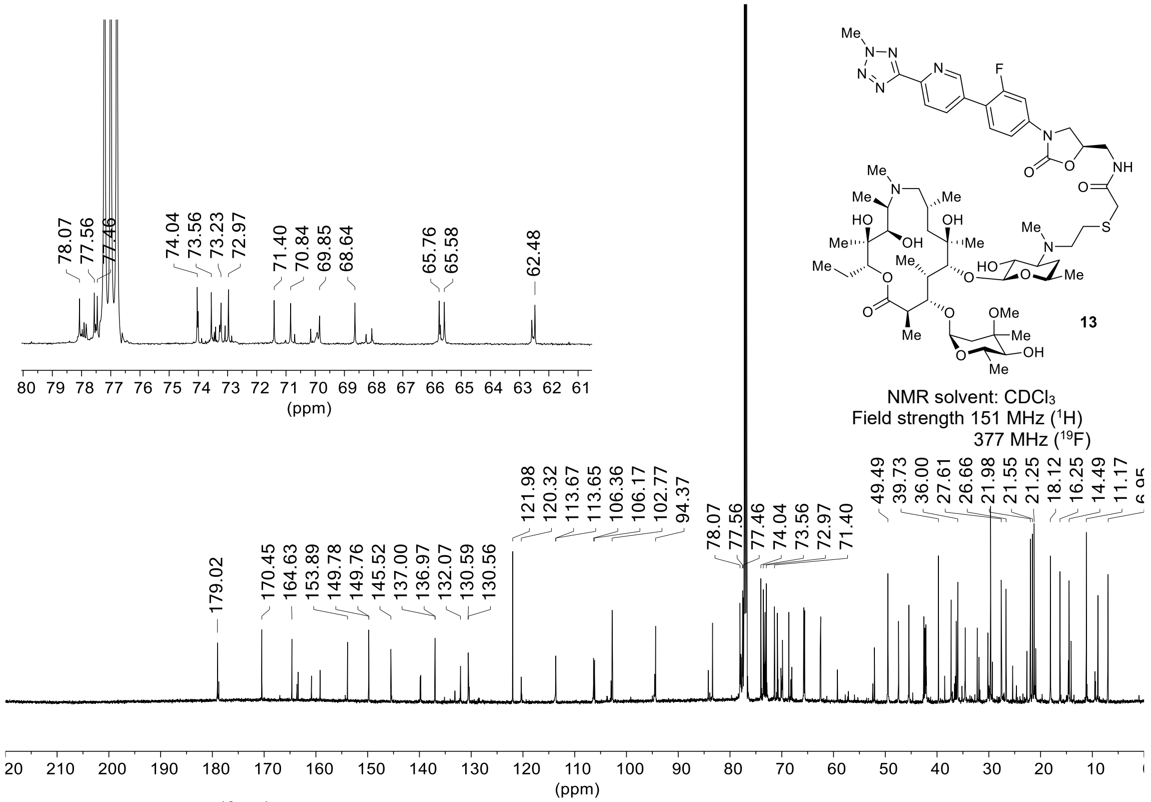


Figure S47. $^{13}C\{^{1}H\}$-NMR spectrum (150.9 MHz; $CDCl_3$ + drop of 50% (v/v) $NH_4OH_{aq}$) of **13**.

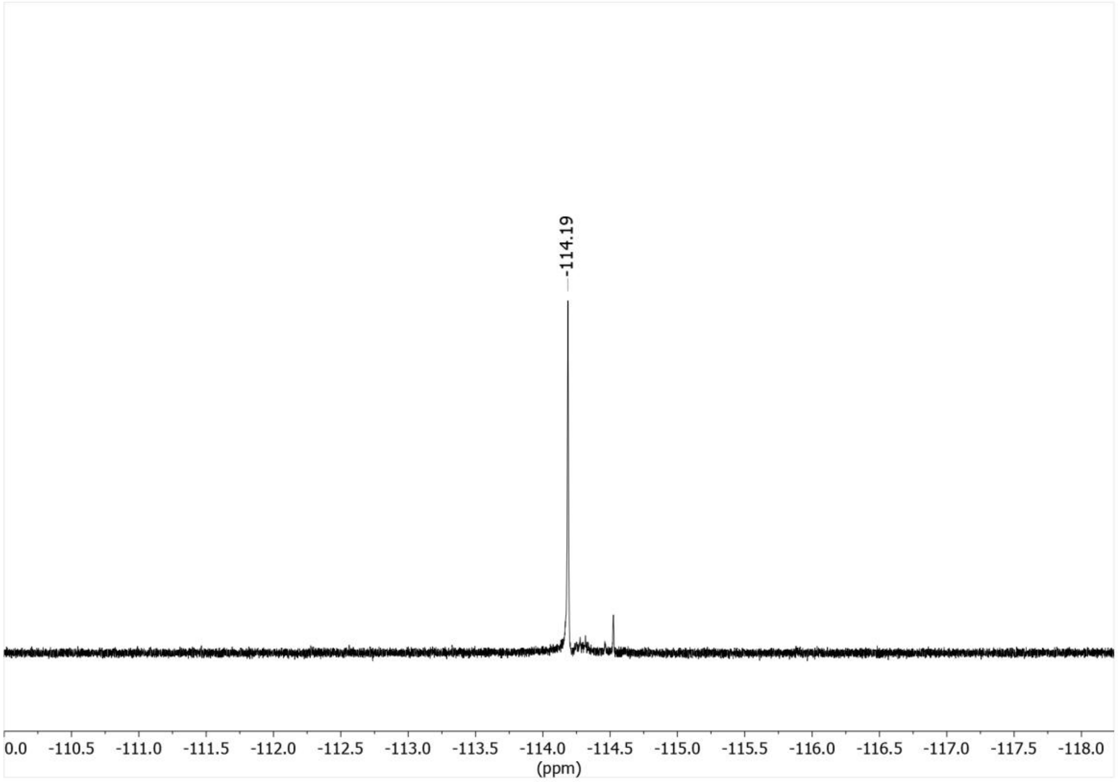


Figure S48. $^{19}F\{^{1}H\}$-NMR spectrum (376.5 MHz; $CDCl_3$ + drop of 50% (v/v) $NH_4OH_{aq}$) of **13**.

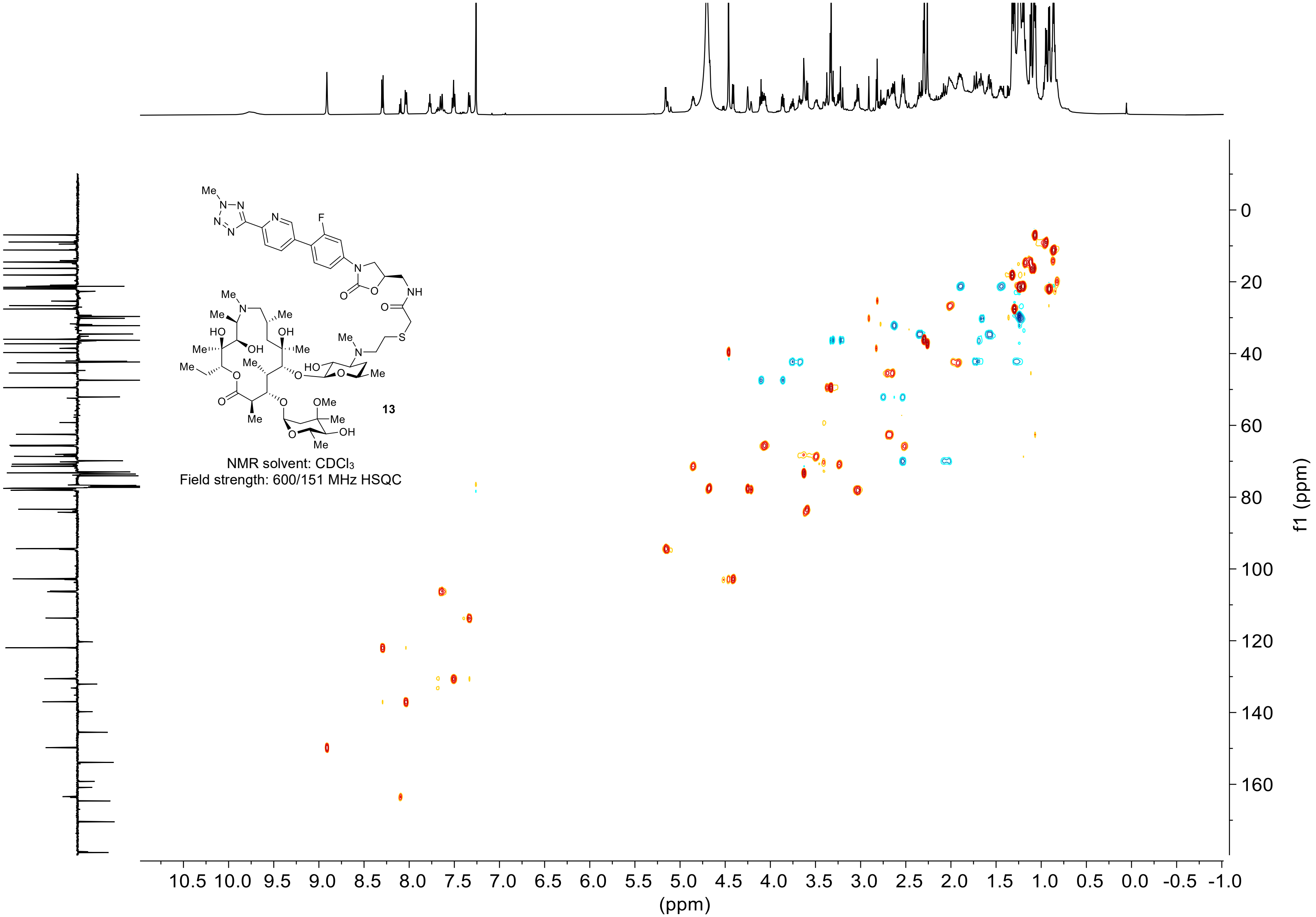


Figure S49. $^{1}H$–$^{13}C$-HSQC NMR spectrum (600.1/150.9 MHz; $CDCl_3$ + drop of 50% (v/v) $NH_4OH_{aq}$) of **13**.

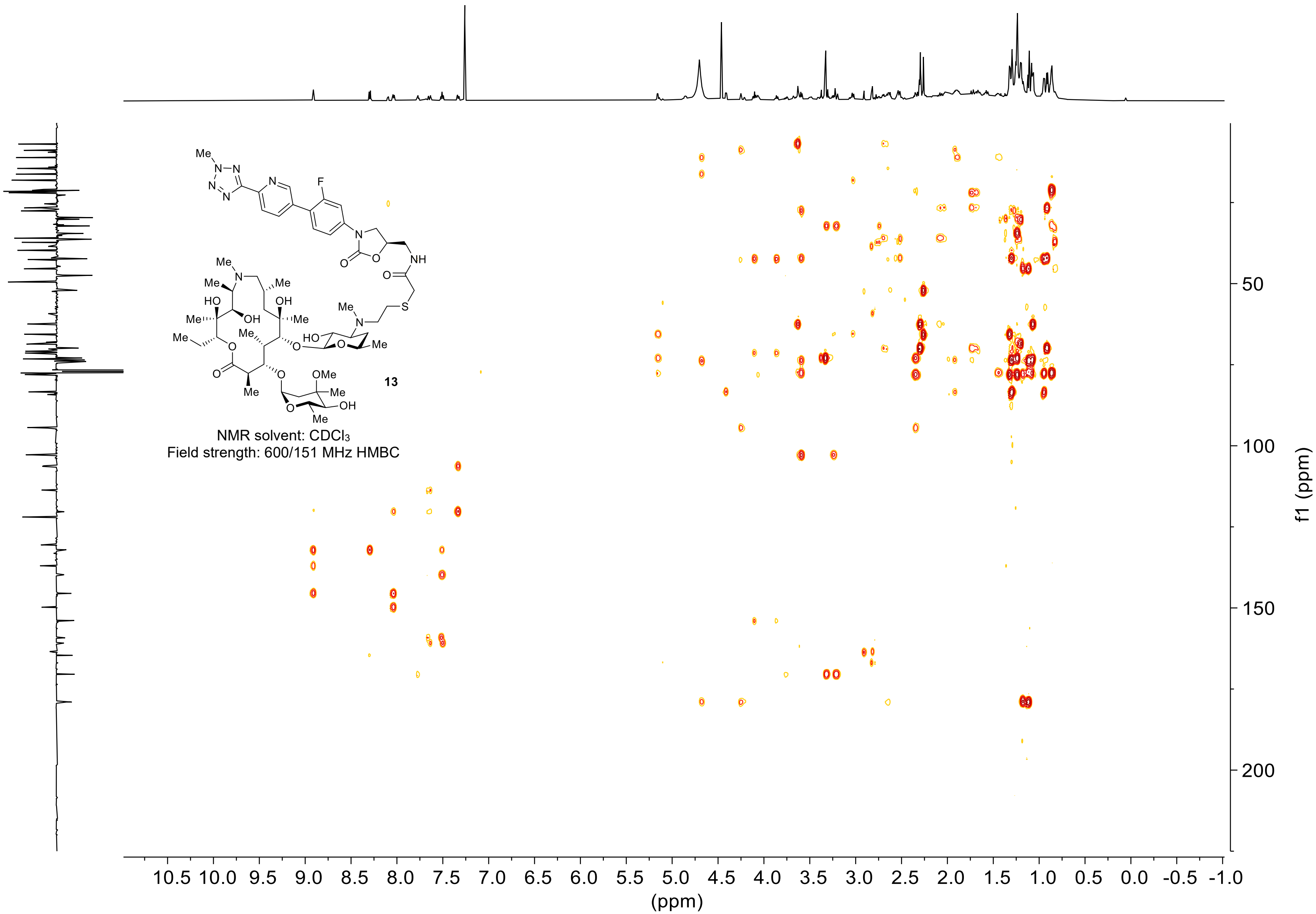


Figure S50. $^{1}H$–$^{13}C$-HMBC NMR spectrum (600.1/150.9 MHz; $CDCl_3$ + drop of 50% (v/v) $NH_4OH_{aq}$) of **13**.

## Literature


1 Li, Q.; Pellegrino, J.; Lee, D. J.; Tran, A. A.; Chaires, H. A.; Wang, R. et al. Synthetic group A streptogramin antibiotics that overcome Vat resistance. *Nature* **2020**, *586*, 145–150.

2 Cheng, A.; Eng, E. T.; Alink, L.; Rice, W. J.; Jordan, K.D.; Kim, L. Y. et al. High resolution single particle cryo-electron microscopy using beam-image shift. *J. Struct. Biol.* **2018**, *204*, 270–275.

3 Mastronarde, D. N. Automated electron microscope tomography using robust prediction of specimen movements. *J. Struct. Biol.* **2005**, *152*, 36–51.

4 Zheng, S. Q.; Palovcak, E.; Armache, J.-P.; Verba, K. A.; Cheng, Y.; Agard, D. A. MotionCor2: anisotropic correction of beam-induced motion for improved cryo-electron microscopy. *Nat. Methods* **2017**, *14*, 331–332.

5 Rohou, A.; Grigorieff, N. CTFFIND4: Fast and accurate defocus estimation from electron micrographs. *J. Struct. Biol.* **2015**, *192*, 216–221.

6 Watson, Z. L.; Ward, F. R.; Méheust, R.; Ad, O.; Schepartz, A.; Banfield, J. F. et al. Structure of the bacterial ribosome at 2 Å resolution, *eLife* **2020**, *9*, e60482.

7 Rosenthal, P. B.; Henderson, R. Optimal determination of particle orientation, absolute hand, and contrast loss in single-particle electron cryomicroscopy. *J. Mol. Biol.* **2003**, *333*, 721–745.

8 Punjani, A.; Rubinstein, J. L.; Fleet, D. J.; Brubaker, M. A. cryoSPARC: algorithms for rapid unsupervised cryo-EM structure determination. *Nat. Methods* **2017**, *14*, 290–296.

9 Moriarty, N. W.; Grosse-Kunstleve, R. W.; Adams, P. D. electronic Ligand Builder and Optimization Workbench (eLBOW): a tool for ligand coordinate and restraint generation. *Acta Crystallogr. D Biol. Crystallogr.* **2009**, *65*, 1074–1080.

10 Adams, P. D.; Afonine, P. V.; Bunkóczi, G.; Chen, V. B.; Davis, I. W.; Echols, N. et al. PHENIX: a comprehensive Python-based system for macromolecular structure solution. *Acta Crystallogr. D Biol. Crystallogr.* **2010**, *66*, 213–221.

11 Emsley, P.; Cowtan, K. Coot: model-building tools for molecular graphics. *Acta Crystallogr. D Biol. Crystallogr.* **2004**, *60*, 2126–2132.
