# Supplementary figures and images for: Hybrid Antibiotics Targeting the Bacterial Ribosome
Source: ACS Cent Sci. 2025 Sep 17;11(11):2133–42. doi: 10.1021/acscentsci.5c01046 (PMC12670282; doi:10.1021/acscentsci.5c01046)

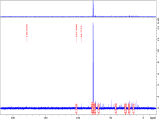

Supplement: Supplementary file 3 [file oc5c01046_si_003.zip › RawData/Comp1-DeMe-AZI/SLC-11_C_pub-240422/1/pdata/1/thumb.png]

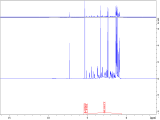

Supplement: Supplementary file 3 [file oc5c01046_si_003.zip › RawData/Comp1-DeMe-AZI/SLC-11_pub-240422/1/pdata/1/thumb.png]
