# Supplementary material for: Hybrid Antibiotics Targeting the Bacterial Ribosome
Source: ACS Cent Sci. 2025 Sep 17;11(11):2133–42. doi: 10.1021/acscentsci.5c01046 (PMC12670282; doi:10.1021/acscentsci.5c01046)
Supplement: Supplementary file 4 [file oc5c01046_si_004.pdf]

oc-2025-01046q.R1

Name: Peer Review Information for "Hybrid antibiotics targeting the bacterial ribosome"

First Round of Reviewer Comments

Reviewer: 1

Comments to the Author

This study presents the rational design, synthesis, and structural analysis of hybrid antibiotics that target the bacterial ribosome by simultaneously engaging the peptidyl transferase center (PTC) and the nascent peptide exit tunnel (NPET). By combining pharmacophores from azithromycin, tedizolid, and chloramphenicol, the authors engineered dual-binding compounds aimed at overcoming key resistance mechanisms in Gram-positive pathogens. Cryo-electron microscopy (cryo-EM) provided high-resolution structural insight, revealing that effective ribosomal engagement requires not only correct pharmacophore selection but also precise linker geometry to achieve optimal placement and binding.

Among the compounds evaluated, a tedizolid-azithromycin hybrid (compound 13) exhibited potent antibacterial activity against a broad panel of Gram-positive strains, including those resistant to macrolides and oxazolidinones. Hybrid 11, which also engaged both ribosomal sites, showed improved mitochondrial toxicity profiles relative to tedizolid but lacked expanded antibacterial activity. Cryo-EM structures of multiple hybrids highlighted how even subtle alterations in linker design or orientation could drastically alter binding pose and efficacy.

Despite the elegant structural work and thoughtful synthetic design, the study does not provide definitive evidence that these hybrids offer functional superiority over established antibiotics. Hybrid 13 retained the same mitochondrial toxicity as tedizolid, and neither hybrid demonstrated robust, consistent advantages in overcoming resistance pathways. The findings underscore the complexity of hybrid antibiotic development and suggest that

the hybrid approach, while promising in concept, requires further refinement to yield therapeutically superior candidates. One of the more notable issues is the discrepancy between activity and inhibition in several cases – a discrepancy that will undoubtedly require further significant study.

Overall, despite the above limitations, this work establishes a valuable framework for hybrid antibiotic design and structural validation, particularly in targeting adjacent ribosomal sites. While the conclusions regarding therapeutic advantage remain preliminary, the study represents a rigorous and insightful integration of synthetic chemistry, high-resolution structural biology, and comprehensive microbiological evaluation. The depth and quality of the work warrant publication in ACS Central Science, where it will serve as a significant and timely addition to the antibiotic discovery and ribosome-targeting literature.

Detailed Suggested Edits:

Page 2:

- Lines 6-7: Consider adjusting the tone of this statement, as it is not evident that any compound enhances activity.

Page 3:

- Lines 4-9: Consider presenting this section in a schematic format to enhance clarity and accessibility for readers.

Page 4:

- Line 40: "with a for promising" contains grammatical errors. Please revise.
- Lines 51-52: Even though the target audience may not be primarily chemists, it would strengthen the manuscript to justify the selectivity and differences in reaction conditions compared to previous work.

Page 5:

- Figure 2: The SI reports a 73% yield for the first step, while the main text reports 51%. Please clarify or correct this inconsistency.

Page 6:

- Lines 4-6: "The inefficient... function" requires supporting proof. If AZI binding is normal, compounds 6 and 7 should exhibit similar translation inhibition; the reason for the observed differences is unclear and should be explained.

Page 7:

- The rationale for using a 2-mercaptoethanol linker needs clarification.
- Line 19: "methanesulfonylation" is incorrect. Compound 10 is not a sulfonate; use the correct terminology (likely sulfide).
- Additionally, the authors claim that compound 9 shows a reversible binding mode with the ribosome and improved activity; however, they do not provide data or discussion on the safety profile of this compound. Can this become a therapeutic candidate?

Page 8:

- Replace "chloroacetamide function" with "chloroacetamide functional group" for precision and clarity.

Page 12:

- Lines 13-14: Although there is a significant increase in MIC for TDZ compared to only a 2-fold increase for the hybrid, the authors do not explain why this discrepancy occurs or provide sufficient reasoning for this observation. Clarification with supporting data would strengthen the discussion.

Supporting Information:

- The proton NMR spectra do not show integrations, making it difficult to confirm compound purity and structural assignments.
- Additionally, the chemical shifts are not labelled on the spectra, which should be corrected to enable proper interpretation.

Reviewer: 2

Comments to the Author

This manuscript from Seiple and colleagues reports on a new class of 'hybrid' antibiotics – that is, two known antibiotics linked together – that target the bacterial ribosome. The sort of hybrid strategy pursued here, where antibiotics that bind proximal to one another in a single target are linked together, makes more intuitive sense than the covalent linkage of two antibiotics that hit different targets. The featured compound from this work (13) has some promising activity, and is backed by impressive structural biology. The ability to use CryoEM to design new hybrids, as they are doing here, could be very powerful as demonstrated by this work.

In general this document is extremely clear and well-written, with the narrative arc lucid and scholarly. And, synthesis of these large and complex molecules is no easy feat, and the authors develop several nifty and efficient transformations that will be useful to others, even in this field where there are many macrolides and oxazolindinones in existence. I have read this manuscript closely and carefully considered its message, and I do think it is suitable for publication in ACS Central Science after some modification.

Major point:

The most negative view of this work would be that the authors have produced a less active version of TDZ. Looking at Table 1, while compound 13 is not subject to macrolide resistance, it could be because the TDZ portion is doing all the work. Indeed, there seem to be no cases where 13 is more active than TDZ and mostly it is worse. This is really the crux of the hybrid design – a "best of both worlds" from the two MOAs, including overcoming of

resistance. The authors get to this a bit in the Discussion, noting a few cell lines where MICs for TDZ get worse but are unchanged for 13, but this is a modest and subtle effect.

Since the dual mode-of-action/hybrid nature of these antibiotics is the critical element here, I do think this point deserves more investigation to demonstrate the possibilities for overcoming both resistance mechanisms, as postulated. For example, if one used the stain in entry 17 (Table 1) and generated resistance mutants, would they be consistent with macrolide resistance and not TDZ resistance? That would be nice to see.

[the other argument the authors make for the hybrid antibiotic is mitigation of toxicity, but as shown in Figure S1 these compounds do not have that effect]

Minor Points:

- In the introduction the authors review other efforts at hybrid antibiotics, including those that seem to be closely related to this new work including other examples of macrolide-oxazolidinone hybrids. It would be useful for them to return to this literature in the Discussion, to say how 11 and 13 and related compounds might have an advantage over these earlier efforts.

- There are a few times where the authors invoke reduced cellular accumulation to explain poor MICs, but accumulation in Gram-positive bacteria is not typically an issue and regardless would not be predicted to be altered for some of these compounds with minor changes (eg, Me in AZI □ propargyl in 2). I suggest that the authors modify their verbiage; alternatively, they could measure accumulation or assess compounds in strains where the membrane is compromised.

- There are a few minor typos ("b" in the Figure 1 legend should be "c", etc) so the manuscript should be read carefully.

- The Supporting Figures, containing important experimental info, are mixed with the archival spectral data. Supporting Figures should be first in the SI and called out as such ("Supporting Figure 1") in the text.

Reviewer: 3

Comments to the Author

In this study, Yeon et al. present the design, synthesis, structural, and microbiological characterization of a series of hybrid antibiotics combining azithromycin (AZI) with either chloramphenicol (CHL) or tedizolid (TDZ). These hybrids are intended to span two key binding regions of the bacterial ribosome, the peptidyl transferase center and the nascent peptide exit tunnel, thereby leveraging dual-site engagement to overcome known resistance mechanisms. The authors synthesized a panel of hybrid molecules and characterized their antimicrobial activities in vitro. Five hybrids were visualized bound to the E. coli ribosome using cryo-EM, revealing varying degrees of conventional placement and flexibility of their component pharmacophores. Among the synthesized compounds, two hybrids - compound 11 and especially compound 13 – demonstrated improved activity over azithromycin across several resistant Gram-positive strains. Cryo-EM structures provided mechanistic insight into how linker geometry affects drug positioning and binding site engagement. However, none of the hybrids surpassed the antimicrobial potency of tedizolid, and only compound 11 showed modestly reduced mitochondrial toxicity.

This manuscript clearly represents a significant synthetic and structural effort. The synthesis of multiple complex hybrid antibiotics is commendable, and the structural studies involving high-resolution cryo-EM are particularly strong. The authors' integration of chemistry, structural biology, and microbiological profiling is a key strength, and the cryo-EM data are of high quality. A particularly engaging aspect of the manuscript is the Introduction section, which provides an excellent historical perspective on prior hybrid antibiotic efforts, including the notable work conducted at Rib-X. That said, the primary limitation of the work lies in the overall antimicrobial activity of the hybrids relative to the parent compounds, particularly tedizolid.

Compound 13, which appears to be the most promising of the hybrids, does indeed show improved MIC values compared to azithromycin across a resistant strain panel. However, its performance is significantly inferior to tedizolid across nearly all strains (as shown in Table 1). Thus, the conclusion that 13 “overcomes resistance” requires clarification. While

compound 13 can engage Erm-modified ribosomes and shows reduced sensitivity to some resistance mechanisms that impact azithromycin, its antimicrobial activity appears primarily driven by the retained functionality of the tedizolid warhead. In other words, rather than creating an "improved macrolide", the authors seem to have generated a somewhat compromised oxazolidinone. This interpretation is especially clear when noting that in no case does compound 13 outperform either of its parent compounds. The only exception of potential interest is compound 11, which demonstrates 2-fold improved activity over tedizolid in S. pyogenes and S. pneumoniae wild-type strains, though not against their resistant counterparts expressing erm genes.

In summary, this work represents a technically impressive and comprehensive study. The chemistry and structural biology are elegant, and the manuscript is well written. However, it is unfortunate that none of the hybrid compounds exceed the antimicrobial potency of either parent drug. This suggests that, at least in their current form, these conjugates may not offer clear therapeutic advantages over existing treatments such as azithromycin or tedizolid. The study could be significantly strengthened by evaluating the most promising compounds (e.g., 13) in in vivo infection models to test whether improved pharmacokinetics or selective toxicity might compensate for their diminished potency in MIC assays. Such experiments could uncover clinically meaningful advantages that are not apparent from the reported in vitro studies.

Author's Response to Peer Review Comments:

Please see attached for response to reviewers. All editorial requests were addressed as well.

Point-by-point response to reviewers

Hybrid antibiotics targeting the bacterial ribosome (manuscript oc-2025-01046q).

Below are our point-by-point responses to the reviewers' comments. The original comments are in blue; our responses are in black. The comments from all three reviewers led to a substantially improved manuscript, and we are grateful for them.

Reviewer(s)' Comments to Author:

Reviewer: 1

Recommendation: Publish in ACS Central Science after minor revisions noted.

Comments:
This study presents the rational design, synthesis, and structural analysis of hybrid antibiotics that target the bacterial ribosome by simultaneously engaging the peptidyl transferase center (PTC) and the nascent peptide exit tunnel (NPET). By combining pharmacophores from azithromycin, tedizolid, and chloramphenicol, the authors engineered dual-binding compounds aimed at overcoming key resistance mechanisms in Gram-positive pathogens. Cryo-electron microscopy (cryo-EM) provided high-resolution structural insight, revealing that effective ribosomal engagement requires not only correct pharmacophore selection but also precise linker geometry to achieve optimal placement and binding.

Among the compounds evaluated, a tedizolid-azithromycin hybrid (compound 13) exhibited potent antibacterial activity against a broad panel of Gram-positive strains, including those resistant to macrolides and oxazolidinones. Hybrid 11, which also engaged both ribosomal sites, showed improved mitochondrial toxicity profiles relative to tedizolid but lacked expanded antibacterial activity. Cryo-EM structures of multiple hybrids highlighted how even subtle alterations in linker design or orientation could drastically alter binding pose and efficacy.

Despite the elegant structural work and thoughtful synthetic design, the study does not provide definitive evidence that these hybrids offer functional superiority over established antibiotics. Hybrid 13 retained the same mitochondrial toxicity as tedizolid, and neither hybrid demonstrated robust, consistent advantages in overcoming resistance pathways. The findings underscore the complexity of hybrid antibiotic development and suggest that the hybrid approach, while promising in concept, requires further refinement to yield therapeutically superior candidates. One of the more notable issues is the discrepancy between activity and inhibition in several cases – a discrepancy that will undoubtedly require further significant study.

Overall, despite the above limitations, this work establishes a valuable framework for hybrid antibiotic design and structural validation, particularly in targeting adjacent ribosomal sites. While the conclusions regarding therapeutic advantage remain preliminary, the study represents a rigorous and insightful integration of synthetic chemistry, high-resolution structural biology, and comprehensive microbiological evaluation. The depth and quality of the work warrant publication in ACS Central Science, where it will serve as a significant and timely addition to the antibiotic discovery and ribosome-targeting literature.

Detailed Suggested Edits:

Page 2:
- Lines 6-7: Consider adjusting the tone of this statement, as it is not evident that any compound enhances activity.

Response: We have revised the statement and the next sentence to: “These results extend our understanding of ribosome inhibition and provide a framework for the rational design of dualaction antibiotics that target the ribosome. In a broader context, this work offers a framework for developing bifunctional inhibitors that engage adjacent binding sites by means of a rational cycle of synthetic optimization, biological evaluation, and structural characterization.”

Page 3:

- Lines 4-9: Consider presenting this section in a schematic format to enhance clarity and accessibility for readers.

Response: Excellent suggestion. We have added a figure to the Supporting Information (Figure S##) that shows the structures of each previous hybrid that was mentioned in the manuscript, along with a short summary of activity.

Page 4:

- Line 40: "with a for promising" contains grammatical errors. Please revise.

Response: corrected.

- Lines 51-52: Even though the target audience may not be primarily chemists, it would strengthen the manuscript to justify the selectivity and differences in reaction conditions compared to previous work.

Response: We have expanded this section to detail our optimization of Andrade’s previously reported conditions for ketolides:

“To enable attachment to **AZI**, we mono-demethylated the desosamine 3’ dimethylamine, providing a monomethylamine that could be used directly for hybridization or decorated with a linker. Unlike other macrolides such as erythromycin and solithromycin, **AZI**’s additional amine at position 9a within the macrocycle posed a potential selectivity challenge (it could be demethylated in competition with the 3’ dimethylamine). We found that conditions previously published by Andrade and coworkers for ketolides, which comprised iodine and sodium acetate in refluxing methanol/water, led to low yields on **AZI**.[29] However, we found that the inclusion of Tris buffer and a lower temperature (50 ºC) led to reproducibly good yields of hybrid precursor **1** (73%, **Figure 2**).”

Page 5:

- Figure 2: The SI reports a 73% yield for the first step, while the main text reports 51%. Please clarify or correct this inconsistency.

Response: Thanks for catching this – the 51% yield was from an older chemdraw file, and the 73% yield (reported in the SI) is correct and reproducible. We have corrected the figure.

Page 6:

- Lines 4-6: "The inefficient... function" requires supporting proof. If AZI binding is normal, compounds 6 and 7 should exhibit similar translation inhibition; the reason for the observed differences is unclear and should be explained.

Response: This is a valid point. Since **7** does have the ability to inhibit ribosomes in vitro (20% residual translation at 10 µM), the placement of the chloramphenicol portion does not explain the lack of activity. We have removed the sentence.

We have also modified the first paragraph of the Discussion section to:

"The design of active hybrid antibiotics is inherently challenging. Accumulation in bacterial cells can be hard to predict and assess.[40,41] complicating hybrid design. Differences in size and exposed polar surface area of the hybrids compared to their individual components can have profound effects on cellular accumulation, and thus activity in MIC assays. AZI-CHL **7**, for example, is capable of inhibiting translation in vitro but does not inhibit the growth of *S. aureus* even at 512 µg/mL. "

Page 7:

- The rationale for using a 2-mercaptoethanol linker needs clarification.

Response: 2-Mercaptoethanol provided a convenient method to extend the linker without major revision of the route. The alcohol in 2-mercaptoethanol was left unprotected and unactivated while coupling to the TDZ acetamide was accomplished (step 1). Then, mesylation of the alcohol led to the electrophile **10** that could be coupled with **1** to reach the extended-linker hybrid.

- Line 19: "methanesulfonylation" is incorrect. Compound 10 is not a sulfonate; use the correct terminology (likely sulfide).

Response: While 10 does indeed have a sulfide/thioether, it also has a methanesulfonyl (or mesyl) group.

- Additionally, the authors claim that compound 9 shows a reversible binding mode with the ribosome and improved activity; however, they do not provide data or discussion on the safety profile of this compound. Can this become a therapeutic candidate?

Response: This is in an intriguing question. The chloroacetamide group, which is not highly hindered, is likely too reactive for this compound to safely be used in vivo, as reactivity with

nucleophilic cysteines and other thiols would lead to off-target toxicity. Indeed, chloroacetamides are used as promiscuous probes, and are generally not found in drug candidates.

We have revised the text to read:

"The enhanced cellular activity of **9** may arise from increased accumulation or from off-target effects on *S. aureus* proteins owing to its reactive chloroacetamide. This hypothesis is supported by recent data from the Hacker group that measured engagement of 230 cysteines in the *S. aureus* proteome with a promiscuous chloroacetamide alkyne probe,[35] and likely tampers any therapeutic potential of this molecule."

Page 8:

- Replace "chloroacetamide function" with "chloroacetamide functional group" for precision and clarity.

Response: Revised throughout the manuscript.

Page 12:

- Lines 13-14: Although there is a significant increase in MIC for TDZ compared to only a 2-fold increase for the hybrid, the authors do not explain why this discrepancy occurs or provide sufficient reasoning for this observation. Clarification with supporting data would strengthen the discussion.

Response: This observation inspired us to examine the structure of the ABC-F resistance protein PoxtA bound to the ribosome (Nat. Comm. **2022**, doi: 10.1038/s41467-022-29274-9) to evaluate whether there could be a structural explanation for the inability of these proteins to confer resistance to **13**. The authors of that study hypothesize that PoxtA binds to antibioticstalled (in that case chloramphenicol, but PoxtA also affects linezolid) ribosomes, which are charged with P-site and A-site tRNA but cannot complete the peptidyltransferase reaction. Importantly, this happens during elongation, when several amino acids have already been added to the nascent chain. After successful binding by PoxtA (and hydrolysis of ATP), binding of the antibiotic is disrupted, and the drug diffuses away, allowing translation to resume.

We have added a substantial section to the discussion section on this topic, and we have added an overlay of the PoxtA ribosome structure with our ribosome-bound structure of **13** to the supporting information (Figure S4).

We added the following paragraph, along with minor changes throughout the manuscript, to expand on this topic:

"The activity of hybrid **13** against strains with phenicol-oxazolidinone (PhO) resistance mechanisms merits further discussion. Phenicols (such as **CHL**) and oxazolidinones (such as **TDZ**) are context-specific translation inhibitors that preferentially bind to ribosomes occupied by

P-site tRNA and a nascent chain that contains alanine (and to a lesser extent, serine or threonine) at the penultimate (-1) position.[42-43] The ABC-F proteins PoxtA and OptrA bind to stalled ribosomes in the E-site with an Antibiotic Resistance Domain (ARD) extended towards the catalytic center and interacts with P-site tRNA and the nascent polypeptide.[60] Upon successful binding, it is hypothesized that the ABC-F protein induces a structural change that leads to antibiotic dissociation, possibly by perturbing the positioning of the nascent chain and disrupting its interaction with the drug. This results in resistance to phenicols and oxazolidinones, which we observe with **TDZ** in both *E. faecalis* (4- to 16-fold increase of MIC compared to WT, entries 1517) and in *E. faecium* (4- to 8-fold increase in MIC, entries 24-25). Unlike **TDZ**, hybrid **13** is minimally affected by these ABC-F resistance mechanisms (2-fold increase in MIC in *E. faecalis* and no change in MIC in *E. faecium*). An overlay of our ribosome-bound structure of **13** with the PoxtA/P-site tRNA-bound structure (Figure S3) reveals no obvious direct clash with the PoxtA ARD or with P-site tRNA, but **13** does occupy the space where the nascent chain would reside. Possible structural explanations of this lack of susceptibility to ABC-F resistance include 1) inability of the ARD to effectively position itself in the presence of **13**, 2) the position of P-site tRNA and the nascent chain in the presence of **13** precludes ABC-F binding, or 3) hybrid **13** acts as an initiation inhibitor, and the inhibited initiation complex is not a substrate for PoxtA or OptrA. Future work will focus on microbiological and structural studies to differentiate between these putative molecular mechanisms and on structural modifications to oxazolidinones to achieve the same result without attachment of an entire macrolide antibiotic."

Supporting Information:

- The proton NMR spectra do not show integrations, making it difficult to confirm compound purity and structural assignments.
- Additionally, the chemical shifts are not labelled on the spectra, which should be corrected to enable proper interpretation.

Response: We have added integrations to the images found of the spectra in the SI document. We chose not to include peak picking in the NMR images because it was not possible to label all of the peaks within a standard page size, especially for the larger hybrid compounds. Of course, all peaks are listed in the synthesis section of the SI.

We have also included all of the raw NMR data in a zip file as an additional attachment. Please feel free to open it in an NMR program of your choice and analyze as you see fit.

Additional Questions:
Quality of experimental data, technical rigor: Top 10%

Significance to chemistry researchers in this and related fields: Top 10%

Broad interest to other researchers: Top 10%

Novelty: Top 10%

Is this research study suitable for media coverage or a First Reactions (a News & Views piece in the journal)?: No

Reviewer: 2

Recommendation: Publish in ACS Central Science after minor revisions noted.

Comments:
This manuscript from Seiple and colleagues reports on a new class of ‘hybrid’ antibiotics – that is, two known antibiotics linked together – that target the bacterial ribosome. The sort of hybrid strategy pursued here, where antibiotics that bind proximal to one another in a single target are linked together, makes more intuitive sense than the covalent linkage of two antibiotics that hit different targets. The featured compound from this work (13) has some promising activity, and is backed by impressive structural biology. The ability to use CryoEM to design new hybrids, as they are doing here, could be very powerful as demonstrated by this work.

In general this document is extremely clear and well-written, with the narrative arc lucid and scholarly. And, synthesis of these large and complex molecules is no easy feat, and the authors develop several nifty and efficient transformations that will be useful to others, even in this field where there are many macrolides and oxazolindinones in existence. I have read this manuscript closely and carefully considered its message, and I do think it is suitable for publication in ACS Central Science after some modification.

Major point:
The most negative view of this work would be that the authors have produced a less active version of TDZ. Looking at Table 1, while compound 13 is not subject to macrolide resistance, it could be because the TDZ portion is doing all the work. Indeed, there seem to be no cases where 13 is more active than TDZ and mostly it is worse. This is really the crux of the hybrid design – a “best of both worlds” from the two MOAs, including overcoming of resistance. The authors get to this a bit in the Discussion, noting a few cell lines where MICs for TDZ get worse but are unchanged for 13, but this is a modest and subtle effect.

Response: Thank you for this comment – we agree that this will be the reaction from most readers at first glance, and there were rooms for improvement in the way we discussed the MIC data of **13** vs **TDZ**. There are a few points that’d I’d like to address here, and we have also updated the manuscript to more clearly highlight the differences between **13** and **TDZ** and to discuss the potential of **13**.

- Hybrid **13** has had very little medicinal chemistry performed on it as a complete compound – I would consider it a “hit” not a “lead” (contrasted to **TDZ**, which is highly optimized in its final form). At this stage of the project, we have only explored three linkers. With further chemistry to explore different oxazolidinones (e.g. radezolid, contezolid), macrolide scaffolds (ketolide scaffold with varying aryl alkyl groups), or linkers, we are likely to lower the MICs against “susceptile” strains to match tedizolid. As it stands, it already equals or exceeds linezolid. We also hope to overcome Cfr resistance.

- Hybrid is as active or more active than the most commonly used oxazolidinone, linezolid, in all strains tested. We have added a table to the supporting information (Table S1) to highlight these differences and compare to other commonly used antibiotics.

- **TDZ** is susceptible to ABC-F resistance (PoxtA and OptrA), which raise its MICs in *E. faecium* and *E. faecalis* by 4- to 16-fold. Hybrid **13** is not susceptible to this resistance mechanism (max MIC difference is 2-fold), as we have highlighted in a new paragraph in the Discussion section. There are interesting mechanistic implications to overcoming this resistance mechanism, and further optimization of the scaffold could yield a molecule that is as potent as tedizolid in other strains but not susceptible to ABC-F resistance.
- Hybrid **13** is less susceptible to G2576T mutational resistance than tedizolid, is equipotent to TDZ against strains with mutant copy numbers in rRNA (see entries 20 and 29).

- In some cases, **13** has a lower MIC than tedizolid, albeit only by 2-fold. See entries 17 (which summarizes multiple strains) and 25 in Table 1.

Since the dual mode-of-action/hybrid nature of these antibiotics is the critical element here, I do think this point deserves more investigation to demonstrate the possibilities for overcoming both resistance mechanisms, as postulated. For example, if one used the stain in entry 17 (Table 1) and generated resistance mutants, would they be consistent with macrolide resistance and not TDZ resistance? That would be nice to see.

Response: We definitely agree! We have updated the text to discuss the resistance that 13 has already overcome (ABC-F) and future work will focus on exploring resistance mutants and overcoming Cfr resistance.

[the other argument the authors make for the hybrid antibiotic is mitigation of toxicity, but as shown in Figure S1 these compounds do not have that effect]

Comment: this was one of the more puzzling results, and we were very surprised about the toxicity. We are going to try to determine the molecular underpinnings of mitochondrial toxicity with cryo-EM in the mitoribosome, and optimize away from it.

Minor Points:

- In the introduction the authors review other efforts at hybrid antibiotics, including those that seem to be closely related to this new work including other examples of macrolideoxazolidinone hybrids. It would be useful for them to return to this literature in the Discussion, to say how 11 and 13 and related compounds might have an advantage over these earlier efforts.

Response: Great suggestion, we have revisited the previous work in the Discussion, where possible. The data for several of the initial reports from industrial groups (e.g., Rib-X) is sparse, so a head-to-head comparison is not always possible.

- There are a few times where the authors invoke reduced cellular accumulation to explain poor MICs, but accumulation in Gram-positive bacteria is not typically an issue and regardless would not be predicted to be altered for some of these compounds with minor changes (eg, Me in AZI propargyl in 2). I suggest that the authors modify their verbiage; alternatively, they could measure accumulation or assess compounds in strains where the membrane is compromised.

Response: I certainly agree that for most compounds it is much easier to achieve cellular accumulation in G+ bugs than G- bugs (with some exceptions such as select aminoglycosides). However, large molecules still have to be able to adopt a shape and have the right physicochemical properties to passively diffuse through the G+ membrane, and this can be particularly challenging when the molecules are >1000 amu and flexible. We run into this problem a lot with hybrid compounds.

That being said, you're certainly right that we overused that argument in our paper. The Me → propargyl change from **AZI** to **2** is a good example – thank you for pointing that out. If anything, **2** should have higher diffusion through the membrane due to better shielding of the amine. We have revised the verbiage to the following:

"The elevated MIC for **1** might be explained by the reduced shielding of the C3' desmethyl amine, which is likely to be protonated and will hinder passive diffusion across the membrane. The reduced cellular activity of **2**, however, is more challenging to explain as the C3' amine is more shielded; reduced activity for this compound may arise from another mechanism besides hindered accumulation."

- There are a few minor typos ("b" in the Figure 1 legend should be "c", etc) so the manuscript should be read carefully.

Response: Corrected several small typos and errors.

- The Supporting Figures, containing important experimental info, are mixed with the archival spectral data. Supporting Figures should be first in the SI and called out as such ("Supporting Figure 1") in the text.

Response: Great catch. The SI was reordered so that the chemistry experimental procedures, data, and spectra are at the end. Several new figures and a table were also added, and are correctly referenced in the text.

Additional Questions:
Quality of experimental data, technical rigor: Top 1%

Significance to chemistry researchers in this and related fields: Top 10%

Broad interest to other researchers: Top 10%

Novelty: Top 10%

Is this research study suitable for media coverage or a First Reactions (a News & Views piece in the journal)?: No

Reviewer: 3

Recommendation: Major revisions required.

Comments:
In this study, Yeon et al. present the design, synthesis, structural, and microbiological characterization of a series of hybrid antibiotics combining azithromycin (AZI) with either chloramphenicol (CHL) or tedizolid (TDZ). These hybrids are intended to span two key binding regions of the bacterial ribosome, the peptidyl transferase center and the nascent peptide exit tunnel, thereby leveraging dual-site engagement to overcome known resistance mechanisms. The authors synthesized a panel of hybrid molecules and characterized their antimicrobial activities in vitro. Five hybrids were visualized bound to the E. coli ribosome using cryo-EM, revealing varying degrees of conventional placement and flexibility of their component pharmacophores. Among the synthesized compounds, two hybrids - compound 11 and especially compound 13 – demonstrated improved activity over azithromycin across several resistant Gram-positive strains. Cryo-EM structures provided mechanistic insight into how linker geometry affects drug positioning and binding site engagement. However, none of the hybrids surpassed the antimicrobial potency of tedizolid, and only compound 11 showed modestly reduced mitochondrial toxicity.

This manuscript clearly represents a significant synthetic and structural effort. The synthesis of multiple complex hybrid antibiotics is commendable, and the structural studies involving highresolution cryo-EM are particularly strong. The authors' integration of chemistry, structural biology, and microbiological profiling is a key strength, and the cryo-EM data are of high quality. A particularly engaging aspect of the manuscript is the Introduction section, which provides an

excellent historical perspective on prior hybrid antibiotic efforts, including the notable work conducted at Rib-X. That said, the primary limitation of the work lies in the overall antimicrobial activity of the hybrids relative to the parent compounds, particularly tedizolid.

Compound 13, which appears to be the most promising of the hybrids, does indeed show improved MIC values compared to azithromycin across a resistant strain panel. However, its performance is significantly inferior to tedizolid across nearly all strains (as shown in Table 1). Thus, the conclusion that 13 "overcomes resistance" requires clarification. While compound 13 can engage Erm-modified ribosomes and shows reduced sensitivity to some resistance mechanisms that impact azithromycin, its antimicrobial activity appears primarily driven by the retained functionality of the tedizolid warhead. In other words, rather than creating an "improved macrolide", the authors seem to have generated a somewhat compromised oxazolidinone. This interpretation is especially clear when noting that in no case does compound 13 outperform either of its parent compounds. The only exception of potential interest is compound 11, which demonstrates 2-fold improved activity over tedizolid in S. pyogenes and S. pneumoniae wildtype strains, though not against their resistant counterparts expressing erm genes.

Response: We agree wholeheartedly with these comments. I have also addressed some of these points in my responses to Reviewers 1 and 2, but I'll try to give a concise response here. Our presentation of several key points in the original manuscript needed improvement. The focus of this manuscript is the development and execution of the platform to design, synthesize, and evaluate these hybrid candidates. We view compounds **11** and **13** as "hit" compounds, not "lead" compounds, from a medicinal chemistry perspective. They were among the first compounds we synthesized in the development of this platform, and their ensemble of functional groups has not been optimized in a hit-to-lead fashion. It is entirely possible that further changes to the oxazolidinone, the macrolide, or the linker could improve potency further and reduce toxicity. In the years to come, we hope to reach "lead" compounds through extensive application of the platform. Also, compound **13** is already more active than linezolid in most strains (see the new Table S1 in the SI).

Secondly, even though many of its MICs are higher than **TDZ**, compound **13** is no longer highly susceptible to oxazolidinone ABC-F resistance (OptrA and PoxtA). Entries 15-17, 24, and 25 highlight this, with a maximum 2-fold increase in MIC for **13** (contrasted with **TDZ**, which has a maximum 16-fold difference). This has interesting implications for drug design of oxazolidinones to overcome these ribosome protection resistance mechanisms, which we have expanded upon in the revised manuscript.

A similar trend for **13** vs **TDZ**, but not quite as pronounced, can be found against strains G2576T mutations, where the MICs of **13** increase less than those of **TDZ** as the copy number of G2576T mutations in rRNA rises.

In each of these cases (mutations and ABC-F), **13** is *as active as **TDZ*** in the most resistant strains. With further medicinal chemistry optimization of these hybrids, we believe that the

activity **13** against WT strains can become competitive with **TDZ**, and the activity against oxazolidione-resistant strains will likely be superior to **TDZ**.

The way we presented these data in the first version of the manuscript certainly needed improvement. We have made several changes that we believe greatly improve the manuscript based on your comments.

In summary, this work represents a technically impressive and comprehensive study. The chemistry and structural biology are elegant, and the manuscript is well written. However, it is unfortunate that none of the hybrid compounds exceed the antimicrobial potency of either parent drug. This suggests that, at least in their current form, these conjugates may not offer clear therapeutic advantages over existing treatments such as azithromycin or tedizolid. The study could be significantly strengthened by evaluating the most promising compounds (e.g., 13) in in vivo infection models to test whether improved pharmacokinetics or selective toxicity might compensate for their diminished potency in MIC assays. Such experiments could uncover clinically meaningful advantages that are not apparent from the reported in vitro studies.

Response: These are certainly valuable experiments to be run down the line. We believe they are outside of the scope of the current work, which focuses on the platform development, structural insights, and initial hit generation of these hybrid series.

Additional Questions:
Quality of experimental data, technical rigor: Moderate

Significance to chemistry researchers in this and related fields: Top 10%

Broad interest to other researchers: Top 10%

Novelty: Moderate

Is this research study suitable for media coverage or a First Reactions (a News & Views piece in the journal)?: No

oc-2025-01046q.R2

Name: Peer Review Information for "Hybrid antibiotics targeting the bacterial ribosome"

Second Round of Reviewer Comments

Reviewer: 3

Comments to the Author

After carefully reading the revised version of the manuscript as well as the responses of the authors to the reviewer's comments, I can say that the authors improved the manuscript and addressed most of the critical points raised by this and other reviewers.

Author's Response to Peer Review Comments:

Summary of changes: Removed the Materials and Methods section from the manuscript, as all details are now in the Supporting Information. Added detailed descriptions of Supporting Information files at the end of the manuscript in ACS Central Science format.
